# Evolutionary “Crowdsourcing”: Alignment of Fitness Landscapes Allows for Cross-species Adaptation of a Horizontally Transferred Gene

**DOI:** 10.1093/molbev/msad237

**Published:** 2023-11-01

**Authors:** Olivia Kosterlitz, Nathan Grassi, Bailey Werner, Ryan Seamus McGee, Eva M Top, Benjamin Kerr

**Affiliations:** Biology Department, University of Washington, Seattle, WA 98195, USA; BEACON Center for the Study of Evolution in Action, East Lansing, MI 48824, USA; Biology Department, University of Washington, Seattle, WA 98195, USA; Biology Department, University of Washington, Seattle, WA 98195, USA; BEACON Center for the Study of Evolution in Action, East Lansing, MI 48824, USA; Department of Neuroscience, Washington University, St.Louis, MO 63110, USA; BEACON Center for the Study of Evolution in Action, East Lansing, MI 48824, USA; Department of Biological Sciences and Institute for Interdisciplinary Data Sciences, University of Idaho, Moscow, ID 83844, USA; Biology Department, University of Washington, Seattle, WA 98195, USA; BEACON Center for the Study of Evolution in Action, East Lansing, MI 48824, USA

**Keywords:** horizontal gene transfer, genomic background, epistasis, protein evolution, beta-lactamase, Enterobacteriaceae, adaptive landscape

## Abstract

Genes that undergo horizontal gene transfer (HGT) evolve in different genomic backgrounds. Despite the ubiquity of cross-species HGT, the effects of switching hosts on gene evolution remains understudied. Here, we present a framework to examine the evolutionary consequences of host-switching and apply this framework to an antibiotic resistance gene commonly found on conjugative plasmids. Specifically, we determined the adaptive landscape of this gene for a small set of mutationally connected genotypes in 3 enteric species. We uncovered that the landscape topographies were largely aligned with minimal host-dependent mutational effects. By simulating gene evolution over the experimentally gauged landscapes, we found that the adaptive evolution of the mobile gene in one species translated to adaptation in another. By simulating gene evolution over artificial landscapes, we found that sufficient alignment between landscapes ensures such “adaptive equivalency” across species. Thus, given adequate landscape alignment within a bacterial community, vehicles of HGT such as plasmids may enable a distributed form of genetic evolution across community members, where species can “crowdsource” adaptation.

## Introduction

Genes transferred horizontally between bacterial species evolve in dramatically different genomic backgrounds as they move between hosts (Redondo-Salvo et al. 2020). This contrasts with genes that evolve under strict vertical inheritance, where the genomic backdrop remains relatively constant over time. Although horizontal gene transfer (HGT) is prevalent and significant in bacterial evolution, the influence of host-switching on the evolution of genes that undergo HGT (hereafter “mobile genes”) has received little attention.

To assess the adaptive consequences of HGT, it is crucial to understand whether the fitness effects of mutations in mobile genes change depending on the host harboring the genes. We term this dependence a “gene-by-host interaction” (hereafter G × H), where mutational fitness effects depend in sign or magnitude on the entire host genomic background (for a more complete explanation of this terminology see supplementary section S1, Supplementary Material online). We emphasize that we focus on mutations within mobile genes (i.e. not within the host chromosome). The existence and form of G × H for such mutations may have evolutionary consequences for mobile genes, similar to those found in prior work on other interactions such as gene-by-gene (G × G) and gene-by-environment (G × E) interactions (Weinreich et al. 2006; Lindsey et al. 2013). For instance, if beneficial mutations in one host have similar effects in other hosts (i.e. negligible G × H), a species may effectively “crowdsource” the mobile gene's adaptive evolution. That is, a focal species that transfers the mobile gene to another species and subsequently reacquires it can benefit from adaptive genetic changes that occurred while in the second host. Conversely, if beneficial mutations in one host are dissimilar in others (i.e. non-negligible magnitude or sign G × H), the opportunity for HGT-driven crowdsourcing decreases.

**Fig. 1.**
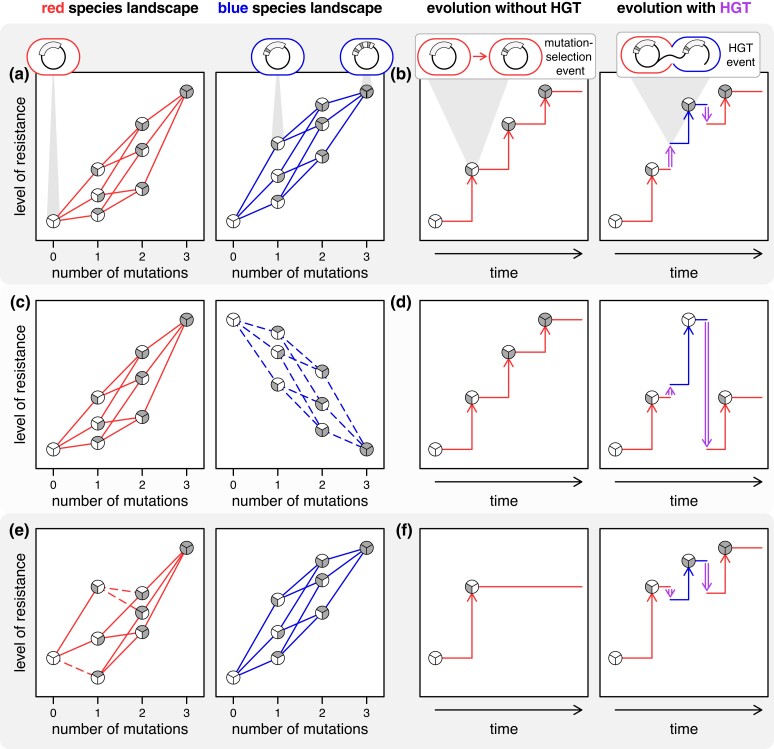
Effect of HGT on mobile gene evolution with hypothetical host-specific landscapes. We consider a landscape for 3 mutations in a mobile gene in 2 hosts (red and blue). a, c, e) The adaptive landscape is plotted as the resistance level of genotypes (taken as a proxy for fitness) as a function of the number of mutations on a wild-type (WT) background. Each of the 2^3^ = 8 genotypes is represented by a circle divided into “wedges” equal to the number of sites (3 in this case) where the evolved variant at a site is indicated by shading the corresponding wedge. The genotypes differing by a single mutation are connected by edges (lines colored to match the host). The effect of a mutation (beneficial or deleterious) is shown with a solid or dashed line, respectively. a) The landscapes of the red (left panel) and blue (right panel) host are reasonably well-aligned, as mutations have roughly similar effects across species and there are no mutations that exhibit opposite fitness effects in the 2 hosts. b) Assuming selection operates rapidly relative to mutation, we can represent each beneficial mutation's fixation as a step up in the level of drug resistance (vertical arrows). In an evolutionary trajectory within a population of the red host (left panel), after 3 mutational events, the population reaches the adaptive peak, from which all mutations are detrimental. Despite HGT (vertical purple double-ended arrow) to and from the blue host preceding and following the second mutational event (right panel), the population still reaches the adaptive peak because the blue and red host landscapes are aligned (part a). This scenario illustrates evolutionary crowdsourcing, where the red host can benefit from the transient adaptation in the blue host. c) The second scenario has rampant sign G × H where mutational steps are beneficial (solid lines) in the red host (left panel) but are deleterious (dashed lines) in the blue host (right panel). d) This is an example of evolutionary insourcing, where transient adaptation in the blue host hinders evolutionary progress in the red host. e) In this last example, there are only a few mutational steps with sign G × H, where the location of these mutations results in a suboptimal fitness peak in the red host landscape, which is absent in the blue host landscape. f) Evolution in the red host may lead to a suboptimal evolutionary endpoint (left panel). However, adaptation in the blue host can effectively release the red host from the suboptimal endpoint (right panel), a scenario that highlights evolutionary outsourcing.

In order to gauge the impacts of G × H on mobile gene evolution, we leverage the classical framework of the “fitness landscape,” which maps a network of mutationally connected genotypes to fitness (Wright 1932; de Visser and Krug 2014; Bank 2022). G × H manifests as differences in the landscape topography across hosts. To illustrate the evolutionary consequences of different forms of G × H, we explore a hypothetical example of 2 host species and 3 variant sites in a mobile gene. In Fig. 1a, the landscapes of the blue and red hosts are generally aligned with no instances of sign G × H (where the sign of a mutational effect is opposite in the 2 hosts). In this scenario, HGT between the hosts does not impact the red host's evolutionary end point relative to adaptation without HGT (Fig. 1b). These conditions enable evolutionary crowdsourcing, where the red host can take advantage of the transient adaptation in the blue host. In Fig. 1c, the 2 host landscapes are mirror images, indicating rampant sign G × H. Here, adaptation in the blue host is counterproductive to evolutionary progress in the red host (Fig. 1d). This scenario highlights evolutionary “insourcing,” where the red host makes more progress without HGT. A more subtle case is found in Fig. 1e where a handful of mutations exhibit sign G × H creating a suboptimal fitness peak in the red host landscape that is absent in the blue host landscape (there is actually pronounced G × G × H interaction here). In this case, adaptive evolution in the blue host explores additional regions of genotype space, and HGT introduces genetic variation from the blue host releasing the red host from a suboptimal endpoint. This scenario highlights evolutionary “outsourcing,” where HGT can qualitatively benefit the evolutionary trajectory in the red host relative to adaptation without HGT (Fig. 1f). These simple cases illustrate that comparing landscape topographies across hosts is the first step in determining how cross-species HGT may influence mobile gene evolution.

To explore how the landscape topography shifts with host background, it is necessary to uncover the existence and form of G × H by measuring the fitness effect of mutations in a mobile gene across different hosts. Previous studies found various forms of host-dependent effects for mutations introduced into chromosomal genes (Lind et al. 2010, 2017; Guerrero et al. 2019; Ogbunugafor and Eppstein 2019) However, the genes experiencing frequent host-switching (via HGT) are those residing on mobile genetic elements such as conjugative plasmids (Redondo-Salvo et al. 2020). Host-specific effects have been shown for the introduction of a plasmid (a type of G × H where the focal “mutation” involves going from a plasmid-free to plasmid-bearing state, see Alonso-Del Valle et al. 2021) and variation in plasmid gene content (e.g. G × H where the focal mutation involves a deletion of a gene, see Benz and Hall 2023). It is surprising that no attention, to our knowledge, has been given to host-specific effects of multiple intragenic mutations affecting the function of a protein encoded on a plasmid, where G × H interaction may be very relevant given that it could change how genes on mobile elements evolve over time.

Here, we experimentally constructed a portion of a mobile gene's landscape in 3 Enterobacteriaceae pathogens: *Escherichia coli*, *Salmonella enterica*, and *Klebsiella pneumoniae*. This gene, known as the *bla*_TEM_ gene, naturally resides on conjugative plasmids in enteric bacteria (Barlow 2009). It encodes a TEM-type beta-lactamase and has served as a model system for understanding protein evolution (Weinreich et al. 2006; Salverda et al. 2011). Specifically, we assembled a landscape featuring all combinations of 5 resistance-increasing mutations, building upon prior work by Weinreich et al. (2006) who used these mutations to asses intragenic interactions (i.e. G × G) in the *bla*_TEM_ gene in *E. coli*. Our study aimed to investigate the presence and nature of G × H, as well as the topographical alignment of this gene's landscape across different host species. Using evolutionary simulations involving adaptive walks on both empirically gauged and randomly generated landscapes, we assessed the relationship between cross-species landscape alignment and the effect of HGT on mobile gene evolution (e.g. crowdsourcing).

## Experimental Approach

**Fig. 2.**
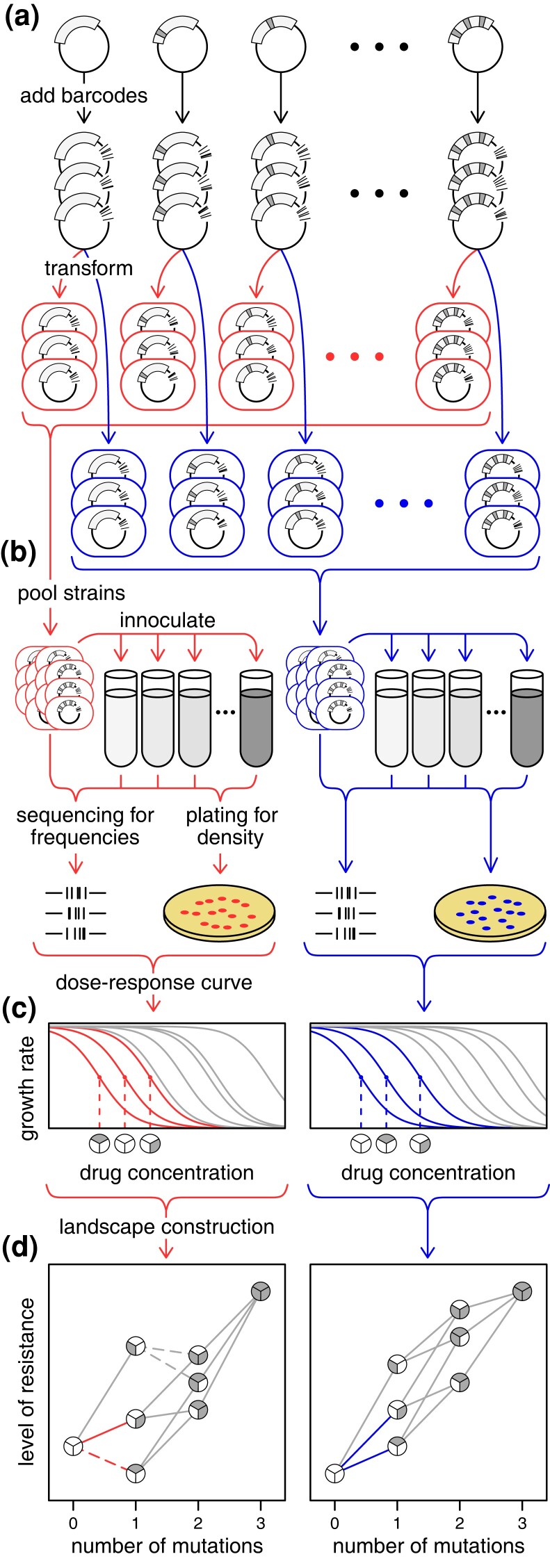
A multiplexed protocol for constructing host-specific landscapes. a) A focal gene (rectangular arc) on a plasmid is mutated (gray notches) to construct each plasmid genotype of interest. To track the plasmid genotypes in the experiment, each genotype is tagged with 3 unique barcodes (black notches) then transformed into each species (“red” and “blue” hosts). b) To assess the resistance level of each plasmid genotype, all transformants within a species are pooled to create the initial bacterial library and inoculated into an antibiotic gradient (the darker shades of gray correspond to higher antibiotic concentration in the growth medium). Samples are collected before and after incubation to determine barcode frequency using deep sequencing and the total population density using dilution plating. c) The growth rate specific to each plasmid genotype and drug concentration is calculated using the product of total population density and barcode frequencies associated with each plasmid genotype before and after selection at a given concentration. For each plasmid genotype, the estimated growth rates across the antibiotic gradient yield a dose response curve by fitting a log-logistic function, where the resistance level is given by the inflection point of the curve (indicated by the dashed vertical line). d) The landscape topography for each host is given by the collection of the set of plasmid genotypes’ resistance levels (the x axis values for inflection points in part c). The connections between the 3 highlighted genotypes from part (c) are shown in the host-specific color.

We used a high-throughput multiplexed assay to assess the host-specific landscape topography of a set of plasmid-borne antibiotic resistance *bla*_TEM_ genotypes. Our approach was inspired by recent advances that allow for parallel assessment of genotype fitness (Fowler and Fields 2014). Each plasmid genotype was mapped to the level of resistance it conferred in the host (a proxy for fitness), and these data points collectively formed the “resistance” landscape. To assess resistance levels, we first engineered each plasmid genotype and tagged it with unique barcodes before transforming it into a given host (Fig. 2a). Next, we pooled transformants to create the initial host library, and incubated this library in a series of tubes with increasing antibiotic concentrations (Fig. 2b). We approximated growth rates for each genotype at different antibiotic concentrations using pre- and post-selection cell counts and barcode frequencies (Fig. 2c). From these estimates, we generated a dose–response curve, using the curve's inflection point as our measure of resistance (supplementary Fig. S1, Supplementary Material online). Collectively, the resistance levels for the set of plasmid genotypes determined the topography of the landscape for the given host (Fig. 2d). By implementing this procedure across multiple bacterial species, we could compare landscapes between different hosts (see Materials and Methods for additional details).

## Results

### Experimental Host-Specific Landscape Construction Shows Minimal Gene-by-Host Interactions (G × H) in a Mobile Gene

**Fig. 3.**
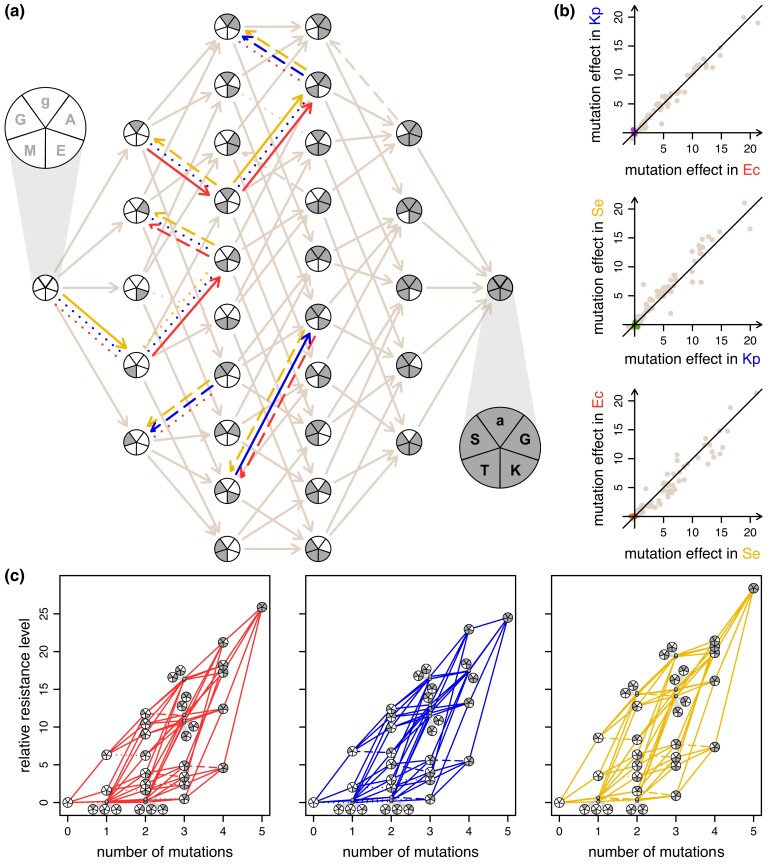
Multi-host landscapes of a mobile gene. a) The resistance landscape for the *bla*_TEM_ gene encoding a beta-lactamase was constructed for 3 enteric species: *Escherichia coli* (red), *Klebsiella pneumoniae* (blue), and *Salmonella enterica* (yellow), where 5 mutations (g4205a, A42G, E104K, M182T, and G238S) are shown as shaded wedges in each node. Lowercase or uppercase letters denote single nucleotide polymorphism in the promotor region or amino acid substitution, respectively. The white-wedged circle represents the TEM-1 genotype with low cefotaxime resistance, whereas the gray-wedged circle represents the most resistant genotype in all species. Mutational steps that exhibited sign G × H are shown as split multicolor edges with 1 arrow for each host (red, blue, and yellow), with beneficial, neutral, and deleterious effects denoted by solid, dotted, or dashed lines, respectively. Mutational steps with no sign G × H are shown with a single brown edge with the corresponding effect (solid, dotted, or dashed). In (b), the effect of each mutational step (80 in total) on the resistance level (akin to the slope in part c) is compared across each species pairing. The relative resistance level, $RRL(i,j)=\;\log_{\surd 2}\;(RL_{i}/RL_{j})$, involves comparing the resistance level (*RL* in μg ml^−1^) of a focal genotype *i* to a different genotype *j*. The plotted points compare the effects of mutations in the relevant species, where genotypes *i* and *j* differ by a single mutation. The mutational steps that exhibited sign G × H (split edges in part a) had small effects (purple, green, orange dots near the origin in the top, middle, and bottom panel, respectively) compared to the mutational steps exhibiting no sign G × H (brown dots). In (c), the landscapes for the 3 species were largely aligned given the low number of mutational steps that exhibited sign G × H and their small effects. Here, the RRL(i,j) is computed by comparing the resistance level (*RL*) of a focal genotype *i* to the TEM-1 genotype *j* (which has no mutations) for each respective host.

The plasmid genotype set that constituted the resistance landscape consists of all combinations of 5 particular mutations to the TEM-1 genotype of the *bla*_TEM_ gene (32 nodes in Fig. 3a). Of the 80 possible single-step mutations connecting 2 plasmid genotypes in our set, only 8 exhibited sign G × H across some combination of the 3 enteric species (these 8 mutations are indicated by the edges split into red, blue, and yellow pieces that connect distinct nodes in Fig. 3a). These mutations exhibiting G × H changed resistance by only small amounts (purple, green, and orange points near the origin in Fig. 3b). In contrast, all larger effect mutations exhibited similar increases in resistance across species (brown points in Fig. 3b). Thus, we concluded that the host-specific landscapes were generally aligned, as shown by the structural similarity among the diagrams in Fig. 3c.

### Simulations With Empirical Landscapes Reveal Evolutionary Crowdsourcing of a Mobile Gene

**Fig. 4.**
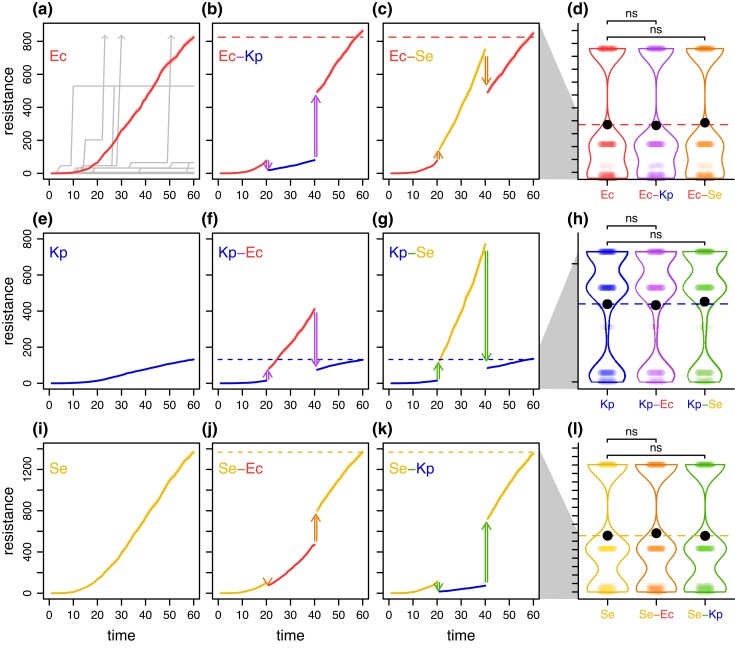
Evolutionary simulations using the empirical multi-host landscapes reveal evolutionary crowdsourcing. We employed evolutionary simulations based on empirically derived landscapes of the mobile *bla*_TEM_ gene (Fig. 3) to examine adaptive walks mediated by stochastic mutation and strong selection. In (a), replicate adaptive walks are depicted with gray lines. Subfigures (a, e, i) establish baseline conditions with no HGT, illustrating how the average resistance level increases (over 1,000 replicates with standard error given by the shading) due to gene evolution in *E. coli* (a), *K. pneumoniae* (e), and *S. enterica* (i). Subfigures (b, c, f, g, j, k) demonstrate that when the mobile gene evolved in a different species facilitated by HGT events (double-ended arrows), the evolutionary endpoint in the focal species was generally similar to that attained without HGT. In subfigures (d, h, l), we observed a pattern of evolutionary crowdsourcing given that the endpoint distributions between simulations without HGT (a, e, i) and with HGT (b, c, f, g, j, k) showed no significant differences according to Wilcoxon tests with Bonferroni corrections (*P* = 1, *P* = 0.79, *P* = 1, *P* = 0.57, *P* = 0.54, and *P* = 1, d to l from top to bottom). In each violin plot, the black dot represents the mean of the distribution. The color representations are the same as Fig. 3.

Topographical congruence between bacterial species potentially translates to crowdsourcing of the adaptive evolution of a mobile gene (as illustrated in Fig. 1a and b). However, as seen in Fig. 1e and f, even a few mutations exhibiting sign G × H can alter the evolutionary trajectory of a mobile gene. To assess the implications of our minor topographical differences, we simulated evolution as an adaptive walk (gray traces in Fig. 4a) on our empirically determined landscapes (Orr 2005; Fragata et al. 2019). Briefly, each simulation involved multiple rounds of stochastic mutation and selection for resistance. We tracked the average level of resistance without HGT (over 1,000 replicates) as a baseline for genetic evolution within a single host species (Fig. 4a, e, and i). To determine the effect of host-switching via HGT on gene evolution, we designed simulations over 3 distinct periods, where each transition to a different period coincided with a change in host (Fig. 4b, c, f, g, j, and k). In the first period, the mobile gene evolved in one species (hereafter the “focal” host) through several rounds of mutation and selection. An HGT event then moved the gene to another species (hereafter the “transient” host) commencing a second period. Finally, another HGT event returned the gene to the focal host initializing a third and final period. Despite time evolving in a transient species, the final level of resistance for the same total duration of evolution was statistically indistinguishable from the scenario with no HGT, a pattern observed for every possible focal-transient pair with our 3 species (Fig. 4d, h, and l). This result was robust to alterations of multiple simulation parameters (supplementary Figs. S2 to S4, Supplementary Material online). Therefore, with our empirically gauged landscapes, our species can effectively crowdsource the evolution of antibiotic resistance.

### Simulations With Artificial Landscapes Indicate Misalignment Impedes Evolutionary Crowdsourcing

**Fig. 5.**
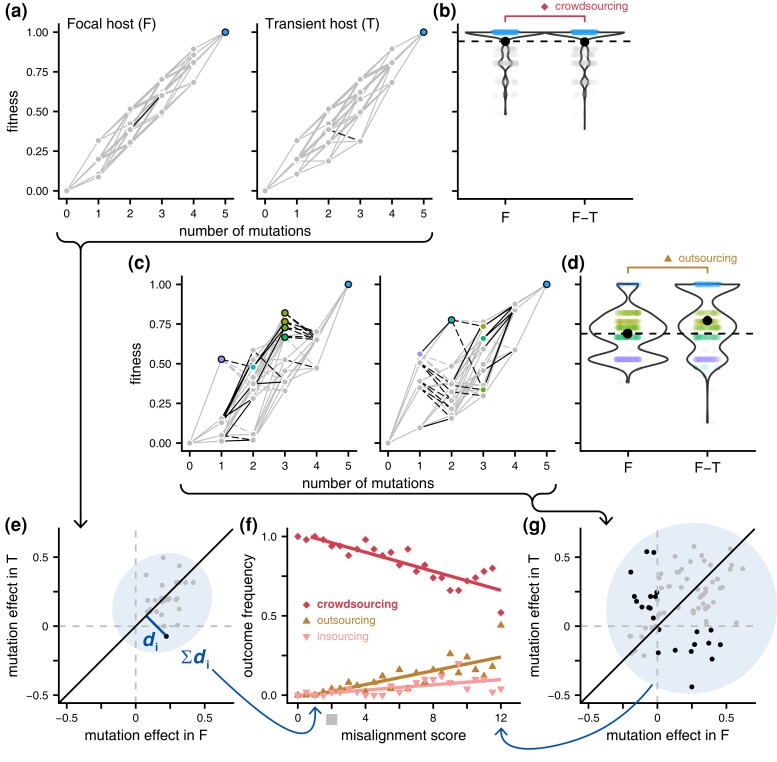
Landscape misalignment decreases the likelihood of evolutionary crowdsourcing. To investigate the influence of cross-species landscape misalignment on the role of HGT in gene evolution, we generated artificial landscapes with varying degrees of misalignment between 2 hosts: a focal host (F) and a transient host (T) (see Materials and Methods for our procedure). a) The landscape for each host included every combination of 5 mutations (the 2^5^ = 32 genotypes are shown as points here). The genotype with no mutations always had the lowest fitness and the genotype with all 5 mutations had the highest fitness. In this figure, genotypes that correspond to fitness peaks in at least one host's landscape are emphasized with distinct colors. The 2 landscapes here exhibit *low* misalignment, with a single common peak (light blue point) and only 1 mutational step that exhibits sign G × H (solid black line in the F landscape, dashed black line in the T landscape). b) The full distribution of fitness in the focal host at the end of evolutionary runs is shown for many replicate simulations. Using the same setup as in Fig. 4, these runs occurred either exclusively in the focal host (denoted “F”) or incorporated HGT events from the focal to the transient host and back, such that the middle third of the simulation occurred in the transient host (denoted “F-T”). For our aligned landscapes, these endpoint fitness distributions are very similar (with the global peak equally well represented both with, and without, HGT). This is a pattern of evolutionary crowdsourcing, symbolized by a red diamond. Conversely in (c), we present a landscape pair with high misalignment, characterized by multiple, mostly host-specific peaks (lavender, teal, and green-shaded points) and many mutational steps that exhibit sign G × H (black edges). In this case (d), the evolutionary simulations revealed evolutionary outsourcing, as marked by the tan triangle. Here, evolution in the focal host alone (left graph in part c) often led to evolutionary endpoints where the population became stuck on a suboptimal peak (lavender or green-shaded points). However, evolution with HGT between the focal and transient host led to a greater number of replicate runs reaching the global peak (i.e. more weight in the part of the distribution corresponding to the light blue dots in “F-T” vs. “F”). e) To calculate cross-species landscape misalignment, we followed 3 steps: (i) we plotted the effect of every mutation in one host as a function of its effect in the other (note in some cases, data points overlap), (ii) calculated the distance between the point for mutation *i* and the identity line ($d_{i}$), and (iii) summed these distances across all mutations ($\sum d_{i}$). For the aligned landscapes in part (a), most points do not stray far from the identity line and the misalignment score is low (i.e. this graph corresponds to a low position on the x axis in part f). g) On the other hand, the misaligned landscapes in part (c) yields points scattered further away from the identity line, leading to a high misalignment score (a high position on the x axis in part f). f) Including the 2 pairs of landscapes shown in parts (a) and (c), we ran evolutionary simulations on 1,250 pairs of landscapes with a variety of misalignment scores. These landscape pairs were grouped into bins of 0.5 increments (see Materials and Methods for details). For each bin, we tracked the proportion of landscapes pairs for which the incorporation of HGT generated crowdsourcing (as in part b), insourcing, or outsourcing (as in part d). We observe a significant inverse relationship between landscape misalignment and the frequency of evolutionary crowdsourcing. The bin for the misalignment scores for our empirical landscapes is indicated on the x axis with a gray square.

To delve further into the interplay between landscape alignment across species and the impact of HGT on mobile gene evolution, we extended our simulation framework to randomly generated pairs of fitness landscapes. These artificial landscapes retained key features of the empirically derived landscapes to facilitate direct comparison. Specifically, we preserved the number of mutations, fixing the genotype with no mutations at the lowest fitness level and the genotype with all 5 mutations at the highest. As a baseline, we generated a single non-epistatic (smooth/additive) landscape where the fitness effects of each mutation were randomly assigned and independent of context. This additive landscape served as the common starting point from which the pair of landscapes corresponding to the 2 host species were generated. To introduce variation between the hosts (i.e. G × H), we perturbed the fitness values for a subset of randomly selected genotypes, independently for each host species. This approach resulted in pairs of landscapes with varying degrees of misalignment, ranging from well-aligned (as depicted in Fig. 5a and e) to poorly aligned (Fig. 5c and g). The fitness effects of all mutations in one species can be represented as a function of their corresponding effects in the other host species using a scatterplot (Fig. 5e and g). If a point corresponding to a mutation lands on the identity line, it must have equivalent effect across hosts. However, the displacement of a point from the identity line indicates the presence of G × H, signifying a disparate effect of the relevant mutation across species. To quantify total landscape misalignment, we used a simple metric that summed the perpendicular distances of points from the identity line. Simulations both with and without HGT on different pairs of landscapes yielded evolutionary outcomes of insourcing, outsourcing (Fig. 5d), and crowdsourcing (Fig. 5b). Notably, as the degree of misalignment between the pair of landscapes increased, we observed a significant reduction in the frequency of crowdsourcing outcomes (Fig. 5f, *P* < 10^−6^ by a permutation test described in supplementary Supporting Material, Supplementary Material online). Furthermore, our empirically derived landscapes fell within the range of misalignment values where the crowdsourcing outcome was most likely to occur (gray square in Fig. 5f).

## Discussion

HGT serves as a critical driver of bacterial evolution, enabling organisms to rapidly adapt to new challenges by acquiring genetic elements from other species. Despite the extensive research recognizing the prevalence of HGT and the relevant gene cargo transferred, the consequences of host-switching for the evolution of the transferred genes themselves (i.e. mobile genes) has been underexplored. Our study, to our knowledge, provides the first examination on the potential role of gene-by-host interactions (G × H) in the evolution of proteins encoded by mobile genes. Through our conceptual framework and simulation analysis, we demonstrated that the topographical congruence of landscapes of different host species strongly influences the evolutionary trajectories of mobile genes. Through our empirical case study of a mobile gene across 3 bacterial pathogens, we found minimal G × H which enabled evolutionary “crowdsourcing.” Our results not only substantiate the potential for HGT to serve as a conduit for collaborative evolution among bacterial species, but also highlight the role of landscape alignment in shaping the adaptive consequences of HGT.

Our study has limitations in both the landscape reconstruction and evolutionary simulation. First, we analyzed a limited set of mutations in 1 gene in 3 closely related species. Thus, our results may not be applicable to all mutations, genes, or species. Second, our landscapes are based on resistance, which correlates strongly with competitive fitness for certain environments (Gullberg et al. 2011; Toprak et al. 2011; Artemova et al. 2015; Schenk et al. 2022). However, a genotype's fitness can be influenced by factors other than resistance (e.g. baseline growth rate in drug-free conditions) and these factors may not correlate with drug resistance (Chevin 2010; Concepción-Acevedo et al. 2015; Ogbunugafor et al. 2016; Das et al. 2020). Third, our evolutionary simulations made several simplifying assumptions; e.g. a series of selective sweeps comprised each adaptive walk and host-switching occurred at a few defined times. However, natural bacterial communities are often more complex, with multiple genotypes competing within and across species, and continual potential for transfer. The outcomes of these competitions as well as the opportunities for HGT depend on the distribution of the relevant species across a potentially heterogeneous environment (e.g. a multispecies biofilm in a drug gradient). Additionally, the simulations did not account for some unique plasmid features such as multiple copies per cell, fitness costs, and basic rates of conjugation and plasmid loss. These features can vary with host context (De Gelder et al. 2007; Dimitriu et al. 2019; Kosterlitz et al. 2022) and may influence HGT opportunities and competitive outcomes. Lastly, while our misalignment metric shows a significant correlation with the evolutionary outcome, it represents just one of many possible approaches (e.g. an alternative metric would be the fraction of mutations exhibiting sign G × H, see de Vos et al. 2015). In future work, a more refined cross-host alignment metric could be formulated particularly if landscape features like ruggedness (or higher-order interactions, such as G × G × H) prove to be predictive of the impact of HGT on mobile gene evolution. More generally, future work should consider all of the above limitations to enrich empirical, theoretical, and simulation frameworks.

Despite these noted caveats, we highlight the possibility that evolutionary adaptation of a mobile gene can be a cosmopolitan affair in a microbial community, where the progress made in one species translates to progress in another. We emphasize that the availability of widespread evolutionary crowdsourcing through HGT will depend on the prevalence and magnitude of G × H and thus overall landscape misalignment (Fig. 5). Interestingly, for our focal mobile gene, the frequency of sign G × H and the effect sizes of all G × H interactions were surprisingly low given the documented number of context-dependent mutations in other studies (De Gelder et al. 2007; Apjok et al. 2019; Guerrero et al. 2019; Ogbunugafor and Eppstein 2019; Gama et al. 2020; Dunn et al. 2021; Benz and Hall 2023). This pattern highlights a connection to the “complexity hypothesis,” which suggests that proteins encoded by genes experiencing higher rates of HGT are less connected to other proteins in the cell (Jain et al. 1999; Novick and Doolittle 2019). This could result in fewer opportunities for host-dependencies and less G × H for these more “modular” mobile genes. To further explore the relationship between the rate of HGT and the availability of evolutionary crowdsourcing, it will be necessary to construct landscapes for additional genes undergoing different rates of HGT across the same set of species.

Our findings expand upon the existing body of knowledge surrounding context-dependent genetic interactions, where the effects of mutations can vary based on specific contextual factors (Eguchi et al. 2019). These contextual factors can be of different forms, such as variations within the same gene (intragenic epistasis), within the same genome (intergenic epistasis), in a different genome (intergenomic, or interspecific, epistasis), or in environmental states (G × E interaction) (Gillespie 1984; Orr 2002; Wade 2007; de Vos et al. 2013; Flynn et al. 2013; Lindsey et al. 2013; Bank et al. 2016; Yi and Dean 2019; Bank 2022; Gupta et al. 2022). In our study, we focus on G × H interactions, which can be interpreted in multiple ways. These could be viewed as a specific type of G × G, where the host chromosome is the contextual (G) factor. Alternatively, G × H can be conceptualized as a form of G × E, where the host serves as the environmental context (E) for the mobile gene. Our simulation was designed with the G × E perspective in mind, treating rare HGT events as environmental changes (for a more complete explanation of the connections between G × H, G × G, and G × E, see supplementary section S2, Supplementary Material online). However, G × H interactions resist easy categorization within either the G × G or G × E frameworks. For a mobile gene encoded on a plasmid, HGT can dramatically alter the host context by moving the plasmid to an entirely different species. This is in sharp contrast to the subtler changes caused by mutations, which align with the conventional G × G framework and are often associated with plasmid–host coevolution. These scenarios differ not only in the scale of contextual alteration but also in the underlying processes that drive these changes—specifically, mutation versus HGT. Each of these processes is characterized by unique rates, mechanisms, and ecological dependencies. Therefore, recognizing the unique characteristics of G × H could pave the way for the development of more comprehensive and predictive models for gene evolution.

In summary, we introduced a novel framework to investigate the molecular evolution of mobile genes—a highly relevant subset of genes evolving with an additional mode of genetic inheritance: HGT. We found that for a small set of mutations in a common mobile gene, the landscape topography and thus evolutionary outcomes are largely aligned across closely related species. These findings suggest that adaption of mobile genes in one species can translate to adaptation for another species. This suggests that conjugative plasmids and other vehicles of cross-species HGT can enable a distributed form of genetic evolution across bacterial communities, where any particular species can draw upon genetic variation from other community members and adapt through “crowdsourcing.”

## Materials and Methods

### General Reagents

Unless otherwise noted, all enzymes and related buffers were obtained from New England Biolabs. Plasmid isolation kits were obtained from Qiagen. DNA cleaning and gel extraction kits were obtained from Zymo Research. Oligonucleotide primers were obtained from Integrated DNA Technologies. Sanger sequencing was conducted by GENEWIZ from Azenta Life Sciences.

### Genotype Construction and Barcoding

We mutated the pBR322 plasmid, which contains the *bla*_TEM_ and *tetA* genes, using a Site-Directed Mutagenesis Kit. Plasmid maintenance was ensured by supplementing the culture medium with 15 μg ml^−1^ tetracycline. The starting genotype for the *bla*_TEM_ gene was TEM-1, and all combinations of 5 mutations (g4205a, A42G, E104K, M182T, and G238S) were generated using custom primers (supplementary table S1, Supplementary Material online). All mutations (supplementary table S2, Supplementary Material online) were confirmed with Sanger sequencing (supplementary table S3, Supplementary Material online). Each beta-lactamase genotype was associated with 3 unique molecular barcodes. For barcoding, we modified the pBR322 backbone to incorporate Nsil and NcoI restriction sites downstream of the *bla*_TEM_ gene. Double-stranded barcoded fragments were prepared using 2 oligonucleotides (supplementary table S4, Supplementary Material online) and inserted into the digested vector through ligation. 3 colonies were sequenced to confirm barcode identity for each genotype, resulting in a library of 96 engineered plasmids. To create host-specific libraries, the 96 engineered plasmids were transformed into each host, and the resulting strains were pooled and stored in 1 ml aliquots at −80 °C in 15% (v/v) glycerol for later use.

### Bacterial Strains, Media, and Culture Conditions

We used 3 Enterobacteriaceae host species: *E. coli* DH10B (Durfee et al. 2008), *K. pneumoniae* Kp08 (Jordt et al. 2020), and *S. enterica* serovar *typhimurium* LT2 (McClelland et al. 2001), abbreviated as Ec, Kp, and Se, respectively. All strains were cultured at 37 °C in lysogeny broth (LB).

### Pooled Competitions Assays

Resistance levels conferred by the *bla*_TEM_ genotypes were estimated using a modified minimum inhibitory concentration assay (Wiegand et al. 2008). The library stocks were thawed, grown in 50 ml of growth medium with 15 μg ml^−1^ tetracycline, and diluted to an initial density close to 10^5^ cells ml^−1^ estimated through dilution plating in triplicate (supplementary table S5, Supplementary Material online). To start the competition assays, 2.5 ml of diluted library was inoculated into 41 test tubes supplemented with escalating cefotaxime (CTX) concentrations using √2-fold dilutions from 2,049.37 up to 0.00393 μg ml^−1^. After overnight incubation, samples from tubes with visible growth were taken for library amplification and sequencing, and final cell densities were determined using dilution plating in triplicate (supplementary table S6, Supplementary Material online).

### Library Amplification and Sequencing

Plasmid DNA was extracted from cell pellets stored at −20 °C, and the barcode region was PCR-amplified using backbone-homologous primers (supplementary table S7, Supplementary Material online). Amplicons were purified and further amplified with unique indexing primers. Sequencing was performed on the Illumina NextSeq500 platform.

### Library Sequence Analysis, Genotype Growth, and Genotype Resistance

We processed the FASTQ files to extract 18 bp barcodes, cluster them using Bartender (Zhao et al. 2018), match them to the Sanger results during the cloning step (supplementary table S8, Supplementary Material online). For a given genotype (*g*) at a particular drug concentration (*c*), the corresponding growth rate ($\mu_{g}^{c}$) was calculated using the estimated initial ($n_{*}^{c}(0)$) and final ($n_{*}^{c}(T)$) cell densities (supplementary tables S5 and S6, Supplementary Material online) along with initial ($b_{g}^{c}(0)$) and final ($b_{g}^{c}(T)$) barcode frequencies using the following equation:


$$\mu_{g}^{c}=\frac{1}{t_{c}}\ln\frac{n_{*}^{c}(T)b_{g}^{c}(T)}{n_{*}^{c}(0)b_{g}^{c}(0)}$$

where $t_{c}$ is the approximate period of growth under drug concentration *c* (see supplementary section S3, Supplementary Material online for details).

For the 3 barcodes of each genotype, we eliminated the one most deviant in growth rate across the drug concentration gradient (determined by summing the squares of the pair-wise differences in growth rates). For the 2 remaining barcodes, we fit a 3-parameter log-logistic dose–response curve (a few examples are shown in supplementary Fig. S1, Supplementary Material online) using the drc package in R (Ritz et al. 2015). We note that a fixed “no-growth” baseline improved curve fitting across genotypes. The 3-parameter estimates for the dose–response curve (upper asymptote, steepness, and inflection point) for each barcode-genotype-species combination are given in supplementary table S9, Supplementary Material online. We used the inflection point of the curve as a proxy for the resistance level. Host-specific landscapes were constructed by comparing the level of resistance of neighboring genotypes (Fig. 3). Specifically, we calculated the relative resistance level, $RRL(i,j)=\;\log_{\surd 2}\;(RL_{i}/RL_{j})$ by comparing the resistance level (*RL* in μg ml^−1^) of a focal genotype *i* to a different genotype *j* where genotypes *i* and *j* differ by a single mutation. Based on the *RRL*, mutational steps were categorized as beneficial, deleterious, or neutral (solid, dashed, or dotted lines in Fig. 3a and c).

### Validation of Resistance Levels Through Standard Minimum Inhibitory Concentration (MIC) Assays

We conducted conventional low-throughput minimum inhibitory concentration (MIC) assays to validate the resistance levels estimated from our high-throughput approach. Bacterial strains were inoculated in triplicate into microtiter wells, each supplemented with increasing concentrations of CTX. After overnight incubation, the MIC values were determined as the average lowest concentration at which no visible bacterial growth was observed across the technical replicates. It is noteworthy that our high-throughput approach, which employs pooled library competition assays across a concentration gradient, affords greater resolution in measuring bacterial resistance compared to traditional MIC assays. Our methodology provides a more granular measure of resistance by fitting a dose–response curve to a quantitative measure (i.e. growth rate), in contrast to MIC assays that produce a binary output (i.e. growth vs. no growth). The increased granularity is particularly advantageous for detecting subtle changes in resistance, such as those arising from mutations of small effects. Another advantage to our methodology is that all strains of interest are being exposed to the same drug environment (i.e. pooled in the same test tube). This contrasts with MIC assays where slight variation in the preparation of the drug gradient across strains and replicates can add experimental error. In such a case, different genotypes (as well as different replicates of the same genotype) can be exposed to different concentrations, which can decrease the precision in the measured level of resistance. Importantly, despite these methodological differences, we observed a strong correlation between the resistance levels identified by our high-throughput assays and those derived from standard MIC assays (supplementary Fig. S5, Supplementary Material online).

### Evolutionary Simulations

Our simulations modeled gene evolution over defined periods in different host species as an adaptive walk (Fragata et al. 2019) (see supplementary Methods, Supplementary Material online for more details). Within each period, mutation and selection occurred at discrete time steps. Genotypes with higher resistance had a higher chance of becoming fixed, and the likelihood of this fixation was influenced by factors such as mutation rate, population size, and the fitness differences (supplementary section S4, Supplementary Material online). Our simulation ignores the potential for multiple genotypes to coexist, as each selective replacement involves the fixation of a more resistant genotype over a time step. However, we note that the immediate fixation of the most resistant variant (from a set of mutants stochastically generated from a single genotype) reasonably mimics the iterative step within standard drug-gradient directed-evolution schemes (Salverda et al. 2010; Packer and Liu 2015; Salverda et al. 2017). Simulation parameters, such as the mutation rate and the number of discrete time steps, were varied to assess their impact (supplementary table S10, Supplementary Material online). We demonstrated that the trends shown for the *bla*_TEM_ gene maintained across parameter sweeps in mutation rate (supplementary Fig. S2, Supplementary Material online), cumulative time (supplementary Fig. S3, Supplementary Material online), and number of simulation replicates (supplementary Fig. S4, Supplementary Material online). Significance tests on the distributions of endpoint resistance were performed using Wilcoxon tests with Bonferroni corrections to determine whether HGT had a positive, negative, or neutral effect on the evolutionary outcomes.

### Artificial Landscape Analysis

To investigate the impact of HGT on gene evolution across different host landscapes, we created artificial fitness landscapes for pairs of host species. As in Fig. 3a, each landscape involved all combinations of 5 mutations. For each pair of artificial landscapes, we started with a common additive “baseline” landscape, in which the effect of a mutation at locus *i*, which we label $\varepsilon_{i}$, was selected randomly between 0 and $\varepsilon_{\max}$ (we used the arbitrary value of $\varepsilon_{\max}=0.2$). We then divided the fitness of every genotype by $\sum_{i}\varepsilon_{i}$, such that the fitness of the genotype with no mutations was always 0 and the fitness of the genotype with all mutations was always 1. This baseline landscape was copied to generate the 2 landscapes for each host, which underwent further manipulation. Misalignment between host landscapes was introduced by randomly perturbing fitness values for a subset of genotypes. The new fitness values were picked from a normal distribution centered around the original fitness values (and truncated at 0 and 1). For this, we excluded the genotype with no mutations and the genotype with all 5 mutations, leaving 60 intermediate genotypes for potential reassignment (30 genotypes in each of the 2 hosts). By plotting the effect of every mutation in one host as a function of its effect in the other host, the total misalignment was assessed by summing the perpendicular distances of all the mutational points from the identity line in the scatterplot. Using this framework, we ran 500 adaptive walks without HGT and 500 with HGT on each host landscape. The endpoint distributions were statistically compared as previously described to assess whether HGT had a positive (outsourcing), negative (insourcing), or neutral (crowdsourcing) effect on the evolutionary outcomes. To assess the relationship between landscape misalignment and evolutionary outcomes, we generated a large number of artificial landscape pairs and randomly sampled 50 pairs with a misalignment score that fell into each of 25 bins of increasing scores. We then ran simulations on each of the 1,250 landscape pairs and assessed the significance of the trend between misalignment and evolutionary outcome using a permutation test.

## Supplementary Material

msad237_Supplementary_Data

## Data Availability

Extra details on the materials and methods are available in the supplementary Supporting Material, Supplementary Material online. The raw data and code can be found at https://github.com/livkosterlitz/crowdsourcing and archived at https://doi.org/10.5281/zenodo.10045641.
