# Supplementary material for: Evolutionary “Crowdsourcing”: Alignment of Fitness Landscapes Allows for Cross-species Adaptation of a Horizontally Transferred Gene
Source: Mol Biol Evol. 2023 Nov 1;40(11):msad237. doi: 10.1093/molbev/msad237 (PMC10657783; doi:10.1093/molbev/msad237)
Supplement: msad237_Supplementary_Data [file msad237_supplementary_data.pdf]

Evolutionary "crowdsourcing": alignment of fitness landscapes allows for cross-species adaptation of a horizontally transferred gene

Olivia Kosterlitz[1,2,*], Nathan Grassi[1], Bailey Werner[1], Ryan Seamus McGee[2,3], Eva M. Top[2,4], Benjamin Kerr[1,2,*]

Author affiliations

[1] Biology Department, University of Washington, Seattle, WA, USA.

[2] BEACON Center for the Study of Evolution in Action, East Lansing, MI, USA.

[3] Department of Neuroscience, Washington University, St. Louis, MO, USA.

[4] Department of Biological Sciences and Institute for Interdisciplinary Data Sciences, University of Idaho, Moscow, ID, USA.

* corresponding authors: Olivia Kosterlitz (livkost@uw.edu) and Benjamin Kerr (kerrb@uw.edu)

Keywords

Horizontal gene transfer, genomic background, epistasis, protein evolution, beta-lactamase, *Enterobacteriaceae*, adaptive landscape

Supporting Material

Extended Materials and Methods

We provide additional details on our materials and methods organized into subsections (in *italics*). These subsections are placed under the subheading that corresponds to the relevant section in the main text materials and methods (in **bold).** We note that not all main text sections have an extended materials and methods subsection here.

Genotype construction and barcoding

DNA fragment preparation for barcoding

We digested the mutated plasmids with NsiI and NcoI at 37°C for 1 h and the restriction enzymes were heat inactivated at 65°C for 20 min. We isolated the digested vector backbone using a gel extraction kit and purified the DNA. We next prepared the double-stranded barcoded fragments to be inserted by ligation using two oligonucleotides: (1) an oligonucleotide with 18bp random barcode sequences nested between the NsiI and NcoI cut sites to be used in directional cloning, and (2) a shorter priming oligonucleotide containing homology to the barcode oligonucleotide. These two oligonucleotides are listed in SI Table 4. To construct the double-stranded barcode fragment, we mixed 1 µL of each oligonucleotide with 5 µL of CutSmart Buffer and 47.5 µL of ddH_2O and annealed these oligonucleotides together by incubating at 98°C for 3 min followed by a ramping down to 25°C at -0.1°C/s. After annealing, we added 1 µL of Klenow polymerase (exonuclease negative) and 1.65 µL of 1mM dNTPs to make the barcode fragment double stranded by incubating at 25°C for 15 min, 75°C for 20 min, and then a ramping down to 37°C at -0.1°C/s. We digested the double-stranded fragment using the same enzymes and protocol for digesting the vector backbone described above. The digested barcoded fragment was then purified. The digested vector and barcoded fragment were ligated at 21°C for 30 min, the enzymes were heat inactivated at 65°C for 10 min and the circular products were transformed into *E. coli*.

Library amplification and sequencing.

Amplicon PCR

The barcode region was amplified using the primers homologous to the plasmid backbone (SI Table 7) with the following conditions: 95°C for 3 min, five cycles of 98°C for 15 s, 65°C for 15 s, 72°C for 30 s, and 72°C for 1 min. Amplicons were then purified with AMPure XP beads (Beckman Coulter) at 1:1 ratio.

Multiplexing PCR

Each sample's purified PCR product was amplified with a unique pair of forward and reverse indexing primers plus SyberGreen with the following PCR conditions on a miniOpticon (Bio-Rad): starting with 95°C for 3 min, fifteen cycles of 98°C for 20 s, 60°C for 15 s, 72°C for 30 s, and finishing with 72°C for 2 min. Using the relative fluorescence units, the amplicons were mixed, gel extracted, quantified by Qubit fluorometry, and sequenced on the Illumina NextSeq500 platform by the Microbial Genome Sequencing Center using custom sequencing primers.

Library sequence analysis, genotype growth, and genotype resistance.

Lower asymptote parameter in dose-response curves

Given that there was a slight lift of the lower asymptote (i.e., a non-zero value), we took the lower asymptote parameter average for each species using approximately half of the genotypes with the lowest resistance (n = 17). For each barcode, we then used its species-specific average as a lower asymptote parameter and determined the three additional parameters (upper asymptote, steepness, and inflection point) giving the best fit log logistic dose-response curve (where the resistance level was the inflection point parameter). If the lower asymptote was zero, then our resistance level would be an IC50 value.

Assigning effects of mutations

To determine the effect of a mutational step, we leveraged our experimental design where two barcodes are associated with each genotype which served as internal replicates in the competition. Therefore, if the resistance estimates (from both barcodes) for the single mutant neighbor were both higher than the focal genotype's estimates (from both of its barcodes), the mutational step was beneficial. If the estimates for the single mutant neighbor were both lower than the focal genotype's estimates, the mutational step was deleterious. If the estimates for the single mutant neighbor overlapped with the focal genotype, the mutational step was neutral.

Evolutionary Simulations

Mutation and selection

We modeled the evolutionary trajectory of a gene as a sequence of single genotype states, where an update reflecting the action of both mutation and selection was performed at each time step (i.e., an adaptive walk). Specifically, we consider a finite population of descendant individuals to be generated from the ancestral genotype with random mutation. Thus, the members of the descendant population will include the ancestral genotype (resulting from a lack of mutation) and a subset of genotypes that differ from the ancestral genotype by a single mutation (each resulting from a mutational event). From this random set of mutants, the most resistant genotype is picked. If the most resistant mutant from this subset is more resistant than the ancestral genotype, then the population fixes on this mutant genotype (making it the new ancestral genotype for the next time step).

Technically, we compute the probability of each genotype j (ancestral and neighboring mutant genotypes) having the highest resistance among a set of descendants given that genotype i is the current ancestor, and we draw the population's next genotype state from this distribution. This set of probabilities depend on mutation rate, population size, and the resistance levels of the ancestor and mutant neighbors (see Supplementary section 4 for details on calculating the probabilities). We note that at some time steps the most resistant genotype may have been the ancestral genotype if no mutants were generated (which becomes more likely at low mutation rates) or if no generated mutant was more resistant than the ancestor (which becomes more likely when fewer mutational neighbors improve resistance). We also note that there is non-zero probability that any mutant genotype with greater resistance than the ancestral genotype can fix (even if other mutants have higher resistance). Generally, however, mutant neighbors with the highest resistance will have the highest probability of fixing. This stochastic process has connections to previously discussed adaptive walks as parameters attain extreme values. At extremely low mutation rates, our adaptive walk becomes a "random adaptive walk" (in which a neighbor with higher fitness than its ancestor is chosen at random), while at extremely high mutation rates, it becomes a "greedy adaptive walk" (in which the neighbor with highest fitness is selected, if greater than the ancestor) (Grewal et al. 2018).

Horizontal gene transfer events

Each evolutionary simulation comprised periods of host-specific evolution, in which the focal gene evolved for a specified number of time steps inside a single host species (SI Table 10). An HGT event was defined as a switch in the host of the evolving gene, which occurred at specified time steps (SI Table 10). Therefore, a simulation for a gene evolving in different hosts over time consisted of distinct periods of single-species evolution linked together by HGT events. If there was sign G×H or magnitude G×H, in which the rank ordering (based on resistance) of the mutational neighbors changed, the evolutionary trajectory of a gene could be affected by HGT. Thus, sign or magnitude G×H could enable HGT to impact the path and endpoint of adaptive evolution.

Empirical landscape simulation specifics

The empirically determined host-specific landscapes provided the information about the beneficial mutants available for each focal genotype (number and ranking). Since each genotype for the bla_{TEM} gene had multiple (replicate) estimates for its level of resistance (SI Table 9), at each time step, the resistance for each genotype was sampled randomly from the set of estimates. Therefore, for each time step of the

simulation, the host landscape could potentially shift; however, these shifts were small given that the variance across the resistance estimates in our assay were generally very low.

Parameter settings

Each simulation examined a parameter in isolation by manipulating the relevant parameter (mutation rate, cumulative time, and number of simulation replicates). For the "baseline" simulations, we used a "baseline" set of parameters (mutation rate of 5 x 10^{-5}, populations size was 1000, and the number of replicate simulations was 1000). For treatments with HGT, the middle period was always one third of the cumulative time.

Artificial landscape analysis

Permutation test for the relationship between misalignment and crowdsourcing

For each misalignment score bin value, m, there is a proportion of landscape pairs, p, for which evolutionary simulations indicate a crowdsourcing pattern. For ease of discussion, imagine ordering the bin values as follows: $1, 2, 3, \dots N$. Thus, we can arrange the misalignment scores as a vector $\boldsymbol{m} = (m_1, m_2, m_3, \dots m_N)$ and the crowdsourcing proportions as a vector $\boldsymbol{p} = (p_1, p_2, p_3, \dots p_N)$. In our observed data, the value of m_i is paired with the value of p_i. We define $\beta_{\boldsymbol{m},\boldsymbol{p}}$ as the slope of the best-fit line for the data.

Our null hypothesis is that the proportion of crowdsourcing outcomes does not change with misalignment (i.e., that the slope of the relationship between m and p is zero). In such a case, our $\beta_{\boldsymbol{m},\boldsymbol{p}}$ value should not be too extreme compared to surrogate best-fit slopes $\beta_{\boldsymbol{m},\boldsymbol{p_r}}$ where the vector $\boldsymbol{p_r} = (p_{r_1}, p_{r_2}, p_{r_3}, \dots p_{r_N})$ is a random permutation of $\boldsymbol{p}$, i.e., where the index vector $\boldsymbol{r} = (r_1, r_2, r_3, \dots r_N)$ is a random permutation of the vector $(1,2,3, \dots N)$. For each analysis, we generate 100,000 $\boldsymbol{p_r}$ vectors and we define the fraction of surrogate $\beta_{\boldsymbol{m},\boldsymbol{p_r}}$ slopes that are strictly less than $\beta_{\boldsymbol{m},\boldsymbol{p}}$ to be P. This yields our P-value for a one-sided permutation test (i.e., where the alternative to our null hypothesis is that the slope of the relationship between m and p is negative).

Supplemental Figures

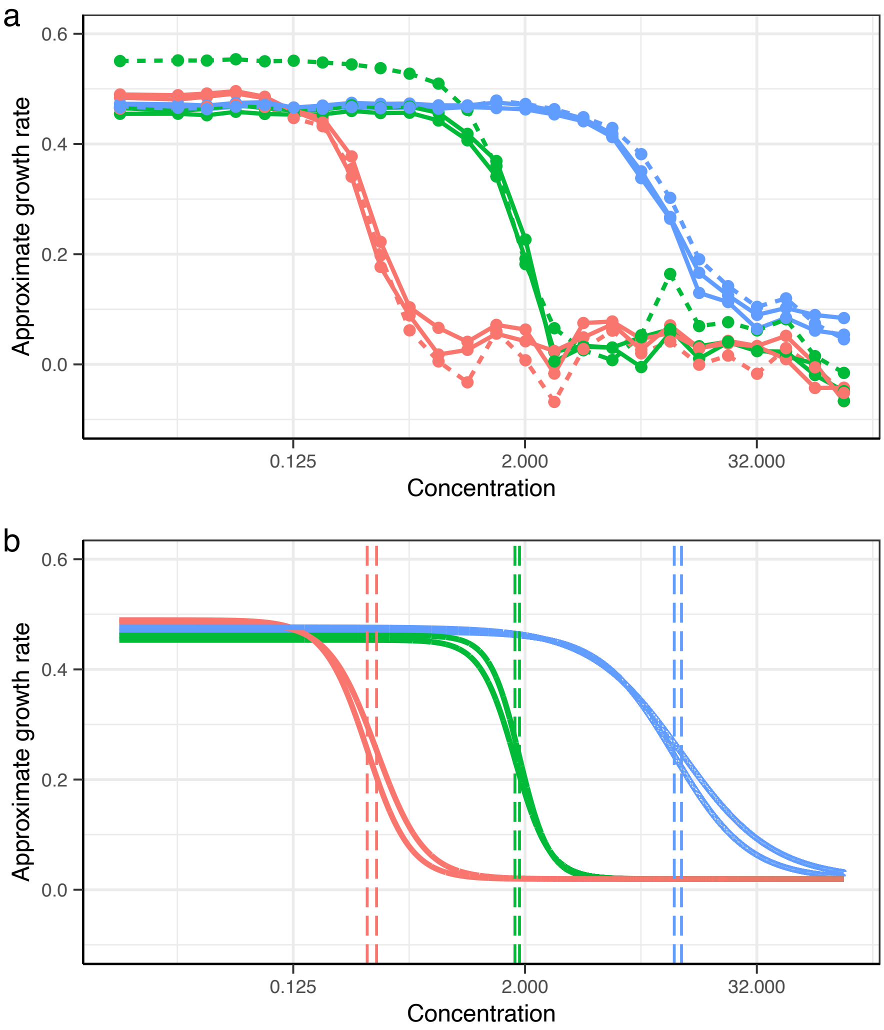

SI Figure 1: The approximate growth rates across the antibiotic gradient (a) yields a dose response curve by fitting a log-logistic (b). Three genotypes (E104K, G238S, and g4205a+M182T+G238S) are highlighted (pink, green and blue, respectively) from the *S. enterica* host. In part a, the three barcodes from each genotype are shown including the deviant barcode (dashed line). In part b, the level of resistance is given by the inflection point of the best-fit curve (dashed vertical line). The deviant barcode is removed.

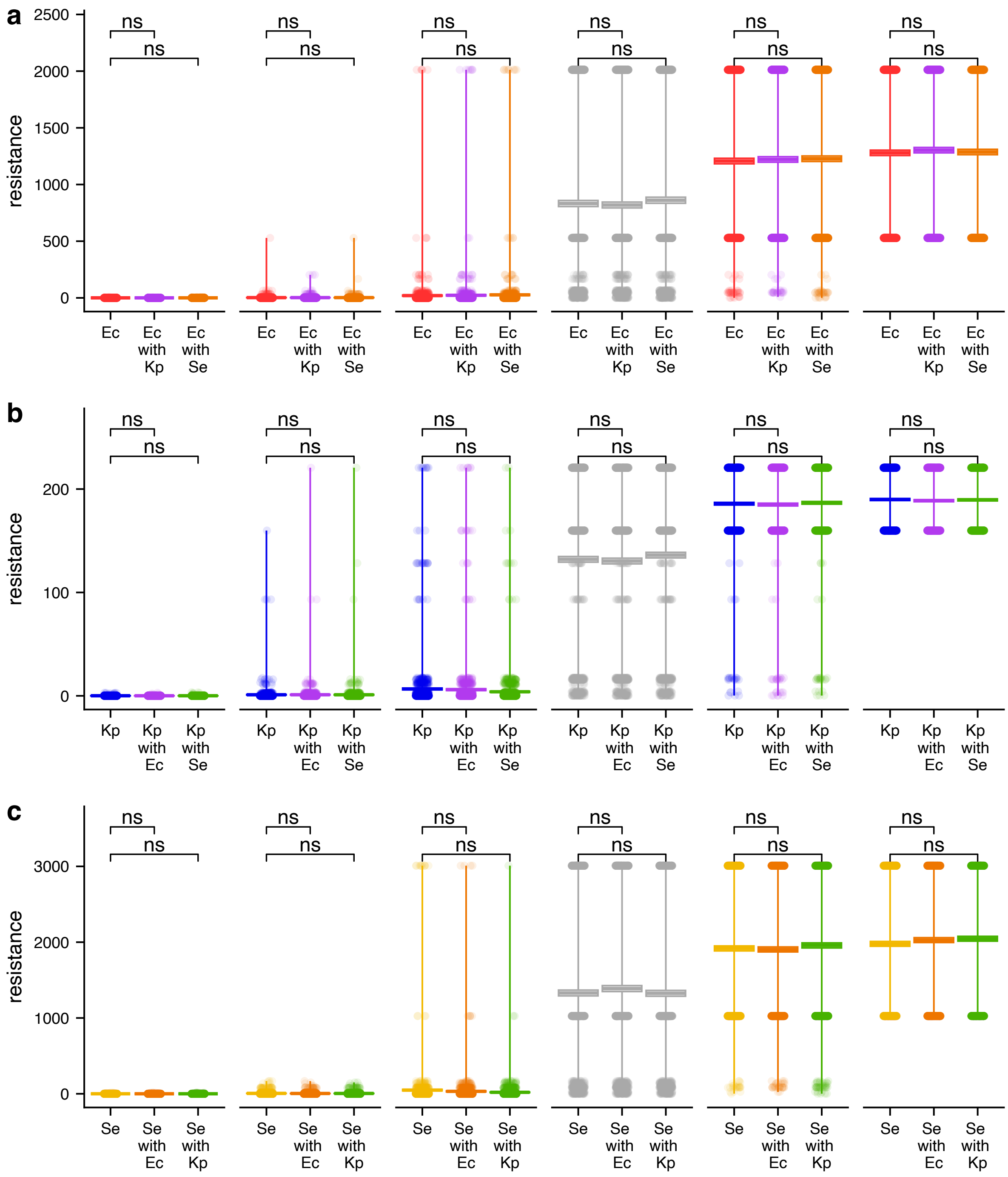

SI Figure 2: The effect of mutation rates on the evolutionary trends of the *bla*$_{TEM}$ gene. The graphical representation is the same as Figure 4d,h,l. The mutation rate increases from left to right (1 x 10^{-6}, 5 x 10^{-6}, 1 x 10^{-5}, 5 x 10^{-5}, 1 x 10^{-4}, and 5 x 10^{-4}). The gray triplicate in each part indicates the baseline simulation given in Figure 4.

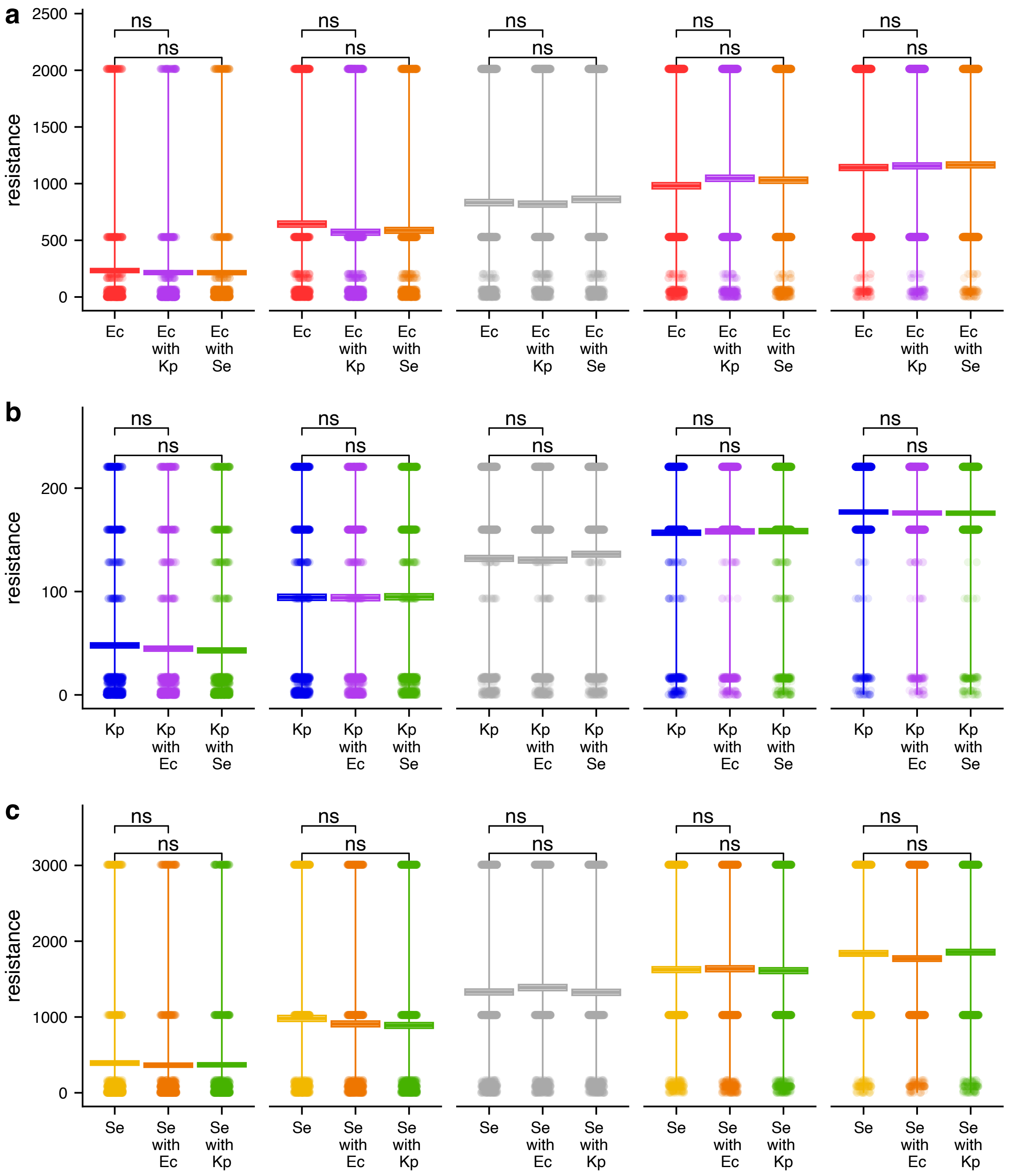

SI Figure 3: The effect of cumulative time on the evolutionary trends of the *bla*$_{TEM}$ gene. The graphical representation is the same as Figure 4d,h,l. The cumulative time increases from left to right (30, 45, 60, 75, and 90). The gray triplicate in each part indicates the baseline simulation given in Figure 4.

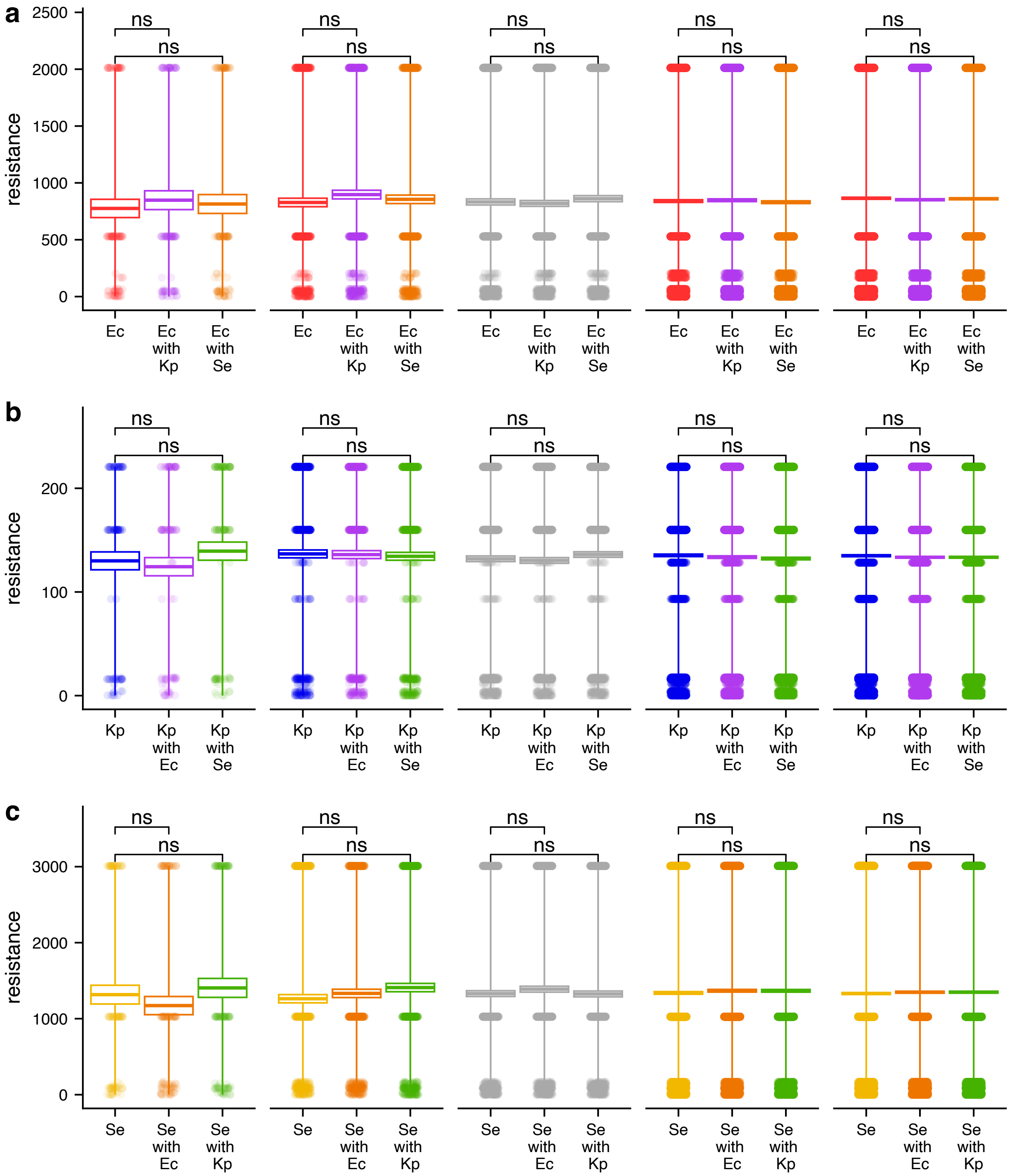

SI Figure 4: The effect of simulation replicates on the evolutionary trends of the *bla*$_{\text{TEM}}$ gene. The graphical representation is the same as Figure 4d,h,l. The number of replicates increases from left to right (100, 500, 1000, 5000, and 10000). The gray triplicate in each part indicates the baseline simulation given in Figure 4.

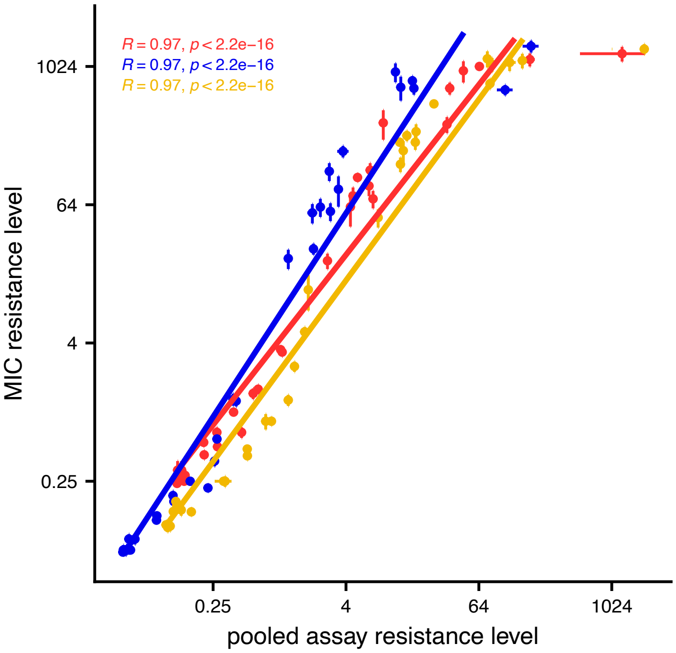

SI Figure 5: The pooled competition approach and a classic MIC assay produce highly correlated resistance measurements. Each genotype's resistance measure from the pooled competition approach is compared to the same genotype's resistance measure from a classic MIC assay. This comparison is done for each species: *E. coli* (red), *K. pneumoniae* (blue), and *S. enterica* (yellow). The correlation calculated using the Pearson correlation coefficient method was statistically significant for each species.

Supplementary Text Sections

Supplementary section 1: Interaction terminology

Here we discuss some terminology surrounding interaction effects and how this topic relates to our adaptive landscape framework. Most generally, we will be focusing on how the effect of a certain focal mutation on some response variable (e.g., drug resistance or competitive fitness) may change as some contextual variable changes. We take a broad view on the form of this contextual variable—it could be the state of other genetic loci in our focal organism, the genomic state of a host possessing our focal genetic element, the genotype of an organism that ecologically interacts with our focal organism, or even the state of the abiotic environment in which the focal organism finds itself. The classic example of "genetic epistasis" would be a case where the contextual variable is the allelic state at a genetic locus differing from our focal genetic locus (i.e., where the effect of the mutation at the focal locus depends on the allele at the second locus). However, we could imagine other forms of contextuality, which we will discuss below. First however, we will develop some terminology in which we can embed our discussion. These terms will also help us highlight some issues in special forms of interaction (e.g., sign epistasis).

Basic Terminology: For simplicity, let us imagine two factors that impact a phenotype of an organism, which we label $\mathbf{A}$ and $\mathbf{B}$. Note, the labels of our factors will generally be bolded, but the values of our factors will be unbolded and italicized. Here we consider that each factor has two values (a and A for factor $\mathbf{A}$, and b and B for factor $\mathbf{B}$). We assume that factor $\mathbf{A}$ gives the allelic state at some focal locus, such that a and A are alleles connected by mutation (and we will alternatively refer to $\mathbf{A}$ as a factor or a locus). However, factor $\mathbf{B}$ could be any number of things (allelic state of another locus, environmental state, etc.), although it may be easiest to start by thinking of it as a second locus. We can thus specify the values of all phenotypes (the response variable) that result from various combinations of our factors (the input variables); specifically, we have P_{ab}, P_{Ab}, P_{aB}, and P_{AB}. We will define the phenotypic effect of a mutation from $a \to A$ when factor $\mathbf{B}$ has value b as

$$\Delta^{(1)}_{(a\to A)|b} = P_{Ab} - P_{ab}$$

If the P values were fitness, this would be a (complicated) way of writing the selective coefficient of the mutation $a \to A$ given the value of b for the factor $\mathbf{B}$. Here, Δ is a difference in phenotypic values. The superscript on Δ denotes that this is a "first-order" difference (to be distinguished from second- and higher-order differences later). The subscript gives information on what change in the focal input variable is being considered (genotypes at our $\mathbf{A}$ locus) and the value of the contextual input variable. That is, "$(a \to A)|b$" can be read as the change $a \to A$ in factor $\mathbf{A}$ given a value of b in factor $\mathbf{B}$. Thus, if factor $\mathbf{B}$ is set to B, the equivalent difference is

$$\Delta^{(1)}_{(a\to A)|B} = P_{AB} - P_{aB}$$

We can now discuss a second-order difference; specifically, a difference of differences:

$$\Delta^{(2)}_{(a\to A)|(b\to B)} = \Delta^{(1)}_{(a\to A)|B} - \Delta^{(1)}_{(a\to A)|b} = (P_{AB} - P_{aB}) - (P_{Ab} - P_{ab})$$

This difference $\Delta^{(2)}_{(a\to A)|(b\to B)}$ quantifies how the phenotypic effect of the $a \to A$ mutation at locus $\mathbf{A}$ changes as factor $\mathbf{B}$ changes from b to B. This is a measure of an interaction effect (e.g., for G×G interactions, this is closely related to the second-order Walsh coefficient for a two-locus system; (Weinreich et al. 2013)). Indeed, when

$$\Delta^{(2)}_{(a\to A)|(b\to B)} = 0\,,$$

we must have

$$\Delta^{(1)}_{(a\to A)|B} = \Delta^{(1)}_{(a\to A)|b}\,,$$

which would mean that the effect of the $a \to A$ mutation would be independent of context (i.e., the value of factor $\mathbf{B}$). When the phenotypic effect of a change in factor $\mathbf{A}$ depends on the value of factor $\mathbf{B}$, we will say there is an $\mathbf{A}\times\mathbf{B}$ interaction (or in shorthand: a "$\mathbf{B}$-dependent impact of $\mathbf{A}$-change"), which requires

$$\Delta^{(2)}_{(a\to A)|(b\to B)} \neq 0$$

Even though we started by assuming that factor $\mathbf{A}$ was the focal variable and factor $\mathbf{B}$ was the contextual variable, we could switch their roles. Noting

$$\Delta^{(1)}_{(b\to B)|a} = P_{aB} - P_{ab}$$

$$\Delta^{(1)}_{(b\to B)|A} = P_{AB} - P_{Ab}$$

we have

$$\Delta^{(2)}_{(b\to B)|(a\to A)} = \Delta^{(1)}_{(b\to B)|A} - \Delta^{(1)}_{(b\to B)|a} = (P_{AB} - P_{Ab}) - (P_{aB} - P_{ab})$$

If

$$\Delta^{(2)}_{(b\to B)|(a\to A)} \neq 0$$

Then we could say that there is a $\mathbf{B}\times\mathbf{A}$ interaction. We note that we will generally label the focal factor first and the contextual factor second in our interaction terminology. However, because

$$(P_{AB} - P_{Ab}) - (P_{aB} - P_{ab}) = (P_{AB} - P_{aB}) - (P_{Ab} - P_{ab})$$

it follows that

$$\Delta^{(2)}_{(b\to B)|(a\to A)} = \Delta^{(2)}_{(a\to A)|(b\to B)}$$

This equation ensures that our measure of the $\mathbf{B}\times\mathbf{A}$ interaction (i.e., "$\mathbf{A}$-dependent impact of $\mathbf{B}$-change") is quantitatively equivalent to our measure of the the $\mathbf{A}\times\mathbf{B}$ interaction (i.e., "$\mathbf{B}$-dependent impact of $\mathbf{A}$-change"). We show this graphically using landscapes in Figure S1.1. Here the single measure of the interaction effect could be written as

$$\Delta^{(2)}_{\mathbf{AB}} = \Delta^{(2)}_{(a\to A)|(b\to B)} = \Delta^{(2)}_{(b\to B)|(a\to A)}$$

If factors $\mathbf{A}$ and $\mathbf{B}$ were allelic states at different loci, then $\Delta^{(2)}_{\mathbf{AB}}$ would quantify genetic epistasis (i.e., our $\mathbf{A}\times\mathbf{B}$ interaction would be a G×G interaction).

We can use this framework now to discuss a case of "sign interaction" (e.g., sign epistasis). We will say that there is a sign $\mathbf{A}\times\mathbf{B}$ interaction (or "sign $\mathbf{B}$-dependent impact of $\mathbf{A}$-change") when the following condition holds

$$\max\left[\Delta^{(1)}_{(a\to A)|B}, \Delta^{(1)}_{(a\to A)|b}\right] > 0 > \min\left[\Delta^{(1)}_{(a\to A)|B}, \Delta^{(1)}_{(a\to A)|b}\right] \qquad \text{[S1]}$$

If factors $\mathbf{A}$ and $\mathbf{B}$ were allelic states at different loci, this would be a case of sign genetic epistasis, where the effect of a mutation at a focal locus changes in sign as the allele at another (contextual) locus changes. Note that condition [S1] constitutes a strict definition for sign interaction. We could consider a definition that also included borderline cases where the effect of the $a \to A$ change was neutral in one context and non-neutral in another, which could be written as follows:

$$\Delta^{(2)}_{(a\to A)|(b\to B)} \neq 0 \qquad [S2a]$$

$$\max\left[\Delta^{(1)}_{(a\to A)|B}, \Delta^{(1)}_{(a\to A)|b}\right] \geq 0 \geq \min\left[\Delta^{(1)}_{(a\to A)|B}, \Delta^{(1)}_{(a\to A)|b}\right] \qquad [S2b]$$

Given that we found quantitative symmetry for our interaction coefficient ($\Delta^{(2)}_{(a\to A)|(b\to B)} = \Delta^{(2)}_{(b\to B)|(a\to A)}$) do we also have symmetry for a sign interaction effect? That is, does the existence of "sign $\mathbf{B}$-dependent impact of $\mathbf{A}$-change" imply the existence of "sign $\mathbf{A}$-dependent impact of $\mathbf{B}$-change?" The answer is no, and Figure S1.1 gives an example of such asymmetry. Specifically, there is a sign $\mathbf{B}\times\mathbf{A}$ interaction—the effects of the $b \to B$ mutation at locus $\mathbf{B}$ change in sign as locus $\mathbf{A}$ changes from a to A (the first two arrows are pointing in opposite directions in Figure S1.1b). However, there is not a sign $\mathbf{A}\times\mathbf{B}$ interaction, as the effects of the $a \to A$ mutation at locus $\mathbf{A}$ are of the same sign (the first two arrows both point upward in Figure S1.1a). For the case of two genetic loci, if there is sign epistasis when each factor plays the focal role, then this is called *reciprocal* sign epistasis (which occurs if there is both a sign $\mathbf{A}\times\mathbf{B}$ interaction *and* a sign $\mathbf{B}\times\mathbf{A}$ interaction; see Figure S1.2 for an example). Given that the existence of sign epistasis does not guarantee reciprocal sign epistasis, we see that it now becomes important to refer to which factor is playing the focal role and which factor is playing the contextual role. Thus, our terminology of placing the focal factor first in an interaction (e.g., $\mathbf{A}\times\mathbf{B}$ for a focal $\mathbf{A}$ factor versus $\mathbf{B}\times\mathbf{A}$ for a focal $\mathbf{B}$ factor) has an advantage for describing sign interaction more accurately.

This distinction may have been less important in previous discussions when

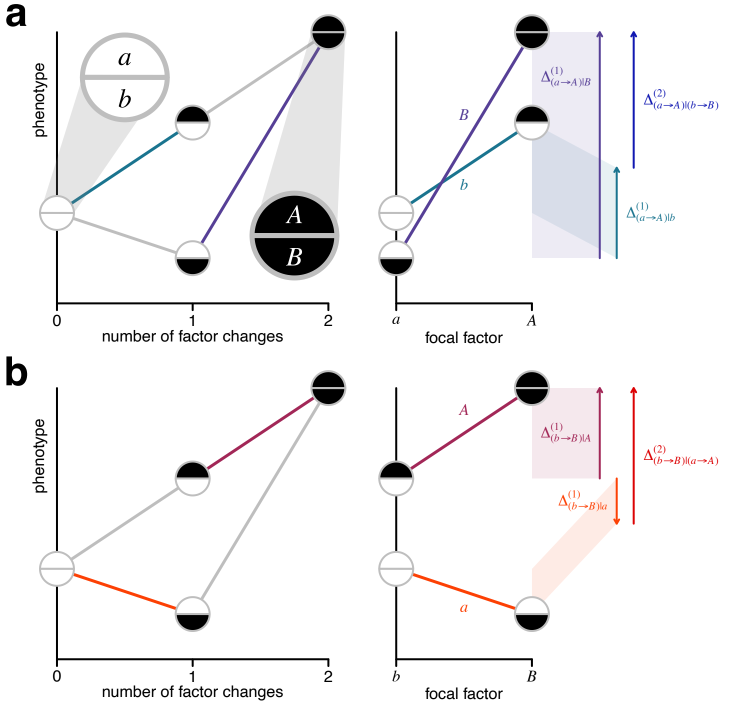

Figure S1.1: Here a landscape for two factors ($\mathbf{A}$ and $\mathbf{B}$) is shown. The state of each factor is shown by the plotted pie chart, where the state of the $\mathbf{A}$ factor is the top half and the state of the $\mathbf{B}$ factor is the bottom half. The "reference" values of each factor are given by lowercase letters (a and b) and white pie slices, while changes from this reference are indicated by uppercase letters (A and B) and black pie slices (see the blowup pies). (**a**) The full landscape where the change $a \to A$ is highlighted in the b (teal edge) and B (violet edge) backgrounds. In the graph to the right, the change in the focal factor is emphasized (by placing the $\mathbf{A}$ factor states on the x-axis) and each segment corresponds to a different state of the contextual factor (here $\mathbf{B}$). The (first-order) phenotype change due to the $a \to A$ change is given by the leftmost arrows. We note that we have moved one of these arrows (representing $\Delta^{(1)}_{(a\to A)|b}$) so that it starts at the same vertical value as the other arrow (i.e., $\Delta^{(1)}_{(a\to A)|B}$). Because $\Delta^{(1)}_{(a\to A)|B} = \Delta^{(2)}_{(a\to A)|(b\to B)} + \Delta^{(1)}_{(a\to A)|b}$, after our arrow reposition, all we need to do to get the second order difference ($\Delta^{(2)}_{(a\to A)|(b\to B)}$, or a difference of the differences) is to go from vertical value of the head of the $\Delta^{(1)}_{(a\to A)|b}$ arrow to value of the head of the $\Delta^{(1)}_{(a\to A)|B}$ arrow. (**b**) Here we consider the same landscape but the change $b \to B$ is highlighted in the a (light red edge) and A (purple edge) backgrounds. It is apparent that the second-order change with a focal $\mathbf{B}$ factor (the red arrow giving $\Delta^{(2)}_{(b\to B)|(a\to A)}$) is identical (in sign and magnitude) to the second-order change with a focal $\mathbf{A}$ factor (the blue arrow giving $\Delta^{(2)}_{(a\to A)|(b\to B)}$). That is, there is a single value ($\Delta^{(2)}_{\mathbf{AB}}$) measuring the interaction between these factors.

both factors are of the same category. For instance, if both factor $\mathbf{A}$ and factor $\mathbf{B}$ are allelic states at genetic loci, then sign $\mathbf{A}\times\mathbf{B}$ interaction and sign $\mathbf{B}\times\mathbf{A}$ interaction could both be described as sign genetic epistasis. Here we take "genetic" to be a description of the category of the contextual factor—the sign of the phenotypic effect of a mutation at one locus depends on the allelic state at another (genetic) locus.

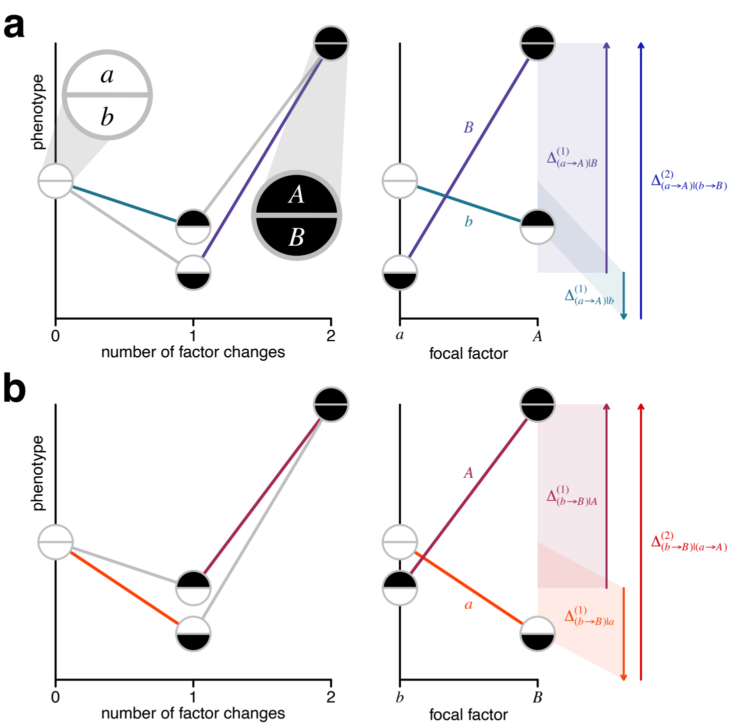

Figure S2: A landscape with a reciprocal sign interaction. This figure follows the exact conventions of Figure S1. Here, however, the leftmost two arrows in part **a** and part **b** are pointing in opposite directions. Thus, there is both a sign $\mathbf{A}\times\mathbf{B}$ and a sign $\mathbf{B}\times\mathbf{A}$ interaction—or a case of reciprocal sign interaction.

However, the distinction starts to get more relevant when the factors are of different categories. For instance, suppose that factor $\mathbf{B}$ gives the state of the abiotic environment (with possible values b and B). Furthermore, suppose that the phenotype of the a genotype decreases when moving from environment b to B, whereas the phenotype of the A genotype does the opposite. This is precisely the situation illustrated in Figure S1.1b, where the two segments in the right graph could be considered reaction norms. While such a graph would traditionally be called a case of (sign) G×E, here we would actually phrase this as a sign E×G interaction, and we might note that there was not a *sign* G×E interaction in this case (Figure S1.1a; note this is a case of *magnitude* G×E). This distinction has relevance for our paper because when we refer to a sign G×H interaction, we are explicitly considering the genotype at the plasmid locus to be the focal factor and the host genome to be the contextual factor. More specifically, a sign G×H interaction means that the effect on resistance of a mutation in the plasmid-borne *bla*$_{\mathrm{TEM}}$ gene changes in sign as the host genome changes. This is distinct from a claim of a sign H×G interaction, which means that the effect on resistance of a shift in host changes in sign as the *bla*$_{\mathrm{TEM}}$ genotype changes. In cases where there are both sign G×H and sign H×G effects, we could refer to this as reciprocal sign interaction between the plasmid gene and host genome.

Higher-order interactions: In the same way that we introduced second-order differences (i.e., differences of differences), there are also third-order and even higher-order differences. These higher-order differences quantify higher-order interactions. To illustrate, here we will focus on third-order interaction. Let us imagine three factors, $\mathbf{A}$, $\mathbf{B}$, and $\mathbf{C}$, where each factor has two values (a and A for factor $\mathbf{A}$, b and B for factor $\mathbf{B}$, and c and C for factor $\mathbf{C}$). Suppose we fix factor $\mathbf{C}$ to the value c, then the second-order difference for the remaining factors can be computed in the normal way:

$$\Delta^{(2)}_{\mathbf{AB}|c} = \Delta^{(1)}_{(a\to A)|\{B,c\}} - \Delta^{(1)}_{(a\to A)|\{b,c\}}$$

Here we note that we also have to add the value c for factor $\mathbf{C}$ as part of the context (both for the first and second-order differences). Of course, we could also compute the same second-order difference where factor $\mathbf{C}$ changes to a value of C:

$$\Delta^{(2)}_{\mathbf{AB}|C} = \Delta^{(1)}_{(a\to A)|\{B,C\}} - \Delta^{(1)}_{(a\to A)|\{b,C\}}$$

Now we can consider an even higher-order (a third-order) difference:

$$\Delta^{(3)}_{\mathbf{ABC}} = \Delta^{(2)}_{\mathbf{AB}|C} - \Delta^{(2)}_{\mathbf{AB}|c}$$
$$= \Delta^{(2)}_{[(a\to A)|(b\to B)]|C} - \Delta^{(2)}_{[(a\to A)|(b\to B)]|c}$$

Here again we are using shorthand $\Delta^{(3)}_{\mathbf{ABC}}$ and we note that also:

$$\Delta^{(3)}_{\mathbf{ABC}} = \Delta^{(2)}_{\mathbf{AC}|B} - \Delta^{(2)}_{\mathbf{AC}|b} = \Delta^{(2)}_{\mathbf{BC}|A} - \Delta^{(2)}_{\mathbf{BC}|a}$$

We show that the value of the third-order difference does not depend on the contextual factor in Figure S1.3. Another way of writing this third-order difference is

$$\Delta^{(3)}_{\mathbf{ABC}} = \Delta^{(2)}_{[(a\to A)|(b\to B)]|(c\to C)}$$

In Table S1, we list all differences relevant for this three-factor system. There is an $\mathbf{A}\times\mathbf{B}\times\mathbf{C}$ interaction when:

$$\Delta^{(3)}_{\mathbf{ABC}} \neq 0$$

In a way analogous to our discussion above, we can also define higher-order sign interaction too. We will say that there is sign $(\mathbf{A}\times\mathbf{B})\times\mathbf{C}$ interaction when

Table S1.1: Differences with Three Factors

Difference	Shorthand	Lower-order Definition	Equivalent to:
$\Delta^{(1)}_{(a\to A)\mid\{b,c\}}$	$\Delta^{(1)}_{\mathbf{A}\mid\{b,c\}}$	$P_{Abc} - P_{abc}$	
$\Delta^{(1)}_{(a\to A)\mid\{B,c\}}$	$\Delta^{(1)}_{\mathbf{A}\mid\{B,c\}}$	$P_{ABc} - P_{aBc}$	
$\Delta^{(1)}_{(a\to A)\mid\{b,C\}}$	$\Delta^{(1)}_{\mathbf{A}\mid\{b,C\}}$	$P_{AbC} - P_{abC}$	
$\Delta^{(1)}_{(a\to A)\mid\{B,C\}}$	$\Delta^{(1)}_{\mathbf{A}\mid\{B,C\}}$	$P_{ABC} - P_{aBC}$	
$\Delta^{(1)}_{(b\to B)\mid\{a,c\}}$	$\Delta^{(1)}_{\mathbf{B}\mid\{a,c\}}$	$P_{aBc} - P_{abc}$	
$\Delta^{(1)}_{(b\to B)\mid\{A,c\}}$	$\Delta^{(1)}_{\mathbf{B}\mid\{A,c\}}$	$P_{ABc} - P_{Abc}$	
$\Delta^{(1)}_{(b\to B)\mid\{a,C\}}$	$\Delta^{(1)}_{\mathbf{B}\mid\{a,C\}}$	$P_{aBC} - P_{abC}$	
$\Delta^{(1)}_{(b\to B)\mid\{A,C\}}$	$\Delta^{(1)}_{\mathbf{B}\mid\{A,C\}}$	$P_{ABC} - P_{AbC}$	
$\Delta^{(1)}_{(c\to C)\mid\{a,b\}}$	$\Delta^{(1)}_{\mathbf{C}\mid\{a,b\}}$	$P_{abC} - P_{abc}$	
$\Delta^{(1)}_{(c\to C)\mid\{A,b\}}$	$\Delta^{(1)}_{\mathbf{C}\mid\{A,b\}}$	$P_{AbC} - P_{Abc}$	
$\Delta^{(1)}_{(c\to C)\mid\{a,B\}}$	$\Delta^{(1)}_{\mathbf{C}\mid\{a,B\}}$	$P_{aBC} - P_{aBc}$	
$\Delta^{(1)}_{(c\to C)\mid\{A,B\}}$	$\Delta^{(1)}_{\mathbf{C}\mid\{A,B\}}$	$P_{ABC} - P_{ABc}$	
$\Delta^{(2)}_{[(a\to A)\mid(b\to B)]\mid c}$	$\Delta^{(2)}_{\mathbf{AB}\mid c}$	$\Delta^{(1)}_{\mathbf{A}\mid\{B,c\}} - \Delta^{(1)}_{\mathbf{A}\mid\{b,c\}}$	$\Delta^{(2)}_{[(b\to B)\mid(a\to A)]\mid c}$
$\Delta^{(2)}_{[(a\to A)\mid(b\to B)]\mid C}$	$\Delta^{(2)}_{\mathbf{AB}\mid C}$	$\Delta^{(1)}_{\mathbf{A}\mid\{B,C\}} - \Delta^{(1)}_{\mathbf{A}\mid\{b,C\}}$	$\Delta^{(2)}_{[(b\to B)\mid(a\to A)]\mid c}$
$\Delta^{(2)}_{[(a\to A)\mid(c\to C)]\mid b}$	$\Delta^{(2)}_{\mathbf{AC}\mid b}$	$\Delta^{(1)}_{\mathbf{A}\mid\{b,C\}} - \Delta^{(1)}_{\mathbf{A}\mid\{b,c\}}$	$\Delta^{(2)}_{[(c\to C)\mid(a\to A)]\mid b}$
$\Delta^{(2)}_{[(a\to A)\mid(c\to C)]\mid B}$	$\Delta^{(2)}_{\mathbf{AC}\mid B}$	$\Delta^{(1)}_{\mathbf{A}\mid\{B,C\}} - \Delta^{(1)}_{\mathbf{A}\mid\{B,c\}}$	$\Delta^{(2)}_{[(c\to C)\mid(a\to A)]\mid B}$
$\Delta^{(2)}_{[(b\to B)\mid(c\to C)]\mid a}$	$\Delta^{(2)}_{\mathbf{BC}\mid a}$	$\Delta^{(1)}_{\mathbf{B}\mid\{a,C\}} - \Delta^{(1)}_{\mathbf{B}\mid\{a,c\}}$	$\Delta^{(2)}_{[(c\to C)\mid(b\to B)]\mid a}$
$\Delta^{(2)}_{[(b\to B)\mid(c\to C)]\mid A}$	$\Delta^{(2)}_{\mathbf{BC}\mid A}$	$\Delta^{(1)}_{\mathbf{B}\mid\{A,C\}} - \Delta^{(1)}_{\mathbf{B}\mid\{A,c\}}$	$\Delta^{(2)}_{[(c\to C)\mid(b\to B)]\mid A}$
$\Delta^{(3)}_{[(a\to A)\mid(b\to B)]\mid(c\to C)}$	$\Delta^{(3)}_{\mathbf{ABC}}$	$\Delta^{(2)}_{\mathbf{AB}\mid C} - \Delta^{(2)}_{\mathbf{AB}\mid c}$	$\Delta^{(3)}_{[(b\to B)\mid(a\to A)]\mid(c\to C)}$, $\Delta^{(3)}_{[(a\to A)\mid(c\to C)]\mid(b\to B)}$, $\Delta^{(3)}_{[(c\to C)\mid(a\to A)]\mid(b\to B)}$, $\Delta^{(3)}_{[(b\to B)\mid(c\to C)]\mid(a\to A)}$, $\Delta^{(3)}_{[(c\to C)\mid(b\to B)]\mid(a\to A)}$

$$\max\left[\Delta^{(2)}_{[(a\to A)|(b\to B)]|C}, \Delta^{(2)}_{[(a\to A)|(b\to B)]|c}\right] > 0 > \min\left[\Delta^{(2)}_{[(a\to A)|(b\to B)]|C}, \Delta^{(2)}_{[(a\to A)|(b\to B)]|c}\right]$$

This higher-order sign interaction is a statement about how lower-order interaction changes sign. Again, it is critical to make clear the factor that is contextual (here $\mathbf{C}$) and the factors for which the lower-order interaction is being assessed (here $\mathbf{A}$ and $\mathbf{B}$). We could call these the focal factors. We write the sign interaction as "sign $(\mathbf{A}\times\mathbf{B})\times\mathbf{C}$" in order to clearly group the focal factors (listed first in parentheses) and separate them from the contextual factor (listed last). While a sign $(\mathbf{A}\times\mathbf{B})\times\mathbf{C}$ interaction does imply a sign $(\mathbf{B}\times\mathbf{A})\times\mathbf{C}$ interaction, it does not imply a sign $(\mathbf{A}\times\mathbf{C})\times\mathbf{B}$ interaction. That is, the "sign" designation depends on the factor that plays the contextual role. We illustrate this asymmetry in Figure S1.3. Here there is a sign $(\mathbf{A}\times\mathbf{C})\times\mathbf{B}$ interaction, but not a sign $(\mathbf{A}\times\mathbf{B})\times\mathbf{C}$ interaction. It may be the case that all possible higher order interactions (e.g., the three third-order interactions where each factor plays the role of context) are sign interactions. This would be the equivalent of reciprocal sign epistasis in the case of two genetic factors. However, there are more than two orderings of higher-order interaction where the identity of the contextual factor now matters. Thus, we suggest replacing the term "reciprocal" with the term "complete." In Figure S1.4, we show a landscape with complete sign $\mathbf{A}\times\mathbf{B}\times\mathbf{C}$ interaction (note we can drop the parentheses, because the sign interaction applies to every factor in the contextual role). Finally, we will reserve the term "universal" if all possible interactions *of every order* are sign interactions (the landscape in Figure S1.4 also exhibits universal sign interaction).

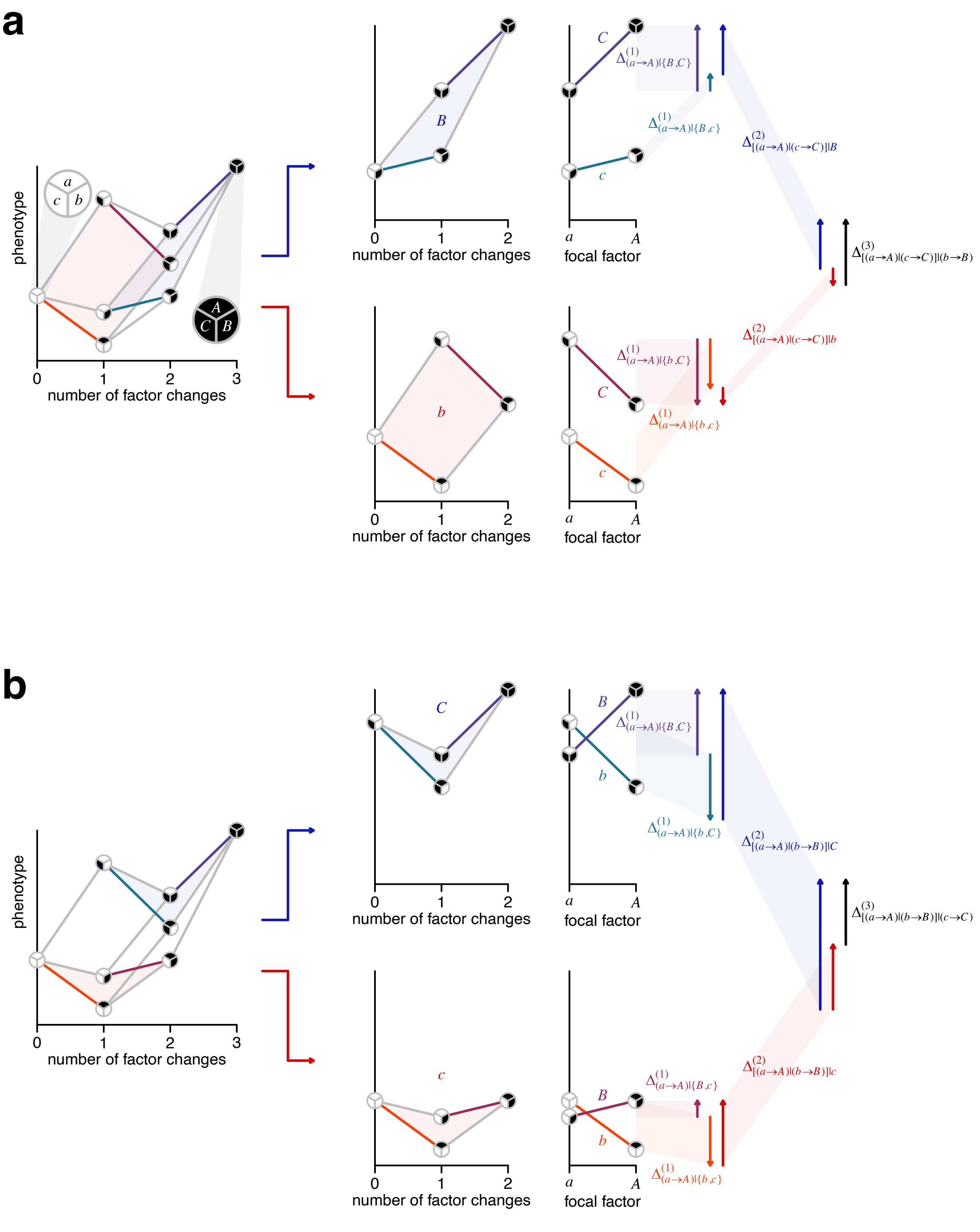

Figure S1.3: A case of higher-order sign interaction that is not "complete." (**a**) A three-factor landscape is shown. First the landscape is broken down into 2 two-factor landscapes by fixing the $\mathbf{B}$ factor at the value B (top graph) and b (bottom graph). Then each of these sub-landscapes are further broken down by $\mathbf{C}$ factor values, such that the effect of the focal factor (here $\mathbf{A}$) is emphasized. The first two of the upper triplet of arrows give the first-order differences with the $\mathbf{B}$ factor fixed at B (the effect of the $a \to A$ change in the backgrounds of $\{B, C\}$ and $\{B, c\}$) while the first two of the lower triplet of arrows give the first-order differences with the $\mathbf{B}$ factor fixed at b (the effect of the $a \to A$ change in the backgrounds of $\{b, C\}$ and $\{b, c\}$). The third arrow in each of these triplets give the second-order differences: the effect of the $a \to A$ change as factor $\mathbf{C}$ changes from c to C with factor $\mathbf{B}$ fixed at B (upper) or b (lower). Each second-order difference is shifted so their arrows start at the same point, which allows the third-order difference to be computed (black arrow). This is a case of $(\mathbf{A} \times \mathbf{C}) \times \mathbf{B}$ sign interaction (because the blue and red arrows point in different directions). (**b**) The same landscape is shown, but here the $\mathbf{C}$ factor is the contextual factor. We see that the length and direction of the third-order difference (black arrow) is the same as in part (a), but this $(\mathbf{A} \times \mathbf{B}) \times \mathbf{C}$ interaction is not a third-order sign interaction (as the blue and red arrows point in the same direction).

In our system, because we are considering multiple genetic sites in our plasmid gene, we can certainly explore G×G×H interaction (or even higher-order interactions). Further we can explore whether there is sign (G×G)×H, which could be called sign host-dependent genetic epistasis. We note this is not the same thing as sign (G×H)×G. In Figure S1.3, we see this potential asymmetry. The utility of these higher-order interactions and their magnitude and sign characterization is a topic that is ripe for future study.

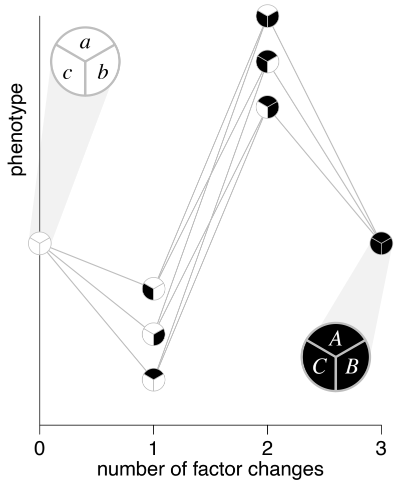

Figure S1.4: A landscape with universal sign interaction. At the second order, this landscape has complete sign $\mathbf{A} \times \mathbf{B}$ interaction (i.e., sign $\mathbf{A} \times \mathbf{B}$ interaction *and* sign $\mathbf{B} \times \mathbf{A}$ interaction, also termed a reciprocal sign interaction), complete sign $\mathbf{A} \times \mathbf{C}$ interaction, and complete sign $\mathbf{B} \times \mathbf{C}$ interaction. At the third order, this landscape has complete sign $\mathbf{A} \times \mathbf{B} \times \mathbf{C}$ interaction (i.e., sign $(\mathbf{A} \times \mathbf{B}) \times \mathbf{C}$ interaction *and* sign $(\mathbf{A} \times \mathbf{C}) \times \mathbf{B}$ interaction *and* sign $(\mathbf{B} \times \mathbf{C}) \times \mathbf{A}$ interaction).

Supplementary section 2: Landscape representations

In our simulations, we are taking the host species to be analogous to an environmental factor. That is, the evolving mobile gene experiences different host environments as it moves between species. For the n possible mutations in the mobile gene, we represented this as a distinct 2^n node landscape for each distinct host. In our simulations, during periods where the gene resided in a single host, evolution by mutation and selection involved an upward trajectory within the relevant species' landscape. When the mobile gene moved to a new host, the landscape "shifted" to the topography corresponding to the new host species, where an upward trajectory ensued. This kind of dynamic landscape has been termed a "seascape" (Mustonen and Lässig 2009). A sign G×H interaction occurs if the slope between a pair of mutationally connected genotypes changes direction as mobile gene moves from one host to another. However, because the host genome (the "H" factor) is itself another genetic component in the system, it also seems reasonable to think of this as another "genetic locus" (making it a "G" factor). In this light, G×H interactions would be standard genetic epistasis. Perhaps more interestingly, instead of considering a distinct landscape for each host, we could expand the landscape to a higher dimension. For instance, suppose we are considering two host species. Treating host as a biallelic locus, we could go from a pair of 2^n node landscapes to a single 2^{n+1} landscape. Or put another way, we could shift from a lower-dimensional dynamic seascape to a higher-dimensional fixed landscape. We do not make this higher dimensional move in our system for a few reasons. In order to understand these, it will help to first take a detour into a case of intergenomic epistasis.

Here we consider a system of two interacting species (e.g., a host and a pathogen). To keep things simple, we will assume that each species has a single biallelic locus: species $\mathbf{A}$ has genotypes a and A, and species $\mathbf{B}$ has genotypes b and B. Here we will assume that selection is strong relative to mutation such that the population for each species can be well described be a single genotype at nearly all points in time. Here we consider an interesting proposal made by Bank (2022) involving combining the genotypes of each species into a "meta-genotype." Denoting species $\mathbf{A}$ in red and species $\mathbf{B}$ in blue, these four meta-genotypes would be ab, Ab, aB, and AB. Coevolution of this community could then be envisioned as movement in the space of these four meta-genotypes. While each meta-genotype could be mapped to some community-level phenotype (e.g., some community function in the context of directed community-level selection (see Xie et al. 2019; Sánchez et al. 2021; Sanchez et al. 2023)), here instead we consider *two* landscapes for our set of meta-genotypes. Specifically, each landscape gives the fitness value of the genotype in one species (given the context of the other species' genotype). If we think of the genotypes as strategies in a game between different "species players," then these plots are just the visualization for both components of the 2×2 asymmetric game payoff matrix. We emphasize that these fitnesses can be dependent on the genotypic context of the other species. In Figure S2.1, we consider an interesting case where the meta-genotypic landscapes exhibit reciprocal intergenomic sign epistasis.

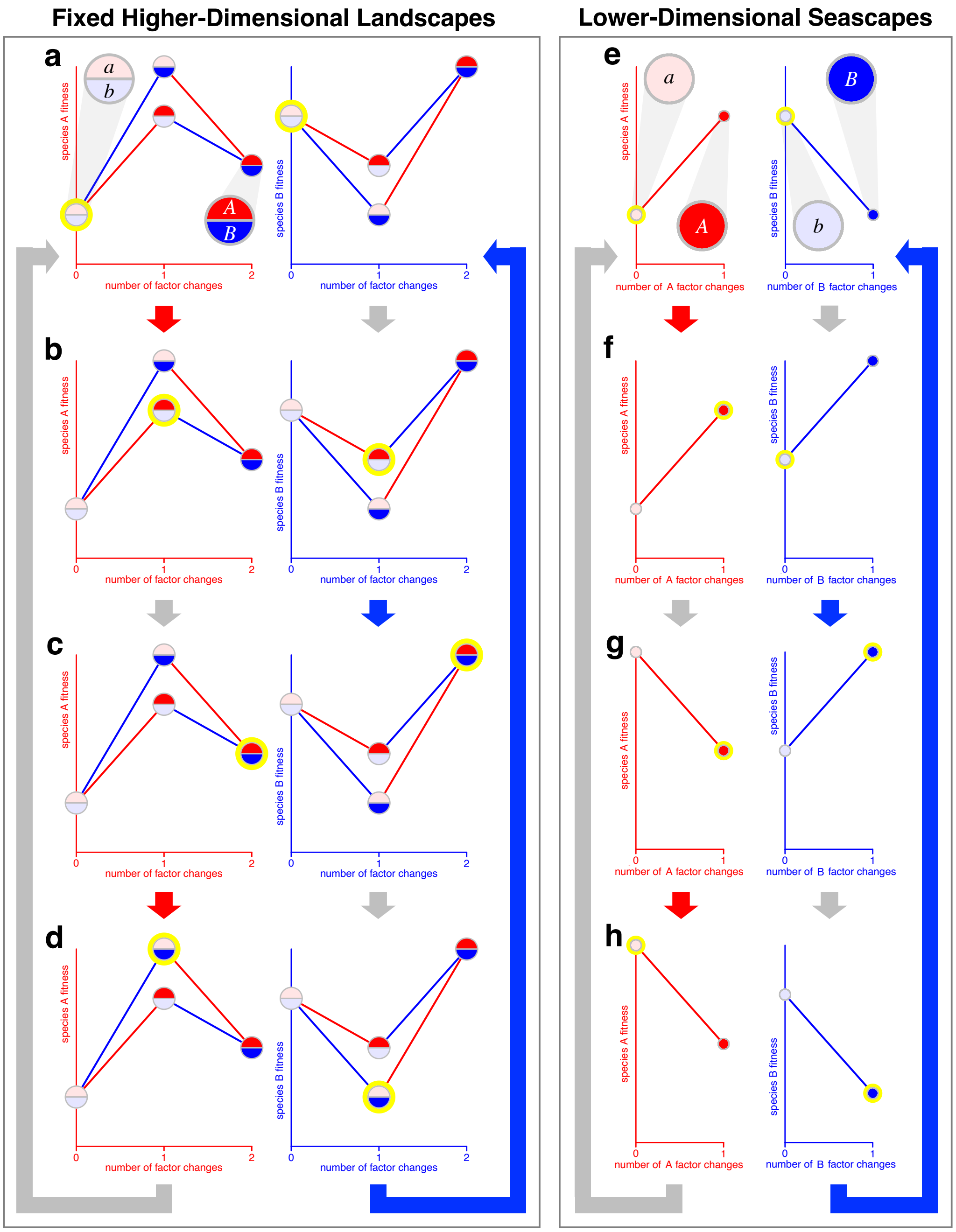

Figure S2.1: Fixed higher-dimensional meta-genotype landscapes versus dynamic lower-dimensional genotypic seascapes. (**a**) Each meta-genotype landscape is defined over the same meta-genotypic space (*ab*, *Ab*, *aB*, and *AB*, where species **A** genotype is in red and the species **B** genotype is in blue). The left landscape (red axes) maps these meta-genotypes to the fitness of the species **A** genotype (in the context of the relevant species **B** genotype), while the right landscape (blue axes) gives similar fitnesses for the species **B** genotypes. The community starts with the *a* genotype for species **A** and the *b* genotype for species **B** (highlighted meta-genotype *ab*). Genotype *A* is selected in the background of genotype *b* so the community will shift its meta-genotype. This occurs due to selection in species **A** (red block arrow) and due to a biotic context shift for species **B** (gray arrow). (**b**) Now positioned at meta-genotype *Ab* (highlighted node), genotype *B* is selected in the background of genotype *A* so the community will shift its meta-genotype again via selection in species **B** (blue arrow) and a biotic context shift for species **A** (gray arrow). (**c**) Now at *AB*, the community shifts to *aB* (**d**) and then back to *ab* (part **a**) to create a meta-genotypic loop, where arrows in color indicate selection and arrows in gray indicate biotic context shifts. (**e-h**) Here the landscapes for each species are laid out in the more traditional way for a single biallelic locus in species **A** (left graph) and a single biallelic locus for species **B** (right graph). The same evolutionary sequence is illustrated, evolution by selection is indicated by an arrow in color, whereas a landscape change due to a partner species evolving is indicated by a gray arrow (note that the genotypic position of the species in this latter case stays constant). The meta-genotypic landscapes in parts (**a-d**) remain fixed, whereas the genotypic landscapes in parts (**e-h**) are dynamic (i.e., seascapes).

The community starts on the meta-genotype ab (highlighted node in Figure S2.1a). Because genotype A is more fit than a in species **A** when genotype b is fixed in species **B**, the community shifts to meta-genotype Ab (Figure S2.1b). We note that this node movement occurs in both the **A** and **B** landscapes, which yields a lift in fitness in species **A** (due to selection) and a drop in the fitness of species **B** (due to a change in biotic context). Because genotype B is more fit than genotype b in species **B** in the presence of genotype A in species **A**, the community now shifts from Ab to AB (Figure S2.1c), which leads to an increase in fitness in species **B** (due to selection) and a decrease in fitness in species **A** (due to a change in context). However, now the direction of selection has changed for species **A** and the community moves from AB to aB (Figure S2.1d). And finally, because the direction of selection has flipped for species **B**, the community moves from aB back to its starting meta-genotype of ab (Figure S2.1a). In this case, the meta-genotypic landscapes are fixed. However, we also start to see how a basic intuition about adaptive walks on landscapes gets violated. Specifically, an adaptive walk can lead to *downhill* movement. If we think of adaptive walks as strictly climbing trajectories, this appears confusing at first glance. Of course, it occurs in a focal species' landscape when the other species is adapting and the altered genotypic context lowers the fitness of the focal resident's genotype. This phenomenon can lead to interesting outcomes such as an adaptive evolutionary trajectory that ends up where it started. This is not behavior familiar to our experience with landscapes: it is exceedingly difficult to climb continuously uphill on a static landscape and end up where you started! However, it is as if our species occupy landscapes similar to an M.C. Escher optical illusion, where such circuits can occur. If one is not bothered by this capricious behavior regarding climbing, then the meta-genomic extension may be appealing (as the landscape for each species is a fixed object; see (Tanaka et al. 2020) for a related discussion). However, there is another option as well. In Figures S2.1e-h, we replay the evolutionary sequence using a pair of interacting seascapes. Here each species occupies a (dynamic) lower-dimensional seascape. Movement within a seascape always proceeds uphill, but the shape of the seascape changes as the other species evolves. While this representation of coevolution has the added complexity of landscape change (i.e., the seascape), it has the advantage that the behavior within each seascape conforms to standard expectations. It is for a related reason that we find the lower-dimensional representation more intuitive in our system, to which we now turn.

As mentioned above, we treat host similar to an environmental factor, and handle the plasmid-gene landscape as a kind of seascape. However, it certainly is possible to treat the host as an additional locus (as in Figure S2.2a). Despite some advantages (e.g., a fixed compact landscape form), we resist this representation for a few reasons. First, in our simulations, the host only switches rarely. Thus, the plasmid gene is evolving on one host landscape for some time before switching to the other. It is as if evolution is constrained to host-specific subsets of the higher-dimensional landscape (e.g., Figures S2.2b and S2.2c)

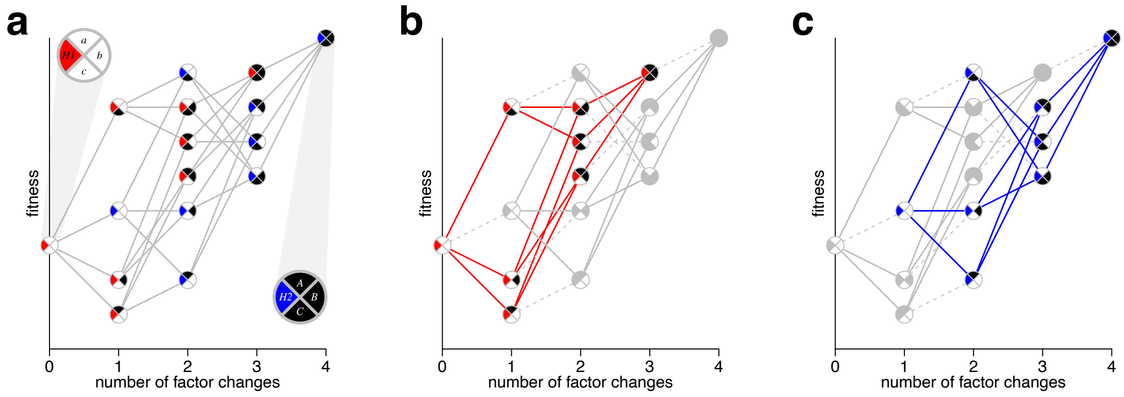

Figure S2.2: A landscape for three biallelic sites (a/A, b/B, and c/C) within a plasmid gene is shown, where the host identity (H1 or H2) is encoded as an "additional locus." (**a**) To distinguish the "alleles" at this locus, we represent the state of host 1 with a red pie slice and the state of host 2 with a blue pie slice. (**b**) Here we focus on the sub-landscape involving host 1. All edges that connect single mutations in the plasmid gene that remain in host 1 are indicated in red. The dashed edges involve a host shift of the same plasmid genotype. The sub-landscape in host 2 has been grayed out. (**c**) Here the sub-landscape in host 2 is emphasized (by blue edges) while the sub-landscape for host 1 is grayed out.

for nearly the whole simulations. However, these subsets are effectively the lower-dimensional landscapes for each host that we originally focused on. A second reason relates to the interacting species case above. In our fixed higher-dimensional landscape it is possible for the fitness to drop (this occurs when the host changes and the new host-plasmid genotypic combination has a lower fitness). The seascape representation avoids such counterintuitive movement (as in Figures S2.1e-h).

With this said, we do think there could be situations where the fixed higher-dimensional landscape would be apposite. Imagine a situation where plasmid-free hosts immigrate into the system at a constant rate. Upon conjugation, suppose the transconjugant rises to prominence only if it has greater fitness than the plasmid donor. In a situation where the processes of intraspecific selection and interspecific competition were strong relative to the processes of mutation and conjugation, we think it would be appropriate to model evolution as an adaptive walk on the fixed higher-dimensional landscape.

An additional situation involves a case where the plasmid is treated as a kind of molecular symbiont associated with a host bacterium. In this light, conjugation of the plasmid between hosts is a kind of "partner-switching" in which standard cross-partner interactions could occur (see Heath 2010; Dunn et al. 2021; Sørensen et al. 2021). Once the plasmid is inside a new host, different host variants could be defined by chromosomal mutations. Suppose we focused on how the host chromosome and plasmid mutate after some bacterium had acquired a new plasmid. Like the interspecies case in Figure S2.1, a set of meta-genotypes (each linking plasmid genotype and host genotype) could be specified for any set of mutations in the plasmid and chromosome. Unlike the interspecies case above, there would be a *single* landscape indicating the fitness of the host variant bearing the relevant plasmid variant. Again, if the process of mutation was weak relative to selection, evolution of such a system could be represented as an adaptive walk on the fixed meta-genotypic landscape. In situations such as this (and the preceding paragraph) we could employ previously developed techniques to study host-plasmid coevolution as an adaptive walk (Draghi and Plotkin 2013; Bank et al. 2016). Such cases form interesting directions for future work.

Supplementary section 3: Derivation of the approximate growth rate

Here, we describe the metric that was used to estimate the growth rates of genotypes in the batch assays performed in this study. Because more genotypes survived at lower antibiotic concentrations than at higher antibiotic concentrations, the number of genotypes that actually competed together decreased as the antibiotic concentration increased. Therefore, as the antibiotic concentration increased the time interval for active growth for each genotype was extended due to having less competitors and more access to resources (given that the initial density per genotype was relatively constant across the gradient). Given that the growth interval increased as the antibiotic concentration increased, we use the derivation here to systematically correct the growth time across the gradient to account for this. If this correction is not applied, the growth rates are systematically under-estimated at lower concentrations where the growth time is shorter due to increased competition for resources. Here we derive an approximate growth rate metric that accounts for the variable growth period across the gradient by normalizing changes in genotype counts to that of the most resistant type in a given assay. Calculating this approximate growth rate metric across antibiotic concentrations generated a standard dose-response curve for each genotype as described in the materials and methods.

Let c^* be the highest concentration where the genotype with the highest resistance does not see a drop in growth. That is, for $c > c^*$, the most resistant genotype will drop in its estimated growth rate, indicative that the genotype was affected by the antibiotic. We label this most resistant genotype as g'. We let the calculated growth rate of genotype g at concentration c be given by m_g^c. To calculate this growth rate, we used a time period of 24 hours; however, the culture may have been growing for less than 24 hours, which will lead to misestimation of the calculated growth rate. We let μ_g^c be the *true* growth rate of genotype g at concentration c. Now at concentration c^*, we will assume that the calculated growth rate and true growth rate are equal for genotype g'

$$\mu_{g'}^{c^*} = m_{g'}^{c^*},$$

which is simply assuming that growth is occurring during the full $T = 24$ hours.

For the time being, we will assume that the true growth rate of genotype g' remains constant at lower drug concentrations.

Now, consider some concentration $c < c^*$. At this concentration, the most resistant genotype g' will realize its maximum true growth rate $\mu_{g'}^{c^*}$. Suppose that the duration of the growth interval at concentration c is given by t_c, at which time resources have been depleted such that all genotypes cease growing. Then the cell count of genotype g' at the end of the growth period is given by:

$$n_{g'}^{c}(t_c) = n_{g'}^{c}(0)e^{\mu_{g'}^{c^*}(t_c)}$$

Solving for the duration of the growth period, we have:

$$t_c = \frac{1}{\mu_{g'}^{c^*}} \ln \frac{n_{g'}^{c}(t_c)}{n_{g'}^{c}(0)}$$

If $n_*^c(t)$ is the total cell count in the assay at time t, and $b_g^c(t)$ is the proportion of barcodes associated with genotype g in concentration c at time t, then we have

$$t_c = \frac{1}{\mu_{g'}^{c^*}} \ln \frac{n_*^c(t_c) b_{g'}^c(t_c)}{n_*^c(0) b_{g'}^c(0)}$$

Further, we assume that the number and proportion of cells does not change from the end of the growth interval t_c to some later time T. Therefore, we can equivalently substitute T for t_c:

$$t_c = \frac{1}{\mu_{g'}^{c^*}} \ln \frac{n_*^c(T) b_{g'}^c(T)}{n_*^c(0) b_{g'}^c(0)}$$

Now, let us turn to consider any arbitrary genotype (as opposed to the most resistant genotype g'). The count of genotype g at the end of the growth interval is given by:

$$n_g^c(t_c) = n_g^c(0)e^{\mu_g^c(t_c)}$$

The true growth rate of an arbitrary genotype — our quantity of interest — can be expressed as:

$$\mu_g^c = \frac{1}{t_c} \ln \frac{n_*^c(t_c) b_g^c(t_c)}{n_*^c(0) b_g^c(0)}$$

Or, assuming that the number and proportion of cells does not change from t_c to T:

$$\mu_g^c = \frac{1}{t_c} \ln \frac{n_*^c(T) b_g^c(T)}{n_*^c(0) b_g^c(0)}$$

After substituting the above expression for t_c and rearranging terms we obtain the following:

$$\mu_g^c = \left(\frac{\ln \frac{n_*^c(T) b_g^c(T)}{n_*^c(0) b_g^c(0)}}{\ln \frac{n_*^c(T) b_{g'}^c(T)}{n_*^c(0) b_{g'}^c(0)}} \right) \mu_{g'}^{c^*}. \qquad [3.1]$$

This gives us an equation for the growth rate of a genotype in terms of the total cell count, barcode proportions, and a fixed reference time T, which can be chosen arbitrarily (so long as it falls after the end of the growth interval). We use T = 24 hours in our analysis. Critically, this method of calculating growth rate does not depend on the duration of the growth interval itself. Effectively, by assuming that all genotypes grow for the same amount of time in a given assay, we normalize the change in genotype counts to that of the most resistant type.

To derive equation [3.1], we assumed that growth for genotype g' is occurring for a full 24 hours when considering concentration c^*. However, this assumption is likely misplaced. Suppose that the actual growth time is t^*, such that we have the following:

$$t^* = (f)T$$

where the actual growth time is some fraction f of the full time period ($T = 24$ hours). Therefore,

$$n_{g'}^{c^*}(t^*) = n_{g'}^{c^*}(0)e^{\mu_{g'}^{c^*}(t^*)}$$

and

$$\mu_{g'}^{c^*} = \frac{1}{t^*}\ln\frac{n_{g'}^{c^*}(t^*)}{n_{g'}^{c^*}(0)}$$

So, substituting t^* with $(f)T$ where $T = 24$ and assuming that the number of cells does not change from t^* to T (the same assumption as above):

$$\mu_{g'}^{c^*} = \frac{1}{f}\left\{\frac{1}{24}\ln\frac{n_{g'}^{c^*}(24)}{n_{g'}^{c^*}(0)}\right\}$$

Thus, the connection between the measured growth rate $m_{g'}^{c^*}$ and the actual growth rate $\mu_{g'}^{c^*}$, is off by a factor:

$$\mu_{g'}^{c^*} = \frac{1}{f}\left(m_{g'}^{c^*}\right)$$

For simplicity, we assume $f = 1$ in our data analysis.

Supplementary section 4: Probability of a mutation being the most resistant (simulating gradient selection)

Here, we derive the probability that the population shifts from the ancestral genotype (hereafter focal genotype) to a neighboring single mutant genotype at the end of a time step. Our mathematical framework is centered on a case of selection for maximal resistance among a random set of mutants. Such a situation would apply if a population of cells with the ancestral genotype grew (and generated mutants) and then was exposed (as subpopulations) to a series of drug concentrations. Focusing on the highest drug concentration for which there was subsequent growth, an isolate from this subpopulation becomes the genotype for the next time step (which could be a mutant that was more resistant than the original ancestor). This setup is similar to standard directed evolution studies involving selection on a drug gradient, which has been shown previously to mimic the natural evolution of the beta-lactamase enzyme (Barlow and Hall 2002).

Let μ be the probability a mutation arises in the focal genotype as a descendent cell is generated. We assume that the probability of mutation is small such that we ignore double mutation events and assume that most individuals within the population have the focal genotype (i.e., mutants are rare and have only a

single mutation). All possible single mutant genotypes are denoted by the set $\mathbf{M} \equiv \{m_1, m_2, m_3, \dots m_y\}$ (where $|\mathbf{M}|$ is the number of mutants).

Consider a population that is initially fixed for the focal genotype i. In the process of creating a population of descendant cells, the focal genotype is copied n times (independently), and the probability that a mutation occurs in a descendent is given by μ. Focusing on one mutant genotype (e.g., m_j), the probability that there are k descendants with this genotype among the n descendant cells will be:

$$\pi_k(m_j) = \binom{n}{k} (\mu)^k (1-\mu)^{n-k}$$

The probability that there are one or more m_j individuals among the descendants of the focal genotype is then:

$$1 - \pi_0(m_j) \approx 1 - e^{-\mu n}$$

We denote this probability as $\pi^*(m_j)$.

After mutation, the descendent undergoes growth followed by selection across an antibiotic gradient. Here we assume that there is enough growth of the population such that if a mutant genotype is present, it is distributed across the entire antibiotic gradient. For a given focal genotype i, and a set of neighboring mutant genotypes labeled $\mathbf{M}$, we denote the genotypes in the set $\mathbf{M}$ that are more resistant than the focal genotype as $\mathbf{H_M}(i)$. First, we start with the probability that the most resistant individual cell has is the focal genotype i. In this case, the probability is:

$$p_i = \prod\nolimits_{m \in \mathbf{H_M}(i)} (1 - \pi^*(m))$$

If the focal genotype is more resistant than all mutants (i.e., the set $\mathbf{H_M}(i)$ is empty), then this probability is defined to be one. If a mutant neighbor is more resistant than the focal genotype, this probability becomes less than one. Generally, when the focal genotype is less resistant, it is less probable for the focal genotype to be the most resistant, and thus less probable for the population to stay at this genotype.

Next, we turn to the probability that the most resistant individual has a particular genotype m_j from the set of mutants $\mathbf{M}$. Here we need to introduce more notation. For a given genotype g, and a set of genotypes labeled $\mathbf{S}$, we denote the genotypes in $\mathbf{S}$ that are equally resistant to genotype g as $\mathbf{E_S}(g)$. If genotype g is in the set $\mathbf{S}$, we will define $\mathbf{E_S}(g)$ to *not* include genotype g (i.e., $g \notin \mathbf{E_S}(g)$). Therefore, $\mathbf{E_S}(g)$ is the set of genotypes with equivalent resistance to genotype g, *other than g itself.* Consider a mutant genotype m_j, which has higher resistance than the focal genotype i. We denote the set of other mutants that have higher and equivalent resistance to genotype m_j as $\mathbf{H_M}(m_j)$ and $\mathbf{E_M}(m_j)$, respectively. The probability that the most resistant individual has genotype m_j is chosen is given by:

$$p_{m_j} = \pi^*(m_j) \prod_{m \in \mathbf{H_M}(m_j)} (1 - \pi^*(m)) \left[\sum_{\mathbf{S} \subseteq \mathbf{E_M}(m_j)} \frac{\left(\prod_{m' \in \mathbf{S}} \{\pi^*(m')\}\right) \left(\prod_{m'' \in \mathbf{E_M}(m_j) - \mathbf{S}} \{1 - \pi^*(m'')\}\right)}{1 + |\mathbf{S}|} \right]$$

If there are never any ties between genotypes with regards to resistance, then $|\mathbf{E_M}(m_j)| = 0$ and the above equation simplifies to:

$$p_{m_j} = \pi^*(m_j) \prod_{m \in \mathbf{H_M}(m_j)} (1 - \pi^*(m))$$

If a particular mutant m_j is the most resistant mutant of the focal genotype ($|\mathbf{H_M}(m_j)| = 0$) then the probability of picking the mutant is $\pi^*(m_j)$ which depends only on the population size and the mutation rate (see above). More generally, the probability of selecting mutant m_j covaries positively with its ranking in the

set (i.e., the more resistant this mutant genotype is relative to the other mutants of the focal genotype, the more likely it is to be selected).

Supplemental Tables

SI Table 1: Primers used for Site-Directed Mutagenesis. The mutagenic primer is labelled with an asterisk. If an amino acid is being mutated, the codon is underlined. The nucleotide being mutated is **bolded**.

Mutation	Primer	Primer orientation	Sequence (5’ -> 3’)
g4205a	pOK84	Forward*	**A**AGCGGATACATATTTGAATGTATTTAGAAAAATAA
	pOK85	Reverse	ATGAGACAATAACCCTGATAAATGCTTC
A42G	pOK80	Forward*	T**C**CACCCAACTGATCTTCAGCATCT
	pOK81	Reverse	CGAGTGGGTTACATCGAACTG
E104K	pOK78	Forward*	CT**T**AACCAAGTCATTCTGAGAATAGTGTATG
	pOK79	Reverse	TACTCACCAGTCACAGAAAAGCA
M182T	pOK82	Forward*	C**G**TCGTGGTGTCACGCTCG
	pOK83	Reverse	CCTGCAGCAATGGCAACAACGTTGC
G238S	pOK75	Forward*	CTCAC**T**GGCTCCAGATTTATCAGC
	pOK74	Reverse	CGTGGGTCTCGCGGTATC

SI Table 2 : Engineered variants using site-directed mutagenesis.

Variant	Number of mutations
g4205a	1
A42G	1
E104K	1
M182T	1
G238S	1
g4205a, A42G	2
g4205a, E104K	2
g4205a, M182T	2
g4205a, G238S	2
A42G, E104K	2
A42G, M182T	2
A42G, G238S	2
E104K, M182T	2
E104K, G238S	2
M182T, G238S	2
g4205a, A42G, E104K	3
g4205a, A42G, M182T	3
g4205a, A42G, G238S	3
g4205a, E104K, M182T	3
g4205a, E104K, G238S	3
g4205a, M182T, G238S	3
A42G, E104K, M182T	3
A42G, E104K, G238S	3
A42G, M182T, G238S	3
E104K, M182T, G238S	3
g4205a, A42G, E104K, M182T	4
g4205a, A42G, E104K, G238S	4

g4205a, A42G, M182T, G238S	4
g4205a, E104K, M182T, G238S	4
A42G, E104K, M182T, G238S	4
g4205a, A42G, E104K, M182T, G238S	5

SI Table 3 : Primers used for Sanger sequencing.

**Primer**	**Sequence region**	**Sequence (5’ -> 3’)**
pOK6	280th amino acid to 600 downstream nucleotides	CAGGCAACTATGGATGAACG
pOK10	50th to 286th amino acid	CCTTCCTGTTTTTGCTCACC
pOK38	Promotor region to 185th amino acid	GAGGATGACGATGAGCGCAT

SI Table 4 : Primers used for creating the barcode fragment. The NcoI and NsiI restriction sites are **bolded.** The homologous nucleotides used for creating the double stranded fragment are underlined.

**Primer**	**Sequence (5’ -> 3’)**
pOK67	CGGACCGCTGGACGTATCTTAGTTTTCTCGAGTAAGATCCAT**CCATG**GTCTGTC<br>ACACCGAGAGGCTAGGCAGTTGCGCGCGTACGNNNNNNNNNNNNNNNNNNNNAC<br>CGGTCCGGTAATCGAACTGGGCGAGACATCCCAGCTTAGCT**ATGCAT**TCACTA<br>GAGGACGCGTGTCCACGTGAAGACATCCCAGCGCTTGA
pOK68	TCAAGCGCTGGGATGTCTTC

SI Table 5 : Initial cell density of each library before selection in CTX.

**Species**	**Number of replicates**	**Average initial density (cfu ml^{-1})**	**Standard error**
Ec	6	4.07×10^{5}	2.67×10^{4}
Kp	6	3.21×10^{5}	2.99×10^{4}
Se	6	6.07×10^{5}	3.41×10^{4}

SI Table 6 : Final cell density (cfu ml^{-1}) from each library selection. The concentrations where sequencing data was obtained are **bolded** for each species. A portion of the lower concentration was not submitted for sequencing given the resistance level of the ancestral genotype, TEM-1. Test tubes that were not turbid (NT) after the 24 h incubation are designated.

**CTX concentration (μg ml^{-1})**	**Ec**	**Kp**	**Se**
0.0000	**2.44×10^{9}**	**3.00×10^{9}**	**2.84×10^{9}**
0.00393	2.28×10^{9}	3.88×10^{9}	1.91×10^{9}
0.0056	2.32×10^{9}	3.32×10^{9}	2.26×10^{9}
0.0079	1.72×10^{9}	3.52×10^{9}	2.39×10^{9}
0.0112	2.00×10^{9}	3.22×10^{9}	2.33×10^{9}
0.0158	1.42×10^{9}	2.24×10^{9}	2.15×10^{9}
0.0223	1.06×10^{9}	2.88×10^{9}	1.97×10^{9}
0.0315	**1.06×10^{9}**	**2.36×10^{9}**	**2.37×10^{9}**
0.0445	**9.80×10^{8}**	**2.18×10^{9}**	**1.70×10^{9}**
0.0629	**9.80×10^{8}**	**3.48×10^{9}**	**1.92×10^{9}**
0.0889	**1.06×10^{9}**	**3.04×10^{9}**	**2.01×10^{9}**
0.1257	**1.32×10^{9}**	**3.44×10^{9}**	**1.74×10^{9}**
0.1777	**1.32×10^{9}**	**3.88×10^{9}**	**1.55×10^{9}**
0.2513	**1.46×10^{9}**	**3.20×10^{9}**	**2.07×10^{9}**
0.3553	**1.26×10^{9}**	**3.08×10^{9}**	**1.92×10^{9}**
0.5024	**1.50×10^{9}**	**4.00×10^{9}**	**1.91×10^{9}**

0.7104	**1.24 x 10^9**	**3.12 x 10^9**	**1.92 x 10^9**
1.0045	**1.86 x 10^9**	**3.10 x 10^9**	**2.05 x 10^9**
1.42	**1.26 x 10^9**	**2.78 x 10^9**	**1.71 x 10^9**
2.01	**1.32 x 10^9**	**3.04 x 10^9**	**2.74 x 10^9**
2.84	**7.50 x 10^8**	**3.76 x 10^9**	**2.02 x 10^9**
4.02	**1.07 x 10^9**	**3.36 x 10^9**	**1.98 x 10^9**
5.68	**1.08 x 10^9**	**2.68 x 10^9**	**1.44 x 10^9**
8.03	**1.56 x 10^9**	**3.24 x 10^9**	**2.72 x 10^9**
11.35	**1.42 x 10^9**	**3.92 x 10^9**	**1.82 x 10^9**
16.05	**2.54 x 10^9**	**3.40 x 10^9**	**2.47 x 10^9**
22.69	**1.04 x 10^9**	**4.44 x 10^9**	**2.08 x 10^9**
32.08	**1.46 x 10^9**	**3.20 x 10^9**	**1.94 x 10^9**
45.36	**1.36 x 10^9**	**3.68 x 10^9**	**1.41 x 10^9**
64.14	**1.26 x 10^9**	**2.64 x 10^9**	**1.55 x 10^9**
90.69	**1.37 x 10^9**	**1.81 x 10^9**	**1.16 x 10^9**
128.24	**4.80 x 10^9**	**2.63 x 10^9**	**1.58 x 10^9**
181.33	**1.14 x 10^9**	NT	**7.00 x 10^8**
256.4	NT	NT	**1.15 x 10^9**
362.55	NT	NT	**1.92 x 10^8**
512.65	NT	NT	NT
724.89	NT	NT	NT
1024.99	NT	NT	NT
1449.34	NT	NT	NT
2049.37	NT	NT	NT

SI Table 7 : Primers used for library amplification and sequencing. The nucleotides that are homologous to the plasmid are **bolded.** The nucleotides that are homologous to the indexing primers are underlined. The 9-bp index used for multiplexing the samples is represented with N nucleotides and are sequence specific depending on the sample.

Primer	Purpose	Sequence (5' -> 3')
pOK55	PCR round 1 forward primer	CCGCGTGATTACGAGTCG**GCAGCAGATTACGCGCAGAA**
pOK56	PCR round 1 reverse primer	GGGTTAGCAAGTGGCAGCCT**AGCGCTGGGATGTCTCG**
	PCR round 2 forward primer	AATGATACGGCGACCACCGAGATCTACACNNNNNNNNNCCGCGTGATTACGAGTCG
	PCR round 2 reverse primer	CAAGCAGAAGACGGCATACGAGATNNNNNNNNNGGGTTAGCAAGTGGCAGCCT
pOK57	Custom read 1 primer	TCACACCGAGAGGCTAGGCAGTTGCGCGCGTACG
pOK59	Custom read 2 primer	GCTGGGATGTCTCGCCCAGTTCGATTACCGGACCGGT

SI Table 8 : Genotype to barcode map.

Variant	Barcode 1	Barcode 2	Barcode 3
Wild-type TEM-1	GATGGCCTTTTGCCGGTT	ATTCGTAAACTTCTGGTT	GAGTCGTGCATTGAGTTC
g4205a	GTATTAGTTTTACTTTAG	TTGAGTCCCGAGGGTGGT	TACTTTTAAACTATAAGA
A42G	CAGTGAGTCAGATATCTT	GTAGCCATTTACTTCTGT	TTCGCATTATATTCCGTC
E104K	TTGGCGCCTTCTCTTCTG	GGAGCAGTAGAGGTGGTA	ATAAAGGATGTGACTGTA
M182T	CCTAGCAGCTCGTAAGAG	TCCAAAGGGTGGCACGAG	TGTGATTTACACACGTCC

G238S	ACTGCGTTTTAATATTTT	CCAACTGTAAGCCTATTT	CAGGGTCATACGAGCTTC
g4205a, A42G	CTCATTTTAGACTTCGTT	CATTCATATTAAAGTTTG	CAGGTTTTAGCATATGCC
g4205a, E104K	CGACAACGTATCAAGCTC	TCCGACCATTAAGGGTTA	GCTGGTCCGATCAGATAT
g4205a, M182T	AGGGATCTGGAGTAGGTC	AATGTGCGTTAATAGATT	TATCATAGTGAGTTCCAT
g4205a, G238S	ATAAGGTTTGTTTCCCTG	AATTTAAGTATAGAGGGG	TAGGTTAATTCTCGGTGA
A42G, E104K	ATTAGATTTATTAATATG	GTTTCCTCTAAAGATTTC	TTTTTCCTCCGCTCTGGT
A42G, M182T	CGTACCCCTTGCTGGTGG	AGAACTTGGTAACGGGGC	TTGGGACCTCTTTGGGTA
A42G, G238S	CAGCTGGTTGGTTCTCTA	ATAGTTATTTTGGAACTA	ACTACAGTAATAGTGCAT
E104K, M182T	GCTTCCTTTATTTGTTTA	GCGAGATGATTAGAGAGA	GTGTGAGACGCAGTTTAG
E104K, G238S	TGTATTGGTTAACGTTAC	TAACGCGAGTCGTAATCT	GAGGGTGTGATTAGCAAT
M182T, G238S	GATCCGATGATAGTAGTT	TATCCGTCCTCGGCAGAG	TCCCTAGCATGGATTGGC
g4205a, A42G, E104K	ATAAATATGTGGTCCCTG	CACCATCCTACAACTAAA	AATCATTCAAATCGAAGA
g4205a, A42G, M182T	TTAACACATGGTATTTAC	TTGTTTATTTACCGGACT	AAAGTTTGAGGAATAACG
g4205a, A42G, G238S	AAGTGTTCGCATTGCAAG	ACAGGAGACGGTATCTTT	TTGAAATGCTTTCGGTTA
g4205a, E104K, M182T	AGTCGTGTGGGGGCCTAC	GCGTCCTCGAGTCTTTAC	TTTGGACCACTTTTCTGT
g4205a, E104K, G238S	AACGGTTGGACCGAGCGG	CCATTTGATTTTAAGCTC	TTGCGGAAGTGGTCGTGG
g4205a, M182T, G238S	TTTGTTGCTCTTTCGATG	TCCGGTACGATTACAACG	AACCTAATTCTTAACGGA
A42G, E104K, M182T	GTGCGAAAGCATTACACT	TTGCATAGTTTCATAATA	GCAGTATGCGGAAAAGCT
A42G, E104K, G238S	AGCATGCTCTGCCGAGAA	TCCACGTACATAAATGTT	ACTGCTTAGCAGTTTGTC
A42G, M182T, G238S	GGCAGGTGAATCTACCAG	TTTAGCCCCATCACTAAC	TTTGTGTAGGTACTATCC
E104K, M182T, G238S	CAATATACTCTGTACTAA	CATCTAATTTATTGGGTA	TAGTGCTTGTTCAGGGGT
g4205a, A42G, E104K, M182T	GCATCGCTCTCATGGGTA	CATAGTCACCGGCTAGAT	GAGAGGCTAAGGTGAAAC
g4205a, A42G, E104K, G238S	TATTTGAGTTATTAGTTC	ACGTAAAGTAAGACTTCA	AGTGCAGGTTTAAATACT
g4205a, A42G, M182T, G238S	TTAGCTTGGTTTCTGTCT	GATAGGGTATATTGGCAC	TAGCATCGGGTCAGGGCG
g4205a, E104K, M182T, G238S	ATATTTTACCGTCTTAAA	GCCGCGGCGTGTGTGGTT	GTAGTAGGTGTCTCAGAC
A42G, E104K, M182T, G238S	ACGAACTTTGCTTTCTTT	GAAAACATACGGCGTGGT	AGTCATGGCATTATGAAA
g4205a, A42G, E104K, M182T, G238S	CTGTTTTTGAACTTGAAG	TGAAGCACGTAAACTATC	TTGGTTGCACCAGACATT

SI Table 9: The three-parameter estimates (inflection point, steepness, and upper asymptote) from the dose-response curve fitting for each barcode-genotype-species combination.

Species	Shorthand	Barcode	Deviant (Outlier)	Inflection point	Slope	Upper asymptote
Ec	Wild-type TEM-1	GATGGCCTTTTGCCGGTT		0.12	3.25	0.48

Ec	Wild-type TEM-1	GAGTCGTGCATTGAGTTC		0.12	3.67	0.48
Ec	Wild-type TEM-1	ATTCGTAAACTTCTGGTT	x	0.12	3.4	0.47
Ec	g4205a	GTATTAGTTTTACTTTAG		0.13	3.39	0.44
Ec	g4205a	TACTTTTAAACTATAAGA		0.12	3.67	0.44
Ec	g4205a	TTGAGTCCCGAGGGTGGT	x	0.1	4.28	0.4
Ec	A42G	CAGTGAGTCAGATATCTT		0.12	3.74	0.48
Ec	A42G	TTCGCATTATATTCCGTC		0.12	3.72	0.47
Ec	A42G	GTAGCCATTTACTTCTGT	x	0.13	3.23	0.48
Ec	E104K	GGAGCAGTAGAGGTGGTA		0.2	3.53	0.47
Ec	E104K	ATAAAGGATGTGACTGTA		0.21	3.5	0.46
Ec	E104K	TTGGCGCCTTCTCTTCTG	x	0.22	2.59	0.48
Ec	M182T	CCTAGCAGCTCGTAAGAG		0.12	3.37	0.49
Ec	M182T	TCCAAAGGGTGGCACGAG		0.12	3.32	0.49
Ec	M182T	TGTGATTTACACACGTCC	x	0.11	3.35	0.48
Ec	G238S	ACTGCGTTTTAATATTTT		1	3.55	0.42
Ec	G238S	CCAACTGTAAGCCTATTT		1.11	3.33	0.42
Ec	G238S	CAGGGTCATACGAGCTTC	x	1.13	3.24	0.42
Ec	g4205a, A42G	CTCATTTTAGACTTCGTT		0.14	3.56	0.46
Ec	g4205a, A42G	CATTCATATTAAAGTTTG		0.13	3.61	0.45
Ec	g4205a, A42G	CAGGTTTTAGCATATGCC	x	0.14	2.95	0.47
Ec	g4205a, E104K	TCCGACCATTAAGGGTTA		0.27	3.1	0.42
Ec	g4205a, E104K	GCTGGTCCGATCAGATAT		0.28	3.42	0.42
Ec	g4205a, E104K	CGACAACGTATCAAGCTC	x	0.29	2.65	0.43
Ec	g4205a, M182T	AATGTGCGTTAATAGATT		0.13	3.31	0.5
Ec	g4205a, M182T	TATCATAGTGAGTTCCAT		0.13	3.25	0.47
Ec	g4205a, M182T	AGGGATCTGGAGTAGGTC	x	0.13	2.97	0.49
Ec	g4205a, G238S	ATAAGGTTTGTTTCCCTG		1.05	3.6	0.34
Ec	g4205a, G238S	TAGGTTAATTCTCGGTGA		1	3.39	0.34
Ec	g4205a, G238S	AATTTAAGTATAGAGGGG	x	1.05	3.81	0.35
Ec	A42G, E104K	GTTTCCTCTAAAGATTTC		0.44	3.42	0.46
Ec	A42G, E104K	TTTTTCCTCCGCTCTGGT		0.47	2.81	0.47
Ec	A42G, E104K	ATTAGATTTATTAATATG	x	0.37	3.82	0.46
Ec	A42G, M182T	AGAACTTGGTAACGGGGC		0.12	3.37	0.48
Ec	A42G, M182T	TTGGGACCTCTTTGGGTA		0.12	3.48	0.48
Ec	A42G, M182T	CGTACCCCTTGCTGGTGG	x	0.12	2.78	0.51
Ec	A42G, G238S	ATAGTTATTTTGGAACTA		4.2	2.47	0.46
Ec	A42G, G238S	ACTACAGTAATAGTGCAT		4.58	2.26	0.47
Ec	A42G, G238S	CAGCTGGTTGGTTCTCTA	x	5.13	2.09	0.48
Ec	E104K, M182T	GCTTCCTTTATTTGTTTA		0.21	3.34	0.47

Ec	E104K, M182T	GTGTGAGACGCAGTTTAG		0.21	3.44	0.47
Ec	E104K, M182T	GCGAGATGATTAGAGAGA	x	0.18	3.83	0.47
Ec	E104K, G238S	TGTATTGGTTAACGTTAC		7.03	3.12	0.41
Ec	E104K, G238S	GAGGGTGTGATTAGCAAT		7.05	2.68	0.41
Ec	E104K, G238S	TAACGCGAGTCGTAATCT	x	9.26	2.51	0.42
Ec	M182T, G238S	TATCCGTCCTCGGCAGAG		2.7	3.01	0.5
Ec	M182T, G238S	TCCCTAGCATGGATTGGC		2.74	3	0.5
Ec	M182T, G238S	GATCCGATGATAGTAGTT	x	2.69	2.63	0.49
Ec	g4205a, A42G, E104K	ATAAATATGTGGTCCCTG		0.62	2.93	0.45
Ec	g4205a, A42G, E104K	CACCATCCTACAACTAAA		0.65	2.85	0.45
Ec	g4205a, A42G, E104K	AATCATTCAAATCGAAGA	x	0.58	3.42	0.44
Ec	g4205a, A42G, M182T	TTAACACATGGTATTTAC		0.14	2.91	0.48
Ec	g4205a, A42G, M182T	TTGTTTATTTACCGGACT		0.14	3.55	0.49
Ec	g4205a, A42G, M182T	AAAGTTTGAGGAATAACG	x	0.13	3.74	0.47
Ec	g4205a, A42G, G238S	ACAGGAGACGGTATCTTT		6.78	2.49	0.44
Ec	g4205a, A42G, G238S	TTGAAATGCTTTCGGTTA		6.46	2.66	0.44
Ec	g4205a, A42G, G238S	AAGTGTTCGCATTGCAAG	x	7.03	2.42	0.44
Ec	g4205a, E104K, M182T	GCGTCCTCGAGTCTTTAC		0.27	2.95	0.47
Ec	g4205a, E104K, M182T	TTTGGACCACTTTTCTGT		0.27	3.14	0.46
Ec	g4205a, E104K, M182T	AGTCGTGTGGGGGCCTAC	x	0.29	2.59	0.48
Ec	g4205a, E104K, G238S	AACGGTTGGACCGAGCGG		6.69	2.12	0.35
Ec	g4205a, E104K, G238S	TTGCGGAAGTGGTCGTGG		6.24	2.23	0.35
Ec	g4205a, E104K, G238S	CCATTTGATTTTAAGCTC	x	6.31	2.46	0.33
Ec	g4205a, M182T, G238S	TTTGTTGCTCTTTCGATG		4.42	2.01	0.5
Ec	g4205a, M182T, G238S	AACCTAATTCTTAACGGA		4.85	2.46	0.5
Ec	g4205a, M182T, G238S	TCCGGTACGATTACAACG	x	5.27	2.39	0.53
Ec	A42G, E104K, M182T	TTGCATAGTTTCATAATA		0.38	3.52	0.47

Ec	A42G, E104K, M182T	GCAGTATGCGGAAAAGCT		0.39	3.04	0.48
Ec	A42G, E104K, M182T	GTGCGAAAGCATTACACT	x	0.46	3.21	0.5
Ec	A42G, E104K, G238S	TCCACGTACATAAATGTT		32.15	2.07	0.46
Ec	A42G, E104K, G238S	ACTGCTTAGCAGTTTGTC		33.65	2.22	0.46
Ec	A42G, E104K, G238S	AGCATGCTCTGCCGAGAA	x	58.76	3.24	0.45
Ec	A42G, M182T, G238S	GGCAGGTGAATCTACCAG		5.39	2.29	0.49
Ec	A42G, M182T, G238S	TTTGTGTAGGTACTATCC		4.8	2.47	0.48
Ec	A42G, M182T, G238S	TTTAGCCCCATCACTAAC	x	6.21	2.55	0.51
Ec	E104K, M182T, G238S	CAATATACTCTGTACTAA		34.19	1.82	0.47
Ec	E104K, M182T, G238S	CATCTAATTTATTGGGTA		35.52	1.75	0.47
Ec	E104K, M182T, G238S	TAGTGCTTGTTCAGGGGT	x	31	1.8	0.48
Ec	g4205a, A42G, E104K, M182T	GCATCGCTCTCATGGGTA		0.6	2.65	0.48
Ec	g4205a, A42G, E104K, M182T	GAGAGGCTAAGGTGAAAC		0.55	2.76	0.48
Ec	g4205a, A42G, E104K, M182T	CATAGTCACCGGCTAGAT	x	0.67	3.22	0.49
Ec	g4205a, A42G, E104K, G238S	TATTTGAGTTATTAGTTC		45.24	2.41	0.41
Ec	g4205a, A42G, E104K, G238S	AGTGCAGGTTTAAATACT		47.43	2.34	0.41
Ec	g4205a, A42G, E104K, G238S	ACGTAAAGTAAGACTTCA	x	53.16	2.07	0.41
Ec	g4205a, A42G, M182T, G238S	TTAGCTTGGTTTCTGTCT		8.8	2.37	0.49
Ec	g4205a, A42G, M182T, G238S	TAGCATCGGGTCAGGGCG		8.62	2.34	0.49
Ec	g4205a, A42G, M182T, G238S	GATAGGGTATATTGGCAC	x	7.71	2.42	0.49
Ec	g4205a, E104K, M182T, G238S	ATATTTTACCGTCTTAAA		167.29	2.35	0.48
Ec	g4205a, E104K, M182T, G238S	GTAGTAGGTGTCTCAGAC		203.43	2.58	0.48
Ec	g4205a, E104K, M182T, G238S	GCCGCGGCGTGTGTGGTT	x	96.01	1.47	0.48

Ec	A42G, E104K, M182T, G238S	GAAAACATACGGCGTGGT		66.33	1.36	0.48
Ec	A42G, E104K, M182T, G238S	AGTCATGGCATTATGAAA		63.07	1.7	0.47
Ec	A42G, E104K, M182T, G238S	ACGAACTTTGCTTTCTTT	x	107.15	1.45	0.5
Ec	g4205a, A42G, E104K, M182T, G238S	TGAAGCACGTAAACTATC		528.76	1.56	0.48
Ec	g4205a, A42G, E104K, M182T, G238S	TTGGTTGCACCAGACATT		2012.02	0.72	0.48
Ec	g4205a, A42G, E104K, M182T, G238S	CTGTTTTTGAACTTGAAG	x	205.94	1.54	0.48
Kp	Wild-type TEM-1	GATGGCCTTTTGCCGGTT		0.04	5.44	0.56
Kp	Wild-type TEM-1	ATTCGTAAACTTCTGGTT		0.04	7.15	0.56
Kp	Wild-type TEM-1	GAGTCGTGCATTGAGTTC	x	0.04	6.54	0.56
Kp	g4205a	TTGAGTCCCGAGGGTGGT		0.04	7.06	0.55
Kp	g4205a	TACTTTTAAACTATAAGA		0.04	27.18	0.52
Kp	g4205a	GTATTAGTTTTACTTTAG	x	0.04	6.75	0.52
Kp	A42G	GTAGCCATTTACTTCTGT		0.04	7.86	0.51
Kp	A42G	TTCGCATTATATTCCGTC		0.04	6.24	0.53
Kp	A42G	CAGTGAGTCAGATATCTT	x	0.04	8.07	0.54
Kp	E104K	TTGGCGCCTTCTCTTCTG		0.08	5.91	0.55
Kp	E104K	GGAGCAGTAGAGGTGGTA		0.07	5.87	0.57
Kp	E104K	ATAAAGGATGTGACTGTA	x	0.08	5.44	0.54
Kp	M182T	CCTAGCAGCTCGTAAGAG		0.04	7.44	0.55
Kp	M182T	TGTGATTTACACACGTCC		0.04	7.16	0.56
Kp	M182T	TCCAAAGGGTGGCACGAG	x	0.03	7.41	0.51
Kp	G238S	ACTGCGTTTTAATATTTT		0.4	4.5	0.55
Kp	G238S	CCAACTGTAAGCCTATTT		0.41	4.66	0.55
Kp	G238S	CAGGGTCATACGAGCTTC	x	0.56	3.55	0.62
Kp	g4205a, A42G	CTCATTTTAGACTTCGTT		0.05	5.65	0.54
Kp	g4205a, A42G	CATTCATATTAAAGTTTG		0.05	7.4	0.53
Kp	g4205a, A42G	CAGGTTTTAGCATATGCC	x	0.05	6.49	0.55
Kp	g4205a, E104K	CGACAACGTATCAAGCTC		0.11	5.95	0.54
Kp	g4205a, E104K	TCCGACCATTAAGGGTTA		0.11	6.13	0.55
Kp	g4205a, E104K	GCTGGTCCGATCAGATAT	x	0.11	5.81	0.54
Kp	g4205a, M182T	AATGTGCGTTAATAGATT		0.04	11.85	0.52
Kp	g4205a, M182T	TATCATAGTGAGTTCCAT		0.04	6.88	0.54
Kp	g4205a, M182T	AGGGATCTGGAGTAGGTC	x	0.05	5.81	0.64

Kp	g4205a, G238S	ATAAGGTTTGTTTCCCTG		0.38	3.68	0.48
Kp	g4205a, G238S	TAGGTTAATTCTCGGTGA		0.38	4.2	0.48
Kp	g4205a, G238S	AATTTAAGTATAGAGGGG	x	0.38	4.76	0.5
Kp	A42G, E104K	GTTTCCTCTAAAGATTTC		0.24	4.01	0.68
Kp	A42G, E104K	TTTTTCCTCCGCTCTGGT		0.21	6.22	0.58
Kp	A42G, E104K	ATTAGATTTATTAATATG	x	0.14	5.18	0.53
Kp	A42G, M182T	CGTACCCCTTGCTGGTGG		0.04	6.82	0.57
Kp	A42G, M182T	TTGGGACCTCTTTGGGTA		0.04	6.13	0.54
Kp	A42G, M182T	AGAACTTGGTAACGGGGC	x	0.04	8.1	0.53
Kp	A42G, G238S	ATAGTTATTTTGGAACTA		1.79	3.21	0.55
Kp	A42G, G238S	ACTACAGTAATAGTGCAT		2.18	2.14	0.54
Kp	A42G, G238S	CAGCTGGTTGGTTCTCTA	x	1.95	5.41	0.54
Kp	E104K, M182T	GCTTCCTTTATTTGTTTA		0.08	4.26	0.56
Kp	E104K, M182T	GTGTGAGACGCAGTTTAG		0.07	6.52	0.53
Kp	E104K, M182T	GCGAGATGATTAGAGAGA	x	0.06	6.44	0.54
Kp	E104K, G238S	TGTATTGGTTAACGTTAC		2.93	3.18	0.54
Kp	E104K, G238S	TAACGCGAGTCGTAATCT		2.72	4.06	0.55
Kp	E104K, G238S	GAGGGTGTGATTAGCAAT	x	3.15	3.72	0.52
Kp	M182T, G238S	GATCCGATGATAGTAGTT		1.17	4.21	0.55
Kp	M182T, G238S	TATCCGTCCTCGGCAGAG		1.23	4.18	0.56
Kp	M182T, G238S	TCCCTAGCATGGATTGGC	x	1.31	4.56	0.55
Kp	g4205a, A42G, E104K	ATAAATATGTGGTCCCTG		0.28	5.68	0.53
Kp	g4205a, A42G, E104K	AATCATTCAAATCGAAGA		0.27	9.95	0.53
Kp	g4205a, A42G, E104K	CACCATCCTACAACTAAA	x	0.31	5.19	0.54
Kp	g4205a, A42G, M182T	TTAACACATGGTATTTAC		0.05	7.55	0.54
Kp	g4205a, A42G, M182T	AAAGTTTGAGGAATAACG		0.04	7.42	0.54
Kp	g4205a, A42G, M182T	TTGTTTATTTACCGGACT	x	0.06	2.98	0.74
Kp	g4205a, A42G, G238S	ACAGGAGACGGTATCTTT		2.95	3.71	0.55
Kp	g4205a, A42G, G238S	TTGAAATGCTTTCGGTTA		2.84	4.33	0.55
Kp	g4205a, A42G, G238S	AAGTGTTCGCATTGCAAG	x	2.77	6.3	0.51
Kp	g4205a, E104K, M182T	GCGTCCTCGAGTCTTTAC		0.11	5.9	0.54
Kp	g4205a, E104K, M182T	TTTGGACCACTTTTCTGT		0.1	5.58	0.53
Kp	g4205a, E104K, M182T	AGTCGTGTGGGGGCCTAC	x	0.12	6.29	0.54

Kp	g4205a, E104K, G238S	AACGGTTGGACCGAGCGG		3.44	4.11	0.52
Kp	g4205a, E104K, G238S	TTGCGGAAGTGGTCGTGG		3.4	4.48	0.58
Kp	g4205a, E104K, G238S	CCATTTGATTTTAAGCTC	x	2.33	4.63	0.49
Kp	g4205a, M182T, G238S	TTTGTTGCTCTTTCGATG		2.01	3.27	0.54
Kp	g4205a, M182T, G238S	AACCTAATTCTTAACGGA		2.05	3.46	0.55
Kp	g4205a, M182T, G238S	TCCGGTACGATTACAACG	x	2.41	3.51	0.55
Kp	A42G, E104K, M182T	TTGCATAGTTTCATAATA		0.15	5.46	0.55
Kp	A42G, E104K, M182T	GCAGTATGCGGAAAAGCT		0.16	5.34	0.53
Kp	A42G, E104K, M182T	GTGCGAAAGCATTACACT	x	0.16	4.34	0.5
Kp	A42G, E104K, G238S	TCCACGTACATAAATGTT		11.03	3.49	0.55
Kp	A42G, E104K, G238S	ACTGCTTAGCAGTTTGTC		11.42	3.33	0.54
Kp	A42G, E104K, G238S	AGCATGCTCTGCCGAGAA	x	11.9	3.27	0.55
Kp	A42G, M182T, G238S	GGCAGGTGAATCTACCAG		2.32	4.14	0.56
Kp	A42G, M182T, G238S	TTTAGCCCCATCACTAAC		2.37	3.25	0.54
Kp	A42G, M182T, G238S	TTTGTGTAGGTACTATCC	x	2.1	3.07	0.53
Kp	E104K, M182T, G238S	CAATATACTCTGTACTAA		13	3.02	0.55
Kp	E104K, M182T, G238S	TAGTGCTTGTTCAGGGGT		12.1	3.26	0.57
Kp	E104K, M182T, G238S	CATCTAATTTATTGGGTA	x	13.44	3.03	0.5
Kp	g4205a, A42G, E104K, M182T	CATAGTCACCGGCTAGAT		0.26	5.53	0.55
Kp	g4205a, A42G, E104K, M182T	GAGAGGCTAAGGTGAAAC		0.25	4.93	0.53
Kp	g4205a, A42G, E104K, M182T	GCATCGCTCTCATGGGTA	x	0.24	5.31	0.53
Kp	g4205a, A42G, E104K, G238S	TATTTGAGTTATTAGTTC		17.35	2.8	0.53
Kp	g4205a, A42G, E104K, G238S	AGTGCAGGTTTAAATACT		15.66	3.22	0.54

Kp	g4205a, A42G, E104K, G238S	ACGTAAAGTAAGACTTCA	x	15.98	3.21	0.52
Kp	g4205a, A42G, M182T, G238S	TTAGCTTGGTTTCTGTCT		4.18	3.38	0.54
Kp	g4205a, A42G, M182T, G238S	GATAGGGTATATTGGCAC		3.33	4.53	0.54
Kp	g4205a, A42G, M182T, G238S	TAGCATCGGGTCAGGGCG	x	3.39	4.14	0.49
Kp	g4205a, E104K, M182T, G238S	ATATTTTACCGTCTTAAA		128.31	1.21	0.52
Kp	g4205a, E104K, M182T, G238S	GCCGCGGCGTGTGTGGTT		93.22	0.73	0.56
Kp	g4205a, E104K, M182T, G238S	GTAGTAGGTGTCTCAGAC	x	211.57	2.62	0.52
Kp	A42G, E104K, M182T, G238S	ACGAACTTTGCTTTCTTT		16.06	2.85	0.53
Kp	A42G, E104K, M182T, G238S	GAAAACATACGGCGTGGT		15.91	3.08	0.54
Kp	A42G, E104K, M182T, G238S	AGTCATGGCATTATGAAA	x	22.86	2.82	0.53
Kp	g4205a, A42G, E104K, M182T, G238S	TGAAGCACGTAAACTATC		220.64	2.1	0.53
Kp	g4205a, A42G, E104K, M182T, G238S	TTGGTTGCACCAGACATT		159.87	6.58	0.54
Kp	g4205a, A42G, E104K, M182T, G238S	CTGTTTTTGAACTTGAAG	x	13503.68	0.42	0.55
Se	Wild-type TEM-1	ATTCGTAAACTTCTGGTT		0.09	5.47	0.49
Se	Wild-type TEM-1	GAGTCGTGCATTGAGTTC		0.09	6.25	0.47
Se	Wild-type TEM-1	GATGGCCTTTTGCCGGTT	x	0.1	4.79	0.48
Se	g4205a	GTATTAGTTTTACTTTAG		0.12	4.03	0.49
Se	g4205a	TTGAGTCCCGAGGGTGGT		0.12	3.83	0.49
Se	g4205a	TACTTTTAAACTATAAGA	x	0.12	4.02	0.47
Se	A42G	CAGTGAGTCAGATATCTT		0.12	4.11	0.49
Se	A42G	GTAGCCATTTACTTCTGT		0.11	4.62	0.49
Se	A42G	TTCGCATTATATTCCGTC	x	0.1	6.09	0.46
Se	E104K	TTGGCGCCTTCTCTTCTG		0.34	3.49	0.49
Se	E104K	GGAGCAGTAGAGGTGGTA		0.3	3.86	0.49
Se	E104K	ATAAAGGATGTGACTGTA	x	0.32	4.82	0.46
Se	M182T	TCCAAAGGGTGGCACGAG		0.09	5.47	0.49
Se	M182T	TGTGATTTACACACGTCC		0.1	5.14	0.49

Se	M182T	CCTAGCAGCTCGTAAGAG	x	0.1	5.02	0.48
Se	G238S	CCAACTGTAAGCCTATTT		1.77	4.84	0.45
Se	G238S	CAGGGTCATACGAGCTTC		1.87	5.62	0.46
Se	G238S	ACTGCGTTTTAATATTTT	x	1.62	3.74	0.55
Se	g4205a, A42G	CATTCATATTAAAGTTTG		0.16	3.56	0.47
Se	g4205a, A42G	CAGGTTTTAGCATATGCC		0.16	3.01	0.52
Se	g4205a, A42G	CTCATTTTAGACTTCGTT	x	0.16	2.8	0.47
Se	g4205a, E104K	CGACAACGTATCAAGCTC		0.52	4.25	0.48
Se	g4205a, E104K	TCCGACCATTAAGGGTTA		0.5	4.3	0.48
Se	g4205a, E104K	GCTGGTCCGATCAGATAT	x	0.47	3.44	0.47
Se	g4205a, M182T	AGGGATCTGGAGTAGGTC		0.11	4.92	0.45
Se	g4205a, M182T	AATGTGCGTTAATAGATT		0.11	5.29	0.48
Se	g4205a, M182T	TATCATAGTGAGTTCCAT	x	0.13	3.19	0.5
Se	g4205a, G238S	ATAAGGTTTGTTTCCCTG		1.64	4.28	0.41
Se	g4205a, G238S	AATTTAAGTATAGAGGGG		1.73	5.51	0.4
Se	g4205a, G238S	TAGGTTAATTCTCGGTGA	x	1.63	3.84	0.4
Se	A42G, E104K	GTTTCCTCTAAAGATTTC		0.81	3.8	0.48
Se	A42G, E104K	TTTTTCCTCCGCTCTGGT		0.88	3.65	0.48
Se	A42G, E104K	ATTAGATTTATTAATATG	x	0.68	3.97	0.47
Se	A42G, M182T	AGAACTTGGTAACGGGGC		0.1	5.39	0.49
Se	A42G, M182T	TTGGGACCTCTTTGGGTA		0.1	5.3	0.5
Se	A42G, M182T	CGTACCCCTTGCTGGTGG	x	0.11	3.54	0.48
Se	A42G, G238S	CAGCTGGTTGGTTCTCTA		13.9	1.69	0.48
Se	A42G, G238S	ATAGTTATTTTGGAACTA		12.55	1.88	0.51
Se	A42G, G238S	ACTACAGTAATAGTGCAT	x	14.91	1.82	0.53
Se	E104K, M182T	GCGAGATGATTAGAGAGA		0.26	3.85	0.48
Se	E104K, M182T	GTGTGAGACGCAGTTTAG		0.36	3.12	0.49
Se	E104K, M182T	GCTTCCTTTATTTGTTTA	x	0.29	4.03	0.56
Se	E104K, G238S	TAACGCGAGTCGTAATCT		14.37	1.69	0.49
Se	E104K, G238S	GAGGGTGTGATTAGCAAT		14.11	1.7	0.48
Se	E104K, G238S	TGTATTGGTTAACGTTAC	x	15.92	1.63	0.54
Se	M182T, G238S	TATCCGTCCTCGGCAGAG		7.25	1.98	0.49
Se	M182T, G238S	TCCCTAGCATGGATTGGC		8.39	1.92	0.49
Se	M182T, G238S	GATCCGATGATAGTAGTT	x	7.93	1.58	0.51
Se	g4205a, A42G, E104K	ATAAATATGTGGTCCCTG		1.28	3.24	0.49
Se	g4205a, A42G, E104K	CACCATCCTACAACTAAA		1.45	3.07	0.48
Se	g4205a, A42G, E104K	AATCATTCAAATCGAAGA	x	1.37	3.31	0.45

Se	g4205a, A42G, M182T	TTAACACATGGTATTTAC		0.13	3.77	0.46
Se	g4205a, A42G, M182T	AAAGTTTGAGGAATAACG		0.12	4.31	0.47
Se	g4205a, A42G, M182T	TTGTTTATTTACCGGACT	x	0.16	3.22	0.47
Se	g4205a, A42G, G238S	ACAGGAGACGGTATCTTT		18.25	1.96	0.48
Se	g4205a, A42G, G238S	TTGAAATGCTTTCGGTTA		16.28	2.18	0.47
Se	g4205a, A42G, G238S	AAGTGTTCGCATTGCAAG	x	16.88	2.05	0.46
Se	g4205a, E104K, M182T	GCGTCCTCGAGTCTTTAC		0.5	4.3	0.47
Se	g4205a, E104K, M182T	TTTGGACCACTTTTCTGT		0.52	3.85	0.47
Se	g4205a, E104K, M182T	AGTCGTGTGGGGGCCTAC	x	0.59	4.19	0.49
Se	g4205a, E104K, G238S	AACGGTTGGACCGAGCGG		12.12	2.1	0.42
Se	g4205a, E104K, G238S	TTGCGGAAGTGGTCGTGG		12.44	2.21	0.42
Se	g4205a, E104K, G238S	CCATTTGATTTTAAGCTC	x	12.71	2.35	0.41
Se	g4205a, M182T, G238S	TTTGTTGCTCTTTCGATG		13	1.83	0.48
Se	g4205a, M182T, G238S	AACCTAATTCTTAACGGA		11.92	2.25	0.47
Se	g4205a, M182T, G238S	TCCGGTACGATTACAACG	x	14.89	1.91	0.47
Se	A42G, E104K, M182T	TTGCATAGTTTCATAATA		0.74	3.42	0.47
Se	A42G, E104K, M182T	GCAGTATGCGGAAAAGCT		0.75	3.31	0.48
Se	A42G, E104K, M182T	GTGCGAAAGCATTACACT	x	0.73	3.7	0.54
Se	A42G, E104K, G238S	AGCATGCTCTGCCGAGAA		77.61	2.26	0.47
Se	A42G, E104K, G238S	ACTGCTTAGCAGTTTGTC		75.2	2.11	0.48
Se	A42G, E104K, G238S	TCCACGTACATAAATGTT	x	67.54	2.18	0.49
Se	A42G, M182T, G238S	GGCAGGTGAATCTACCAG		17	1.74	0.46
Se	A42G, M182T, G238S	TTTAGCCCCATCACTAAC		16.99	2.03	0.47
Se	A42G, M182T, G238S	TTTGTGTAGGTACTATCC	x	13.99	1.96	0.47
Se	E104K, M182T, G238S	CAATATACTCTGTACTAA		81.03	2.19	0.48

Se	E104K, M182T, G238S	TAGTGCTTGTTCAGGGGT		80.42	2	0.48
Se	E104K, M182T, G238S	CATCTAATTTATTGGGTA	x	84.48	2.06	0.47
Se	g4205a, A42G, E104K, M182T	GCATCGCTCTCATGGGTA		1.27	3.43	0.48
Se	g4205a, A42G, E104K, M182T	GAGAGGCTAAGGTGAAAC		1.13	3.33	0.48
Se	g4205a, A42G, E104K, M182T	CATAGTCACCGGCTAGAT	x	1.42	2.79	0.47
Se	g4205a, A42G, E104K, G238S	ACGTAAAGTAAGACTTCA		86.27	2.31	0.46
Se	g4205a, A42G, E104K, G238S	AGTGCAGGTTTAAATACT		82.6	2.47	0.45
Se	g4205a, A42G, E104K, G238S	TATTTGAGTTATTAGTTC	x	99.11	1.82	0.45
Se	g4205a, A42G, M182T, G238S	TTAGCTTGGTTTCTGTCT		26.49	2.16	0.47
Se	g4205a, A42G, M182T, G238S	GATAGGGTATATTGGCAC		23.65	1.91	0.46
Se	g4205a, A42G, M182T, G238S	TAGCATCGGGTCAGGGCG	x	27.5	1.66	0.52
Se	g4205a, E104K, M182T, G238S	GCCGCGGCGTGTGTGGTT		149.32	2.01	0.49
Se	g4205a, E104K, M182T, G238S	GTAGTAGGTGTCTCAGAC		167.72	3.91	0.48
Se	g4205a, E104K, M182T, G238S	ATATTTTACCGTCTTAAA	x	164.9	2.96	0.45
Se	A42G, E104K, M182T, G238S	GAAAACATACGGCGTGGT		106.57	2.25	0.48
Se	A42G, E104K, M182T, G238S	AGTCATGGCATTATGAAA		136.69	2.39	0.47
Se	A42G, E104K, M182T, G238S	ACGAACTTTGCTTTCTTT	x	27179.24	0.03	0.83
Se	g4205a, A42G, E104K, M182T, G238S	CTGTTTTTGAACTTGAAG		3009.24	0.81	0.48
Se	g4205a, A42G, E104K, M182T, G238S	TTGGTTGCACCAGACATT		1026	1.52	0.46
Se	g4205a, A42G, E104K, M182T, G238S	TGAAGCACGTAAACTATC	x	1162.37	1.42	0.49

SI Table 10 : Specific datasets and parameters used in the evolutionary simulations. The same population size (1,000 individuals) was used in each treatment.

Relevant figure	Focal gene	Focal host	Transient host	Time steps	HGT middle period	Mutation rate	Replicates
Figure 4a, Figure 4d, SI Figure 2-4	bla_{TEM}	Ec	*None	60	*None	5×10^{-5}	1000
Figure 4b, Figure 4d, SI Figure 2-4	bla_{TEM}	Ec	Kp	60	21-40	5×10^{-5}	1000
Figure 4c, Figure 4d, SI Figure 2-4	bla_{TEM}	Ec	Se	60	21-40	5×10^{-5}	1000
Figure 4e, Figure 4h, SI Figure 2-4	bla_{TEM}	Kp	*None	60	*None	5×10^{-5}	1000
Figure 4f, Figure 4h, SI Figure 2-4	bla_{TEM}	Kp	Ec	60	21-40	5×10^{-5}	1000
Figure 4g, Figure 4h, SI Figure 2-4	bla_{TEM}	Kp	Se	60	21-40	5×10^{-5}	1000
Figure 4i, Figure 4l, SI Figure 2-4	bla_{TEM}	Se	*None	60	*None	5×10^{-5}	1000
Figure 4j, Figure 4l, SI Figure 2-4	bla_{TEM}	Se	Ec	60	21-40	5×10^{-5}	1000
Figure 4k, Figure 4l, SI Figure 2-4	bla_{TEM}	Se	Kp	60	21-40	5×10^{-5}	1000
SI Figure 2a	bla_{TEM}	Ec	*None	60	*None	1×10^{-6}	1000
SI Figure 2a	bla_{TEM}	Ec	Kp	60	21-40	1×10^{-6}	1000
SI Figure 2a	bla_{TEM}	Ec	Se	60	21-40	1×10^{-6}	1000
SI Figure 2b	bla_{TEM}	Kp	*None	60	*None	1×10^{-6}	1000
SI Figure 2b	bla_{TEM}	Kp	Ec	60	21-40	1×10^{-6}	1000
SI Figure 2b	bla_{TEM}	Kp	Se	60	21-40	1×10^{-6}	1000
SI Figure 2c	bla_{TEM}	Se	*None	60	*None	1×10^{-6}	1000
SI Figure 2c	bla_{TEM}	Se	Ec	60	21-40	1×10^{-6}	1000
SI Figure 2c	bla_{TEM}	Se	Kp	60	21-40	1×10^{-6}	1000
SI Figure 2a	bla_{TEM}	Ec	*None	60	*None	5×10^{-6}	1000
SI Figure 2a	bla_{TEM}	Ec	Kp	60	21-40	5×10^{-6}	1000
SI Figure 2a	bla_{TEM}	Ec	Se	60	21-40	5×10^{-6}	1000
SI Figure 2b	bla_{TEM}	Kp	*None	60	*None	5×10^{-6}	1000
SI Figure 2b	bla_{TEM}	Kp	Ec	60	21-40	5×10^{-6}	1000
SI Figure 2b	bla_{TEM}	Kp	Se	60	21-40	5×10^{-6}	1000
SI Figure 2c	bla_{TEM}	Se	*None	60	*None	5×10^{-6}	1000
SI Figure 2c	bla_{TEM}	Se	Ec	60	21-40	5×10^{-6}	1000
SI Figure 2c	bla_{TEM}	Se	Kp	60	21-40	5×10^{-6}	1000
SI Figure 2a	bla_{TEM}	Ec	*None	60	*None	1×10^{-5}	1000
SI Figure 2a	bla_{TEM}	Ec	Kp	60	21-40	1×10^{-5}	1000

SI Figure 2a	*bla*$_{TEM}$	Ec	Se	60	21-40	1 x 10^{-5}	1000
SI Figure 2b	*bla*$_{TEM}$	Kp	*None	60	*None	1 x 10^{-5}	1000
SI Figure 2b	*bla*$_{TEM}$	Kp	Ec	60	21-40	1 x 10^{-5}	1000
SI Figure 2b	*bla*$_{TEM}$	Kp	Se	60	21-40	1 x 10^{-5}	1000
SI Figure 2c	*bla*$_{TEM}$	Se	*None	60	*None	1 x 10^{-5}	1000
SI Figure 2c	*bla*$_{TEM}$	Se	Ec	60	21-40	1 x 10^{-5}	1000
SI Figure 2c	*bla*$_{TEM}$	Se	Kp	60	21-40	1 x 10^{-5}	1000
SI Figure 2a	*bla*$_{TEM}$	Ec	*None	60	*None	1 x 10^{-4}	1000
SI Figure 2a	*bla*$_{TEM}$	Ec	Kp	60	21-40	1 x 10^{-4}	1000
SI Figure 2a	*bla*$_{TEM}$	Ec	Se	60	21-40	1 x 10^{-4}	1000
SI Figure 2b	*bla*$_{TEM}$	Kp	*None	60	*None	1 x 10^{-4}	1000
SI Figure 2b	*bla*$_{TEM}$	Kp	Ec	60	21-40	1 x 10^{-4}	1000
SI Figure 2b	*bla*$_{TEM}$	Kp	Se	60	21-40	1 x 10^{-4}	1000
SI Figure 2c	*bla*$_{TEM}$	Se	*None	60	*None	1 x 10^{-4}	1000
SI Figure 2c	*bla*$_{TEM}$	Se	Ec	60	21-40	1 x 10^{-4}	1000
SI Figure 2c	*bla*$_{TEM}$	Se	Kp	60	21-40	1 x 10^{-4}	1000
SI Figure 2a	*bla*$_{TEM}$	Ec	*None	60	*None	5 x 10^{-4}	1000
SI Figure 2a	*bla*$_{TEM}$	Ec	Kp	60	21-40	5 x 10^{-4}	1000
SI Figure 2a	*bla*$_{TEM}$	Ec	Se	60	21-40	5 x 10^{-4}	1000
SI Figure 2b	*bla*$_{TEM}$	Kp	*None	60	*None	5 x 10^{-4}	1000
SI Figure 2b	*bla*$_{TEM}$	Kp	Ec	60	21-40	5 x 10^{-4}	1000
SI Figure 2b	*bla*$_{TEM}$	Kp	Se	60	21-40	5 x 10^{-4}	1000
SI Figure 2c	*bla*$_{TEM}$	Se	*None	60	*None	5 x 10^{-4}	1000
SI Figure 2c	*bla*$_{TEM}$	Se	Ec	60	21-40	5 x 10^{-4}	1000
SI Figure 2c	*bla*$_{TEM}$	Se	Kp	60	21-40	5 x 10^{-4}	1000
SI Figure 3a	*bla*$_{TEM}$	Ec	*None	30	*None	5 x 10^{-5}	1000
SI Figure 3a	*bla*$_{TEM}$	Ec	Kp	30	21-40	5 x 10^{-5}	1000
SI Figure 3a	*bla*$_{TEM}$	Ec	Se	30	21-40	5 x 10^{-5}	1000
SI Figure 3b	*bla*$_{TEM}$	Kp	*None	30	*None	5 x 10^{-5}	1000
SI Figure 3b	*bla*$_{TEM}$	Kp	Ec	30	21-40	5 x 10^{-5}	1000
SI Figure 3b	*bla*$_{TEM}$	Kp	Se	30	21-40	5 x 10^{-5}	1000
SI Figure 3c	*bla*$_{TEM}$	Se	*None	30	*None	5 x 10^{-5}	1000
SI Figure 3c	*bla*$_{TEM}$	Se	Ec	30	21-40	5 x 10^{-5}	1000
SI Figure 3c	*bla*$_{TEM}$	Se	Kp	30	21-40	5 x 10^{-5}	1000
SI Figure 3a	*bla*$_{TEM}$	Ec	*None	45	*None	5 x 10^{-5}	1000
SI Figure 3a	*bla*$_{TEM}$	Ec	Kp	45	21-40	5 x 10^{-5}	1000
SI Figure 3a	*bla*$_{TEM}$	Ec	Se	45	21-40	5 x 10^{-5}	1000
SI Figure 3b	*bla*$_{TEM}$	Kp	*None	45	*None	5 x 10^{-5}	1000
SI Figure 3b	*bla*$_{TEM}$	Kp	Ec	45	21-40	5 x 10^{-5}	1000

SI Figure 3b	bla_{TEM}	Kp	Se	45	21-40	5 x 10^{-5}	1000
SI Figure 3c	bla_{TEM}	Se	*None	45	*None	5 x 10^{-5}	1000
SI Figure 3c	bla_{TEM}	Se	Ec	45	21-40	5 x 10^{-5}	1000
SI Figure 3c	bla_{TEM}	Se	Kp	45	21-40	5 x 10^{-5}	1000
SI Figure 3a	bla_{TEM}	Ec	*None	75	*None	5 x 10^{-5}	1000
SI Figure 3a	bla_{TEM}	Ec	Kp	75	21-40	5 x 10^{-5}	1000
SI Figure 3a	bla_{TEM}	Ec	Se	75	21-40	5 x 10^{-5}	1000
SI Figure 3b	bla_{TEM}	Kp	*None	75	*None	5 x 10^{-5}	1000
SI Figure 3b	bla_{TEM}	Kp	Ec	75	21-40	5 x 10^{-5}	1000
SI Figure 3b	bla_{TEM}	Kp	Se	75	21-40	5 x 10^{-5}	1000
SI Figure 3c	bla_{TEM}	Se	*None	75	*None	5 x 10^{-5}	1000
SI Figure 3c	bla_{TEM}	Se	Ec	75	21-40	5 x 10^{-5}	1000
SI Figure 3c	bla_{TEM}	Se	Kp	75	21-40	5 x 10^{-5}	1000
SI Figure 3a	bla_{TEM}	Ec	*None	90	*None	5 x 10^{-5}	1000
SI Figure 3a	bla_{TEM}	Ec	Kp	90	21-40	5 x 10^{-5}	1000
SI Figure 3a	bla_{TEM}	Ec	Se	90	21-40	5 x 10^{-5}	1000
SI Figure 3b	bla_{TEM}	Kp	*None	90	*None	5 x 10^{-5}	1000
SI Figure 3b	bla_{TEM}	Kp	Ec	90	21-40	5 x 10^{-5}	1000
SI Figure 3b	bla_{TEM}	Kp	Se	90	21-40	5 x 10^{-5}	1000
SI Figure 3c	bla_{TEM}	Se	*None	90	*None	5 x 10^{-5}	1000
SI Figure 3c	bla_{TEM}	Se	Ec	90	21-40	5 x 10^{-5}	1000
SI Figure 3c	bla_{TEM}	Se	Kp	90	21-40	5 x 10^{-5}	1000
SI Figure 4a	bla_{TEM}	Ec	*None	60	*None	5 x 10^{-5}	100
SI Figure 4a	bla_{TEM}	Ec	Kp	60	21-40	5 x 10^{-5}	100
SI Figure 4a	bla_{TEM}	Ec	Se	60	21-40	5 x 10^{-5}	100
SI Figure 4b	bla_{TEM}	Kp	*None	60	*None	5 x 10^{-5}	100
SI Figure 4b	bla_{TEM}	Kp	Ec	60	21-40	5 x 10^{-5}	100
SI Figure 4b	bla_{TEM}	Kp	Se	60	21-40	5 x 10^{-5}	100
SI Figure 4c	bla_{TEM}	Se	*None	60	*None	5 x 10^{-5}	100
SI Figure 4c	bla_{TEM}	Se	Ec	60	21-40	5 x 10^{-5}	100
SI Figure 4c	bla_{TEM}	Se	Kp	60	21-40	5 x 10^{-5}	100
SI Figure 4a	bla_{TEM}	Ec	*None	60	*None	5 x 10^{-5}	500
SI Figure 4a	bla_{TEM}	Ec	Kp	60	21-40	5 x 10^{-5}	500
SI Figure 4a	bla_{TEM}	Ec	Se	60	21-40	5 x 10^{-5}	500
SI Figure 4b	bla_{TEM}	Kp	*None	60	*None	5 x 10^{-5}	500
SI Figure 4b	bla_{TEM}	Kp	Ec	60	21-40	5 x 10^{-5}	500
SI Figure 4b	bla_{TEM}	Kp	Se	60	21-40	5 x 10^{-5}	500
SI Figure 4c	bla_{TEM}	Se	*None	60	*None	5 x 10^{-5}	500
SI Figure 4c	bla_{TEM}	Se	Ec	60	21-40	5 x 10^{-5}	500

SI Figure 4c	*bla*$_{TEM}$	Se	Kp	60	21-40	5 x 10^{-5}	500
SI Figure 4a	*bla*$_{TEM}$	Ec	*None	60	*None	5 x 10^{-5}	5000
SI Figure 4a	*bla*$_{TEM}$	Ec	Kp	60	21-40	5 x 10^{-5}	5000
SI Figure 4a	*bla*$_{TEM}$	Ec	Se	60	21-40	5 x 10^{-5}	5000
SI Figure 4b	*bla*$_{TEM}$	Kp	*None	60	*None	5 x 10^{-5}	5000
SI Figure 4b	*bla*$_{TEM}$	Kp	Ec	60	21-40	5 x 10^{-5}	5000
SI Figure 4b	*bla*$_{TEM}$	Kp	Se	60	21-40	5 x 10^{-5}	5000
SI Figure 4c	*bla*$_{TEM}$	Se	*None	60	*None	5 x 10^{-5}	5000
SI Figure 4c	*bla*$_{TEM}$	Se	Ec	60	21-40	5 x 10^{-5}	5000
SI Figure 4c	*bla*$_{TEM}$	Se	Kp	60	21-40	5 x 10^{-5}	5000
SI Figure 4a	*bla*$_{TEM}$	Ec	*None	60	*None	5 x 10^{-5}	10000
SI Figure 4a	*bla*$_{TEM}$	Ec	Kp	60	21-40	5 x 10^{-5}	10000
SI Figure 4a	*bla*$_{TEM}$	Ec	Se	60	21-40	5 x 10^{-5}	10000
SI Figure 4b	*bla*$_{TEM}$	Kp	*None	60	*None	5 x 10^{-5}	10000
SI Figure 4b	*bla*$_{TEM}$	Kp	Ec	60	21-40	5 x 10^{-5}	10000
SI Figure 4b	*bla*$_{TEM}$	Kp	Se	60	21-40	5 x 10^{-5}	10000
SI Figure 4c	*bla*$_{TEM}$	Se	*None	60	*None	5 x 10^{-5}	10000
SI Figure 4c	*bla*$_{TEM}$	Se	Ec	60	21-40	5 x 10^{-5}	10000
SI Figure 4c	*bla*$_{TEM}$	Se	Kp	60	21-40	5 x 10^{-5}	10000

References

Alonso-Del Valle A, León-Sampedro R, Rodríguez-Beltrán J, DelaFuente J, Hernández-García M, Ruiz-Garbajosa P, Cantón R, Peña-Miller R, San Millán A. 2021. Variability of plasmid fitness effects contributes to plasmid persistence in bacterial communities. *Nat. Commun.* [Internet] 12:2653. Available from: http://dx.doi.org/10.1038/s41467-021-22849-y

Apjok G, Boross G, Nyerges Á, Fekete G, Lázár V, Papp B, Pál C, Csörgő B. 2019. Limited Evolutionary Conservation of the Phenotypic Effects of Antibiotic Resistance Mutations. *Mol. Biol. Evol.* [Internet] 36:1601–1611. Available from: http://dx.doi.org/10.1093/molbev/msz109

Artemova T, Gerardin Y, Dudley C, Vega NM, Gore J. 2015. Isolated cell behavior drives the evolution of antibiotic resistance. *Mol. Syst. Biol.* [Internet] 11:822. Available from: http://dx.doi.org/10.15252/msb.20145888

Bank C. 2022. Epistasis and Adaptation on Fitness Landscapes. *Annu. Rev. Ecol. Evol. Syst.* [Internet] 53:457–479. Available from: https://doi.org/10.1146/annurev-ecolsys-102320-112153

Bank C, Matuszewski S, Hietpas RT, Jensen JD. 2016. On the (un)predictability of a large intragenic fitness landscape. *Proc. Natl. Acad. Sci. U. S. A.* [Internet] 113:14085–14090. Available from: http://dx.doi.org/10.1073/pnas.1612676113

Barlow M. 2009. What Antimicrobial Resistance Has Taught Us About Horizontal Gene Transfer. In: Gogarten MB, Gogarten JP, Olendzenski LC, editors. Horizontal Gene Transfer: Genomes in Flux. Totowa, NJ: Humana Press. p. 397–411. Available from: https://doi.org/10.1007/978-1-60327-853-9_23

Barlow M, Hall BG. 2002. Predicting evolutionary potential: in vitro evolution accurately reproduces natural evolution of the tem beta-lactamase. *Genetics* [Internet] 160:823–832. Available from: http://dx.doi.org/10.1093/genetics/160.3.823

Benz F, Hall AR. 2023. Host-specific plasmid evolution explains the variable spread of clinical antibiotic-resistance plasmids. *Proc. Natl. Acad. Sci. U. S. A.* [Internet] 120:e2212147120. Available from: http://dx.doi.org/10.1073/pnas.2212147120

Chevin L-M. 2010. On measuring selection in experimental evolution. *Biol. Lett.* [Internet] 7:210–213. Available from: https://doi.org/10.1098/rsbl.2010.0580

Concepción-Acevedo J, Weiss HN, Chaudhry WN, Levin BR. 2015. Malthusian Parameters as Estimators of the Fitness of Microbes: A Cautionary Tale about the Low Side of High Throughput. *PLoS One* [Internet] 10:e0126915. Available from: http://dx.doi.org/10.1371/journal.pone.0126915

Das SG, Direito SO, Waclaw B, Allen RJ, Krug J. 2020. Predictable properties of fitness landscapes induced by adaptational tradeoffs. *Elife* [Internet] 9. Available from: http://dx.doi.org/10.7554/eLife.55155

De Gelder L, Ponciano JM, Joyce P, Top EM. 2007. Stability of a promiscuous plasmid in different hosts: no guarantee for a long-term relationship. *Microbiology* [Internet] 153:452–463. Available from: http://dx.doi.org/10.1099/mic.0.2006/001784-0

Dimitriu T, Marchant L, Buckling A, Raymond B. 2019. Bacteria from natural populations transfer plasmids mostly towards their kin. *Proc. Biol. Sci.* [Internet] 286:20191110. Available from: http://dx.doi.org/10.1098/rspb.2019.1110

Draghi JA, Plotkin JB. 2013. Selection biases the prevalence and type of epistasis along adaptive trajectories. *Evolution* [Internet] 67:3120–3131. Available from: http://dx.doi.org/10.1111/evo.12192

Dunn S, Carrilero L, Brockhurst M, McNally A. 2021. Limited and Strain-Specific Transcriptional and Growth Responses to Acquisition of a Multidrug Resistance Plasmid in Genetically Diverse Escherichia coli Lineages. *mSystems* [Internet] 6. Available from: http://dx.doi.org/10.1128/mSystems.00083-21

Durfee T, Nelson R, Baldwin S, Plunkett G 3rd, Burland V, Mau B, Petrosino JF, Qin X, Muzny DM, Ayele M, et al. 2008. The complete genome sequence of Escherichia coli DH10B: insights into the biology of a laboratory workhorse. *J. Bacteriol.* [Internet] 190:2597–2606. Available from: http://dx.doi.org/10.1128/JB.01695-07

Eguchi Y, Bilolikar G, Geiler-Samerotte K. 2019. Why and how to study genetic changes with context-dependent effects. *Curr. Opin. Genet. Dev.* [Internet] 58–59:95–102. Available from: http://dx.doi.org/10.1016/j.gde.2019.08.003

Flynn KM, Cooper TF, Moore FB-G, Cooper VS. 2013. The environment affects epistatic interactions to alter the topology of an empirical fitness landscape. *PLoS Genet.* [Internet] 9:e1003426. Available from: http://dx.doi.org/10.1371/journal.pgen.1003426

Fowler DM, Fields S. 2014. Deep mutational scanning: a new style of protein science. *Nat. Methods* [Internet] 11:801–807. Available from: http://dx.doi.org/10.1038/nmeth.3027

Fragata I, Blanckaert A, Dias Louro MA, Liberles DA, Bank C. 2019. Evolution in the light of fitness landscape theory. *Trends Ecol. Evol.* [Internet] 34:69–82. Available from: http://dx.doi.org/10.1016/j.tree.2018.10.009

Gama JA, Kloos J, Johnsen PJ, Samuelsen Ø. 2020. Host dependent maintenance of a blaNDM-1-encoding plasmid in clinical Escherichia coli isolates. *Sci. Rep.* [Internet] 10:9332. Available from: http://dx.doi.org/10.1038/s41598-020-66239-8

Gillespie JH. 1984. Molecular Evolution Over the Mutational Landscape. *Evolution* [Internet] 38:1116–1129. Available from: http://www.jstor.org/stable/2408444

Grewal RK, Sinha S, Roy S. 2018. Topologically Inspired Walks on Randomly Connected Landscapes With Correlated Fitness. *Frontiers in Physics* [Internet] 6. Available from: https://www.frontiersin.org/articles/10.3389/fphy.2018.00138

Guerrero RF, Scarpino SV, Rodrigues JV, Hartl DL, Ogbunugafor CB. 2019. Proteostasis Environment Shapes Higher-Order Epistasis Operating on Antibiotic Resistance. *Genetics* [Internet] 212:565–575. Available from: http://dx.doi.org/10.1534/genetics.119.302138

Gullberg E, Cao S, Berg OG, Ilbäck C, Sandegren L, Hughes D, Andersson DI. 2011. Selection of resistant bacteria at very low antibiotic concentrations. *PLoS Pathog.* [Internet] 7:e1002158. Available from: http://dx.doi.org/10.1371/journal.ppat.1002158

Gupta A, Zaman L, Strobel HM, Gallie J, Burmeister AR, Kerr B, Tamar ES, Kishony R, Meyer JR. 2022. Host-parasite coevolution promotes innovation through deformations in fitness landscapes. *Elife* [Internet] 11. Available from: http://dx.doi.org/10.7554/eLife.76162

Heath KD. 2010. Intergenomic epistasis and coevolutionary constraint in plants and rhizobia. *Evolution* [Internet] 64:1446–1458. Available from: http://dx.doi.org/10.1111/j.1558-5646.2009.00913.x

Jain R, Rivera MC, Lake JA. 1999. Horizontal gene transfer among genomes: the complexity hypothesis. *Proc. Natl. Acad. Sci. U. S. A.* [Internet] 96:3801–3806. Available from: http://dx.doi.org/10.1073/pnas.96.7.3801

Jordt H, Stalder T, Kosterlitz O, Ponciano JM, Top EM, Kerr B. 2020. Coevolution of host-plasmid pairs facilitates the emergence of novel multidrug resistance. *Nat Ecol Evol* [Internet] 4:863–869. Available from: http://dx.doi.org/10.1038/s41559-020-1170-1

Kosterlitz O, Tirado AM, Wate C, Elg C, Bozic I, Top EM, Kerr B. 2022. Estimating the transfer rates of bacterial plasmids with an adapted Luria-Delbrück fluctuation analysis. *PLoS Biol.* [Internet] 20:e3001732. Available from: http://dx.doi.org/10.1371/journal.pbio.3001732

Lindsey HA, Gallie J, Taylor S, Kerr B. 2013. Evolutionary rescue from extinction is contingent on a lower rate of environmental change. *Nature* [Internet] 494:463–467. Available from: http://dx.doi.org/10.1038/nature11879

McClelland M, Sanderson KE, Spieth J, Clifton SW, Latreille P, Courtney L, Porwollik S, Ali J, Dante M, Du F, et al. 2001. Complete genome sequence of Salmonella enterica serovar Typhimurium LT2. *Nature* [Internet] 413:852–856. Available from: http://dx.doi.org/10.1038/35101614

Mustonen V, Lässig M. 2009. From fitness landscapes to seascapes: non-equilibrium dynamics of selection and adaptation. *Trends Genet.* [Internet] 25:111–119. Available from: http://dx.doi.org/10.1016/j.tig.2009.01.002

Novick A, Doolittle WF. 2020. Horizontal persistence and the complexity hypothesis. *Biol. Philos.* [Internet] 35. Available from: http://link.springer.com/10.1007/s10539-019-9727-6

Ogbunugafor CB, Eppstein MJ. 2019. Genetic Background Modifies the Topography of a Fitness Landscape, Influencing the Dynamics of Adaptive Evolution. *IEEE Access* [Internet] 7:113675–113683. Available from: http://dx.doi.org/10.1109/ACCESS.2019.2935911

Ogbunugafor CB, Wylie CS, Diakite I, Weinreich DM, Hartl DL. 2016. Adaptive Landscape by Environment Interactions Dictate Evolutionary Dynamics in Models of Drug Resistance. *PLoS Comput. Biol.* [Internet] 12:e1004710. Available from: http://dx.doi.org/10.1371/journal.pcbi.1004710

Orr HA. 2002. The population genetics of adaptation: the adaptation of DNA sequences. *Evolution* [Internet] 56:1317–1330. Available from: http://dx.doi.org/10.1111/j.0014-3820.2002.tb01446.x

Orr HA. 2005. The genetic theory of adaptation: a brief history. *Nat. Rev. Genet.* [Internet] 6:119–127. Available from: http://dx.doi.org/10.1038/nrg1523

Packer MS, Liu DR. 2015. Methods for the directed evolution of proteins. *Nat. Rev. Genet.* [Internet] 16:379–394. Available from: http://dx.doi.org/10.1038/nrg3927

Redondo-Salvo S, Fernández-López R, Ruiz R, Vielva L, de Toro M, Rocha EPC, Garcillán-Barcia MP, de la Cruz F. 2020. Pathways for horizontal gene transfer in bacteria revealed by a global map of their plasmids. *Nat. Commun.* [Internet] 11:3602. Available from: http://dx.doi.org/10.1038/s41467-020-17278-2

Ritz C, Baty F, Streibig JC, Gerhard D. 2015. Dose-Response Analysis Using R. *PLoS One* [Internet] 10:e0146021. Available from: http://dx.doi.org/10.1371/journal.pone.0146021

Salverda MLM, De Visser JAGM, Barlow M. 2010. Natural evolution of TEM-1 β-lactamase: experimental reconstruction and clinical relevance. *FEMS Microbiol. Rev.* [Internet] 34:1015–1036. Available from: http://dx.doi.org/10.1111/j.1574-6976.2010.00222.x

Salverda MLM, Dellus E, Gorter FA, Debets AJM, van der Oost J, Hoekstra RF, Tawfik DS, de Visser JAGM. 2011. Initial mutations direct alternative pathways of protein evolution. *PLoS Genet.* [Internet] 7:e1001321. Available from: http://dx.doi.org/10.1371/journal.pgen.1001321

Salverda MLM, Koomen J, Koopmanschap B, Zwart MP, de Visser JAGM. 2017. Adaptive benefits from small mutation supplies in an antibiotic resistance enzyme. *Proc. Natl. Acad. Sci. U. S. A.* [Internet] 114:12773–12778. Available from: http://dx.doi.org/10.1073/pnas.1712999114

Sanchez A, Bajic D, Diaz-Colunga J, Skwara A, Vila JCC, Kuehn S. 2023. The community-function landscape of microbial consortia. *Cell Syst* [Internet] 14:122–134. Available from: http://dx.doi.org/10.1016/j.cels.2022.12.011

Sánchez Á, Vila JCC, Chang C-Y, Diaz-Colunga J, Estrela S, Rebolleda-Gomez M. 2021. Directed Evolution of Microbial Communities. *Annu. Rev. Biophys.* [Internet] 50:323–341. Available from: http://dx.doi.org/10.1146/annurev-biophys-101220-072829

Schenk MF, Zwart MP, Hwang S, Ruelens P, Severing E, Krug J, de Visser JAGM. 2022. Population size mediates the contribution of high-rate and large-benefit mutations to parallel evolution. *Nat Ecol Evol* [Internet] 6:439–447. Available from: http://dx.doi.org/10.1038/s41559-022-01669-3

Sørensen MES, Wood AJ, Cameron DD, Brockhurst MA. 2021. Rapid compensatory evolution can rescue low fitness symbioses following partner switching. *Curr. Biol.* [Internet] 31:3721-3728.e4. Available from: http://dx.doi.org/10.1016/j.cub.2021.06.034

Tanaka MM, Godfrey-Smith P, Kerr B. 2020. The Dual Landscape Model of Adaptation and Niche Construction. *Philos. Sci.* [Internet] 87:478–498. Available from:

https://www.cambridge.org/core/services/aop-cambridge-core/content/view/21BDC83AF25AA863C731D2D1CE2C8358/S0031824800015968a.pdf/div-class-title-the-dual-landscape-model-of-adaptation-and-niche-construction-div.pdf

Toprak E, Veres A, Michel J-B, Chait R, Hartl DL, Kishony R. 2011. Evolutionary paths to antibiotic resistance under dynamically sustained drug selection. *Nat. Genet.* [Internet] 44:101–105. Available from: http://dx.doi.org/10.1038/ng.1034

de Visser JAGM, Krug J. 2014. Empirical fitness landscapes and the predictability of evolution. *Nat. Rev. Genet.* [Internet] 15:480–490. Available from: http://dx.doi.org/10.1038/nrg3744

de Vos MGJ, Dawid A, Sunderlikova V, Tans SJ. 2015. Breaking evolutionary constraint with a tradeoff ratchet. *Proc. Natl. Acad. Sci. U. S. A.* [Internet] 112:14906–14911. Available from: http://dx.doi.org/10.1073/pnas.1510282112

de Vos MGJ, Poelwijk FJ, Battich N, Ndika JDT, Tans SJ. 2013. Environmental dependence of genetic constraint. *PLoS Genet.* [Internet] 9:e1003580. Available from: http://dx.doi.org/10.1371/journal.pgen.1003580

Wade MJ. 2007. The co-evolutionary genetics of ecological communities. *Nat. Rev. Genet.* [Internet] 8:185–195. Available from: http://dx.doi.org/10.1038/nrg2031

Weinreich DM, Delaney NF, Depristo MA, Hartl DL. 2006. Darwinian evolution can follow only very few mutational paths to fitter proteins. *Science* [Internet] 312:111–114. Available from: http://dx.doi.org/10.1126/science.1123539

Weinreich DM, Lan Y, Wylie CS, Heckendorn RB. 2013. Should evolutionary geneticists worry about higher-order epistasis? *Curr. Opin. Genet. Dev.* [Internet] 23:700–707. Available from: http://dx.doi.org/10.1016/j.gde.2013.10.007

Wiegand I, Hilpert K, Hancock REW. 2008. Agar and broth dilution methods to determine the minimal inhibitory concentration (MIC) of antimicrobial substances. *Nat. Protoc.* [Internet] 3:163–175. Available from: http://dx.doi.org/10.1038/nprot.2007.521

Wright S. 1932. The roles of mutation, inbreeding, crossbreeding and selection in evolution, Proceedings of the Sixth International Congress of Genetics. *Proc Sixth Int Congr Genet* [Internet] 1:356. Available from: https://cir.nii.ac.jp/crid/1373101967230580356

Xie L, Yuan AE, Shou W. 2019. Simulations reveal challenges to artificial community selection and possible strategies for success. *PLoS Biol.* [Internet] 17:e3000295. Available from: http://dx.doi.org/10.1371/journal.pbio.3000295

Yi X, Dean AM. 2019. Adaptive Landscapes in the Age of Synthetic Biology. *Mol. Biol. Evol.* [Internet] 36:890–907. Available from: http://dx.doi.org/10.1093/molbev/msz004

Zhao L, Liu Z, Levy SF, Wu S. 2018. Bartender: a fast and accurate clustering algorithm to count barcode reads. *Bioinformatics* [Internet] 34:739–747. Available from: http://dx.doi.org/10.1093/bioinformatics/btx655
